# Disentangling Electronic and Geometric Effects in Ternary NiFeX Layered Double Hydroxide Catalysts for Oxygen Evolution

**DOI:** 10.1021/jacs.6c12678

**Published:** 2026-09-07

**Authors:** D. Choi, P. W. Buchheister, R. Somaiya, D. Ligay, G. Podbielski, J. Lu, P. Schröer, E. Bumenn, I. Galkina, F. Scheepers, Z. Brejwo, A. Hartmann, B. Paul, T. N. Thanh, M. Klingenhof, A. Karl, E. Jodat, R.-A. Eichel, Z. Zeng, F. Dionigi, P. Strasser

**Affiliations:** † Electrochemical Energy, Catalysis, and Materials Science Laboratory, Department of Chemistry, Chemical Engineering Division, Technical University Berlin, Straße des 17. Juni 124, 10623 Berlin, Germany; ‡ School of Physics and Electronics, 12569 Hunan University, Changsha 410082, China; § Tarpo Department of Chemistry, Davidson School of Chemical Engineering, School of Materials Engineering, Institute of Physical AI, 311308 Purdue University, West Lafayette, Indiana 47907, United States; ∥ Institute of Energy Technologies, IET-1: Fundamental Electrochemistry, Forschungszentrum Jülich GmbH, Wilhelm-Johnen-Straße, 52428 Jülich, Germany; ⊥ Institute of Energy Technologies, IET-4: Electrochemical Process Engineering, Forschungszentrum Jülich GmbH, Wilhelm-Johnen-Straße, 52428 Jülich, Germany

## Abstract

Ternary NiFeX layered double hydroxide (LDH) electrocatalysts incorporating high-valence metals X from groups 5 and 6 have shown improved performance for the alkaline oxygen evolution reaction (OER), yet the molecular origin has remained speculative. Here, we synthesize a comprehensive series of phase-pure Ni_a_Fe_1_X_1_ LDHs (X = V, Nb, Ta, Cr, Mo, W) with controlled stoichiometry and unravel distinct catalytic enhancement mechanisms for different metals X. By combining impedance spectroscopy-based real ECSA determination, in situ high-energy resolution fluorescence detected X-ray absorption spectroscopy (HERFD-XAS), and density functional theory (DFT) calculations, we show that the geometric activity improvements of NiFeMo and NiFeV LDH arise from fundamentally different mechanisms: increased electrochemically active surface area for Mo, and a previously overlooked electronic promotional effect for V. In situ HERFD-XAS reveals that V uniquely modifies the activation of the NiFe LDH host, shifting the Ni K-edge prepeak of the active γ-phase to lower energies. DFT calculations demonstrate that surface V^5+^ acts as an electronic modulator at the V^5+^–OH–Ni^2+^ bridge site, enabling a favorable Ni-based redox pathway and lowering the overpotential to 0.37 V—an improvement of 0.08 V over γ-NiFe LDH. Both catalysts were validated in 25 cm^2^ anion exchange membrane electrolyzer cells, confirming their promise for large-scale alkaline water electrolysis.

## Graphical abstract

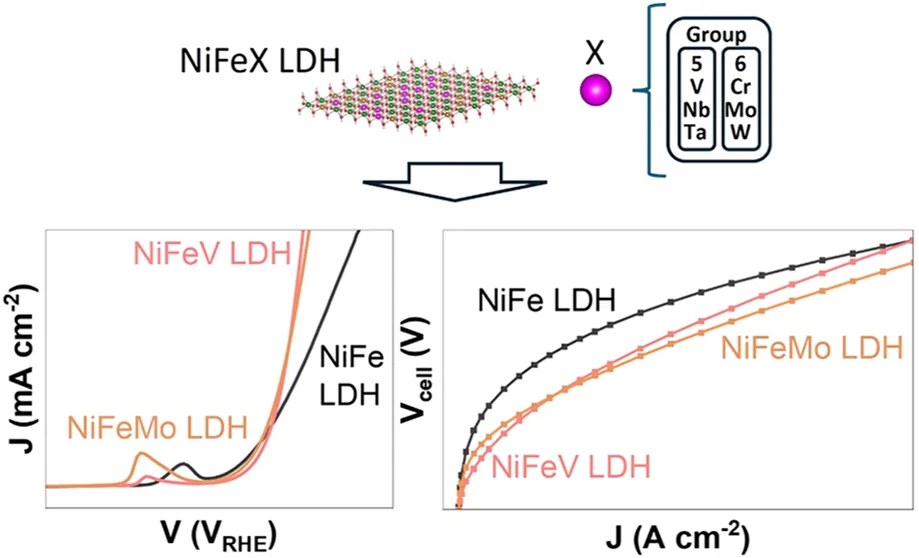

## 1 Introduction

Energy demand has been increasing rapidly for decades, driven by expanding economic sectors such as power, transportation, and industry. Conventionally, such energy demand increments have been realized through fossil primary energy carriers. However, fossil fuels are not abundant, and energy generation from fossil fuels leads to increasing levels of pollutants, such as SO_*x*_ and NO_x,_ contributing to acid rain, fine particulates, and CO_2_, which results in global warming due to the greenhouse effect. Therefore, there is an urgent need to develop clean and sustainable energy sources to replace fossil fuels.^1^

Hydrogen ranks prominently as one of the key alternatives to replace fossil fuels in the power, industry, and transport sectors. Unfortunately, it is difficult to obtain hydrogen directly from nature, and various alternative hydrogen production methods have been investigated. Traditionally, hydrogen is produced mainly by steam methane reforming and coal gasification.^2,3^ However, these processes require large amounts of energy and generate large quantities of pollutants. Thus, water-splitting driven by electricity from renewable resources has been suggested as an alternative, clean, and sustainable method.

Water electrolysis is performed using electrolyzers, where the hydrogen evolution reaction (HER) occurs at the cathode and the oxygen evolution reaction (OER) occurs at the anode. In particular, a sophisticated and detailed understanding of the OER is necessary because it plays an important role in improving the efficiency of water electrolysis due to its sluggish kinetics (four-electron catalytic pathway). Different types of water electrolyzers have been developed and are the subject of current research and development. Among them, low-temperature membrane-based ones, proton exchange membrane water electrolyzers (PEMWEs) and anion exchange membrane water electrolyzers (AEMWEs) are attracting attention. To improve the kinetic efficiency of the OER at the anode in PEMWEs, noble metal-based oxides such as RuO_2_ and IrO_2_ have been reported, with the latter considered the benchmark catalyst because of its chemical stability and superior activity in the acidic environment that characterizes these devices.^4,5^ However, these catalysts are expensive and scarcely available. As an alternative, AEMWEs have emerged as viable alternatives. Owing to the nonacidic environment caused by different ionomers than those used in PEMWEs, non-noble metal-based electrochemical catalysts consisting of 3d-transition metals, such as Ni- or Co-based metal oxides/hydroxides, show promising stabilities as anodes for the OER in alkaline conditions and have been extensively studied. Among Ni-based metal oxides/hydroxides, NiFe layered double hydroxide (NiFe LDH) exhibits the highest OER activity in alkaline conditions.^6^ However, its substantial OER overpotential remains a challenge. Thus, many researchers have attempted to understand the origin of the OER activity at the atomic level by combining experimental results and computational simulations, such as density functional theory (DFT) and Ab-initio molecular dynamic (AIMD) calculations. However, the OER mechanism and active sites of NiFe LDH are still under debate, as various hypotheses have been formulated. Among all, Dionigi and Zeng et al. recently suggested that μ_2_-hydroxo (bridge oxygen Ni–O–Fe) at the edge of the layer of the γ-phase (active phase) is the catalytically active site.^7^ Furthermore, the authors proposed that the presence of Fe at μ_2_-hydroxo has a positive synergistic effect by stabilizing the OER intermediate compared to single-metal bridge oxygens such as Ni–O–Ni and Fe–O–Fe. The potential-determining step (PDS) for NiFe LDH was predicted to be the deprotonation of the OH* intermediate to the O* intermediate, which is significantly facilitated by the presence of Fe with respect to Ni–O–Ni, but remains challenging. Interestingly, they also suggested that for NiMn LDH, even if it is not as active for OER in comparison to NiFe LDH, the Ni site at the subsurface, but close to the catalytically active μ_2_-hydroxo, plays a role in stabilizing OER intermediates.^8,9^ This suggests that a third metal, in addition to the two involved in the active dual-atom μ_2_-hydroxo site, can influence the catalytic activity by an electronic effect.

This concept is highly appealing since it can be combined with other explored strategies that have been shown to enhance the OER activity of (undoped) NiFe LDH, such as introducing modification of crystallinity and morphology^10–12^ as well as immobilization of catalysts on substrates of different porosity and nature.^13–15^ Recently, pioneering works on ternary and multimetallic NiFe-based LDH catalysts incorporated with nonprecious metals, such as lanthanides and high-valence metals from d-block groups 5 and 6, have reported improved OER activity in comparison to the binary NiFe LDH. For instance, Ce-doped NiFe LDH on carbon nanotube achieved a low overpotential (227 mV in 1.0 M KOH) due to mesoporosity and Ce-induced electronic modulation.^16^ Similarly, Cr incorporation has been shown to enhance OER activity,^17^ while Bo et al. attributed improvements to the oxidation of Cr^3+^ to soluble Cr^6+^ (CrO_4_^2–^), generating lattice defects and new active sites as supported by DFT.^18,19^ Jiang et al. demonstrated that Fe/V codoped Ni oxyhydroxide directly deposited on carbon fiber improved activity through V–O bond contraction.^20^ Moreover, Zhang et al. reported superior performance (∼201 mV) for NiFeMo oxyhydroxide on carbon paper, with simulations confirming a key role of Fe^2+^ in enhancing OER activity.^21^

Most of the studies on ternary NiFeX oxyhydroxides/LDHs have focused on the optimization and understanding of one specific choice of metal X or one single metal atomic ratio, such as Ni_1_Fe_1_X_1_. This hampered a wider, more comprehensive, and synoptic understanding of the catalytic activity mechanisms for distinct choices of X. On the other hand, high-throughput approaches, with their great advantage of screening a wide variety of metal compositions and ratios concomitantly, were challenged by limited control of the detailed crystal structure and atomic arrangement of each NiFeX LDH library. In the absence of other hypotheses, “intrinsic” catalytic activity enhancements have most frequently been cited as the origin of the superior performance of some of the ternary catalysts. However, alternative enhancement mechanisms, such as the increase in the electrochemically active surface area (ECSA), by leaching of the X metal atoms, could equally account for the observed catalytic benefits. This knowledge gap will be addressed here.

In this study, we analyze and elucidate metal-specific mechanisms of electrocatalytic OER rate enhancements of a comprehensive set of ternary NiFe-based LDH incorporated with high-valence transition metals across groups 5 and 6, such as V, Nb, Ta, Cr, Mo, and W. The choice of high-valent metals is dictated by their expected stabilization of O* in respect to OH* species, which, as discussed above, should improve the activity of NiFe LDH, by decreasing the overpotential of the OER PDS (OH* → O*). Based on our previous calculations, a third metal site does not need to be at the surface to influence the active sites, which we hypothesize will still be Ni–O–Fe but could occupy a subsurface site.^8,9^ In contrast to previous studies, we prepared ternary NiFe-based LDHs using solvothermal synthesis methods that allowed us to obtain highly crystallized LDH, providing well-defined structures for comparison with theoretical simulations. Furthermore, we investigated three different metal ratios. To accomplish the desired metal atom arrangement with neighboring μ_2_-hydroxo bridges (Figure 1a), we controlled the metal composition by varying the Ni-to-Fe ratio of Ni to Fe while keeping the Fe-to-X ratio of Fe to X constant. The rationale behind the choice of metal ratios is that (1) the activity of NiFe LDHs has been shown to have a maximum between 10 and 30 Fe at %, and (2) we hypothesize one X per Ni–O–Fe site to maximize the impact on the active site, that is, there should be at least one X per Ni–O–Fe, but not too many to block the active sites. Solvothermal synthesis via a microwave autoclave with an autosampler was selected as the synthesis method because it is much faster than conventional autoclave synthesis, which is a great advantage for screening several catalysts. Thus, 18 candidates were screened and compared with three reference NiFe LDHs for a total of 21 catalysts. Their electrochemical OER performance was measured using a three-electrode cell system in 0.1 M KOH. XPS measurements were conducted to validate the presence of highly oxidized X species and to observe their influence on the local electron density of the Ni and Fe species. To evaluate the surface-normalized intrinsic activity of each catalyst, we conducted electrochemical impedance spectroscopy (EIS) according to a well-established measurement protocol for Ni-based oxy­(hydroxide) and compared the calculated ECSAs with those obtained through the integration of the oxidation peak of Ni during anodic sweeping.^22–24^ These electrochemical characterizations were further supported by studying the different activation behaviors of the catalysts using in situ HERFD-XAS combined with DFT calculations to provide insights into their electronic structure and validate the hypothesis of intrinsic enhanced OER activity due to an electronic effect, or disprove it in favor of alternative effects, that is, enhanced ECSA

**Figure 1:**
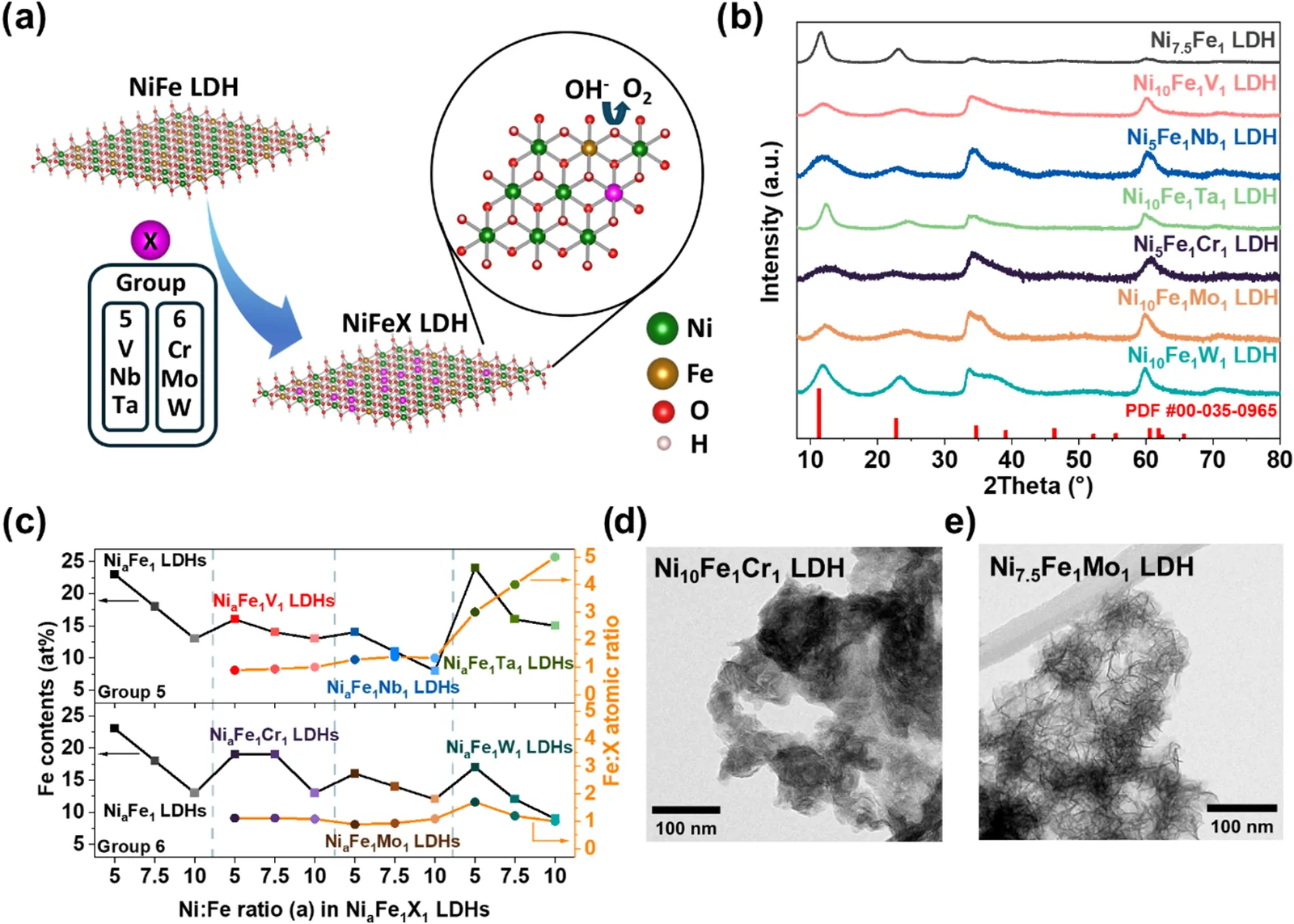
Physico-Chemical characterization of NiFeX LDH. (a) Schematic representation of the strategy used in this study to develop ternary Ni_a_Fe_1_X_1_ LDH and the targeted atom arrangement at the active site. (b) Normalized XRD patterns of as-synthesized Ni_a_Fe_1_X_1_ LDH (with a = 5, 7.5, and 10). The reference XRD pattern of hydrotalcite is shown in red. (PDF #00–035–0965). (c) Fe contents (at %) and Fe:X atomic ratio obtained by ICP-OES analysis. (d, e) TEM images of Ni_10_Fe_1_Cr_1_ LDH and Ni_7.5_Fe_1_Mo_1_ LDH, respectively.

## 2 Experimental Section

### 2.1 Synthesis

The ternary Ni_a_Fe_1_X_1_ LDHs (*a* = 5, 7.5, 10) were synthesized by solvothermal synthesis, varying the Ni:Fe ratio (e.g., 5, 7.5, 10), while keeping the Fe:X ratio constant (e.g., 1:1) (see Supporting Method 1), apart from the following exceptions. Ni_a_Fe_1_Nb_1_ LDHs were synthesized by a two-step synthesis consisting of coprecipitation by employing excess Na_2_CO_3_ and solvothermal synthesis (see Supporting Method 1.3). Ni_a_Fe_1_Mo_1_ LDHs and Ni_a_Fe_1_W_1_ LDHs were synthesized by solvothermal synthesis with modified thermal treatment (see Supporting Method 1.6 and 1.7).

### 2.2 RDE Measurement

RDE electrochemical characterization was performed in a three-compartment glass cell with a rotating disk electrode as the working electrode (RDE, 5 mm in diameter of glassy carbon, Pine Instrument) and a potentiostat (SP-200, Biologic) at room temperature. A Pt-mesh was used as the counter electrode, and a reversible hydrogen electrode (RHE, Gaskatel) was used as the reference electrode, respectively. The electrolytes were prepared with KOH pellets (semiconductor grade, 99.99% metal trace, Sigma-Aldrich) and Milli-Q water. The catalyst was deposited on a glassy carbon electrode by dropcasting using the ink consisting of isopropanol/water solution with Nafion (Perfluorinated resin solution containing Nafion 1100W, 5 wt %, Sigma-Aldrich). The total catalyst loading was 0.1 mg/cm^2^. The detailed ink preparation is available in Supporting Method 3.3. The detailed RDE measurement protocol is provided in Supporting Method 3.4.

### 2.3 AEMWE Single-Cell Measurement

AEMWE performance was evaluated in a 25 cm^2^ single-cell electrolyzer (AEM-Direkt consortium cell) operated with recirculating 1.0 M KOH electrolyte at 70 °C. The preparation of the electrodes followed a protocol developed by Galkina et al.^25^ Electrolyte was supplied separately to the anode and cathode compartments at a flow rate of 100 mL/min. Membrane–electrode assemblies (MEAs) were conditioned and tested following the *EU harmonized polarization curve test method for low-temperature water electrolysis*.^26^ Polarization curves were recorded under steady-state galvanostatic operation, and galvanostatic electrochemical impedance spectroscopy (GEIS) measurements were conducted at 1.0 A/cm^2^ over a frequency range of 300 kHz to 300 mHz. Additional details regarding cell assembly, conditioning procedures, electrode preparation, and electrochemical testing protocols are provided in the Supporting Method 4.

### 2.4 In Situ HERFD-XAS Measurement

In situ HERFD-XAS measurements were performed at ID26 at the European Synchrotron Radiation Facility (ESRF, Grenoble in France). The measurement was performed in a homemade flow cell (0.1 M KOH, 25 mL/min) made of PTFE with a glassy carbon rod as the working electrode. Pt-mesh and RHE were used as counter- and reference electrodes, respectively. The compartment arrangement is depicted in Supporting Figure 1. The catalyst was deposited on laser-engraved glassy carbon disks (*d* = 5 mm) by dropcasting. The ink was prepared similarly to the RDE measurements. The detailed measurement protocol is available in Supporting Method 5.3.

### 2.5 DFT Calculation Parameters and XAS Simulations

We have performed spin-polarized first-principles calculations with the projector augmented-wave method^27,28^ as implemented in the Vienna Ab initio Simulation Package (VASP).^29^ To gain a more accurate description of strongly correlated LDHs with weak interaction between layers, we employed a Hubbard U term to correct the self-interaction errors in 3d transition metal cations together with a van der Waals functional optPBE^30^ to describe the weak interactions in the layered materials, and intercalated water and ions between layers. The U-values applied to the d-orbitals of Ni, Fe and V are 5.0, 2.5, and 3.0 eV, respectively. An energy cutoff of 400 eV with a 4 × 3 × 1 Monkhorst–Pack k-point grid for the Brillouin-zone integration is employed. All the equilibrium geometries are obtained when the maximum atomic forces become less than 0.01 eV/Å and a total energy convergence of 10^–5^ eV is achieved for the electronic self-consistent field loop.

The X-ray absorption spectroscopy (XAS) simulations are performed to investigate the K-edges of individual Ni atoms in the α and γ phases of NiFe LDH. The Ni K-edge XAS simulations in the equilibrium geometric structure are performed using the supercell core-hole (SCH) method, with one full core hole. All the simulated XANES spectra are simulated by introducing a constant Gaussian broadening of (CH_SIGMA) 0.5 eV, equivalent to the full width at half-maximum (FWHM) of ∼1.2 eV. To facilitate a better comparison, all the absorption coefficients are normalized to its maximum in the whole range. We have performed energy alignment with a known experimental reference i.e., 8335.5 eV for the α-phase in the current work. This choice provides a constant value of 119.1 eV, and accordingly, all the simulated XANES were relatively aligned to this constant value. We considered the OH-saturated edge surface of both α and γ phases to investigate the electronic structure (magnetic moments) and K-edge spectra of all nickel atoms in this work. For this, a (2 × 1) supercell for the α-(γ-)­NiFe LDH surface is adopted, which has lattice constants, *a* = 6.22 (5.67) Å, *b* = 7.76 (7.24) Å, and *c* = 36.89 (35.29) Å. The number of unoccupied orbitals or empty bands considered is double as that of occupied orbitals. To investigate the V dopant incorporation in the γ-NiFe framework, all the structures are fully optimized. For investigating OER on γ-NiFeV LDH surface, we have considered a similar (2 × 1) supercell having lattice constants, *a* = 5.75 Å, *b* = 7.13 Å, and *c* = 35.12 Å.

## 3 Results and Discussion

### 3.1 Synthesis and Ex Situ Physicochemical Characterization

A large set of 18 distinct phase-pure ternary Ni_a_Fe_1_X_1_ LDH materials (X = V, Nb, Ta, Cr, Mo, W) were synthesized at the outset of this study. In addition, as benchmark references, binary Ni_a_Fe_1_ LDHs with varying Ni to Fe ratios (*a* = 5, 7.5, and 10 in particular Ni_5_Fe_1_, Ni_7.5_Fe_1_, and Ni_10_Fe_1_ LDH) were synthesized using solvothermal recipes (see Supporting Methods section 1.1). The XRD patterns and TEM images shown in Supporting Figure 2 indicate that all binary Ni_a_Fe_1_ LDHs featured a pure hydrotalcite crystalline structure without additional metal oxide nanoparticles across all selected Ni-to-Fe ratios *a*. Furthermore, the results of ICP-OES, shown in Supporting Table 1, reveal that the Fe contents of Ni_a_Fe_1_ LDHs of all selected ratios are consistent with the targeted Fe content range (e.g., 10–30 at %), at which the OER activity of NiFe LDHs is known to be maximized.^6^ Subsequently, building on the three distinct ratios *a* (i.e., Ni:Fe), a total of 18 ternary Ni_a_Fe_1_X_1_ LDHs were synthesized, keeping the ratio of Fe to X at 1:1, to maximize the probability of having the desired atom arrangement at the edge of the layer, as depicted in Figure 1a. From here on, we will use the notation Ni_a_Fe_1_X_1_ LDH, in order to identify the as-prepared compositions of ternary LDH catalysts.

The majority of the ternary Ni_a_Fe_1_X_1_ (X = V, Nb, Ta, Cr, Mo, W) LDH electrocatalysts investigated in this study were synthesized by solvothermal synthesis followed by thermal postsynthesis treatments, depending on the nature of metal X (see Supporting Table 2 and detailed Supporting Methods section 1). The exception is Ni_a_Fe_1_Nb_1_ LDH, where a two-step synthesis was used, consisting of a coprecipitation and solvothermal treatment, in order to avoid the formation of unwanted metal complexes, such as NiC_2_O_4_, caused by the specific metal precursor (see Supporting Methods 1.3). A synthetic variant was developed for Ni_a_Fe_1_Mo_1_ LDHs and Ni_a_Fe_1_W_1_ LDHs, where a thermal treatment at 50 °C for 15 min preceded one at 120 °C for 60 min owing to the low solubility of the Mo and W precursors (i.e., Mo­(CO)_6_ and W­(CO)_6_) at room temperature in a mixture of Milli-Q water and DMF (see Supporting Methods 1.6 and 1.7). The XRD patterns of the catalytically most active Ni_a_Fe_1_ LDH and Ni_a_Fe_1_X_1_ LDHs—chosen based on their geometric overpotential at 10 mA/cm^2^ in 0.1 M KOH—are shown in Figure 1b. It is noteworthy that all catalysts precisely showed the desired crystalline hydrotalcite structure, which makes their structure–function comparisons, unlike many earlier studies, meaningful. The presence of two characteristic peaks of the hydrotalcite-like structure at 2θ values of approximately 11.5° (003) and 23.5° (006) is noticeable, and the crystallinity varies depending on the incorporated third metal X and the metal content ratios (see Supporting Figure 3). The XRD patterns of all Ni_a_Fe_1_X_1_ LDHs, shown in Supporting Figure 4, indicate that all Ni_a_Fe_1_X_1_ LDHs were of the targeted hydrotalcite crystalline structure without additional undesired metal oxide nanoparticles. Careful inspection of the XRD patterns indicates that Ni_7.5_Fe_1_Ta_1_ LDH shows a minor structural variation since the peak in the 2θ range of 23.5° (006) is separated into two peaks. However, undesired phases were not identified. Furthermore, the targeted Fe content (at %) and Fe:X atomic ratio of all samples was obtained, except for Ni_a_Fe_1_Ta_1_ LDHs, which contained too much Fe compared to Ta. Therefore, further investigation of the synthesis of NiFe-based LDH containing Ta is necessary. A full overview of the atomic ratios present in the synthesized catalysts is presented in Supporting Table 1.

According to the measured TEM images depicted in Figure 1d,e and Supporting Figure 5, the samples showed similar morphologies consisting of agglomerated flakes. Interestingly, Ni_10_Fe_1_Nb_1_ LDH, Ni_10_Fe_1_Mo_1_ LDH, and Ni_10_Fe_1_W_1_ LDH revealed flower-like open morphologies despite their distinctively different synthetic recipes. By contrast, more compact morphologies were displayed by Ni_7.5_Fe_1_ LDH (Supporting Figure 2b) and Ni_10_Fe_1_Cr_1_ LDH.

X-ray Photoemission Spectroscopy (XPS) was performed on selected catalysts to gain insight into the oxidation states of the incorporated “X” metals (see Supporting Table 3). The XPS results showed that highly oxidized ‘X species’ (oxidation state ≥ + III) were indeed obtained. Most of the catalysts presented X in a prevalent oxidation state that far outweighed the one of any other species present. Only N_7.5_Fe_1_Cr_1_ and Ni_10_Fe_1_W_1_, however, showed a mixture of oxidation states (Cr: III and VI; W: IV and VI). All fitted XPS spectra are presented in Supporting Figure 6.

### 3.2 Electrochemical Characterization

#### 3.2.1 Voltammetry of Binary NiFe LDHs

Prior to correlating the surface redox behavior of ternary Ni_a_Fe_1_X_1_ LDHs to their catalytic OER performance, all binary Ni_a_Fe_1_ LDHs (*a* = 5, 7.5, 10) were electrochemically characterized. A break-in procedure of about 50 voltammetric cycles (CVs) was applied until the shapes of the CV curves remained time-stable. The 50th cycle of each Ni_a_Fe_1_ LDH was contrasted in Supporting Figure 7. The anodic Ni oxidation peaks overlapped with the OER onset currents between 1.47 V_RHE_ and 1.50 V_RHE_. With increasing Fe content, the Ni oxidation peak shifted anodically, while its magnitude decreased.^31,32^ Several researchers have suggested possible reasons for this shift based on the results of DFT calculations and *operando* spectroscopy. Friebel et al. proposed that the presence of Fe leads to a shift in the Ni oxidation peak to an anodic potential owing to a decrease in the affinity of the Ni sites to oxygen.^33^ Chen et al. suggested that Fe^4+^ is detectable in NiFe LDH and its presence destabilizes the Ni^3+^ d-orbitals due to the lower electron donor character of the bridge oxygen. As a result, a higher anodic potential is necessary to oxidize Ni from +2 to +3/+4.^34^ In addition, small shoulders were located at potentials prior to the main Ni oxidation peak for Ni_5_Fe_1_ LDH and Ni_10_Fe_1_ LDH, which might be explained by the inhomogeneity of Ni sites in the bulk compared to the edge of the layer.^35^

#### 3.2.2 Voltammetry of Ternary NiFeX LDHs

The 50th CV cycle of the ternary Ni_a_Fe_1_X_1_ LDHs is presented in Figure 2a,b and Supporting Figure 8. Our measurements show that the potential shift in the Ni oxidation peak is largely controlled by the Fe content (at %), as discussed for binary compounds. This trend is particularly evident in the individual voltammetric profiles of each Ni_1_Fe_1_X_1_ LDH (Supporting Figure 8), which highlight the three metal ratios. A comparison of Ni_a_Fe_1_X_1_ LDHs with different X generally shows the same trend, with some exceptions. In the 50th cyclic voltammetry (CV) cycle of the most active Ni_a_Fe_1_X_1_ layered double hydroxide (LDH) for group 5, as depicted in Figure 2a, the Ni oxidation peak of Ni_7.5_Fe_1_ LDH (Fe 18 at %) was observed at a more anodic potential than that of Ni_10_Fe_1_Ta_1_ LDH (Fe 15 at %), which itself was more anodic than that of Ni_10_Fe_1_V_1_ LDH (Fe 13 at %), consistent with the expected trend. Notably, the Ni oxidation peak of Ni_5_Fe_1_Nb_1_ LDH (Fe 14 at %) was nearly at the same potential as that of Ni_7.5_Fe_1_ LDH (Fe 18 at %), despite the lower Fe atomic percentage. Similarly, Figure 2b for group 6 illustrates that the Ni oxidation peaks of Ni_7.5_Fe_1_ LDH (Fe 18 at %) and Ni_5_Fe_1_Cr_1_ LDH (Fe 19 at %), which have comparable Fe atomic percentages, appear within the same potential window and are more anodic than those of Ni_10_Fe_1_Mo_1_ LDH (Fe 12 at %) and Ni_10_Fe_1_W_1_ LDH (Fe 9 at %). However, the Ni oxidation peak of Ni_10_Fe_1_Mo_1_ LDH shifted in a more cathodic direction than that of Ni_10_Fe_1_W_1_ LDH, despite the higher Fe atomic percentage. These variations in the trend may suggest partial and varying degrees of Fe incorporation within the LDH structure, potentially due to the formation of small FeOOH clusters, as reported in the literature.^36,37^ Alternatively, it may be hypothesized that the incorporation of the third metal, X, alters the electronic structure of the Ni site. Remarkably, Figure 2b shows that the Ni oxidation peaks of Ni_10_Fe_1_Mo_1_ LDH and Ni_10_Fe_1_W_1_ LDH, which were synthesized with Mo­(CO)_6_ and W­(CO)_6_ using a modified thermal treatment (see Supporting Methods sections 1.6 and 1.7), are much larger than those of other ternary Ni_a_Fe_1_X_1_ LDHs. We discuss this in more detail later in the paper.

**Figure 2:**
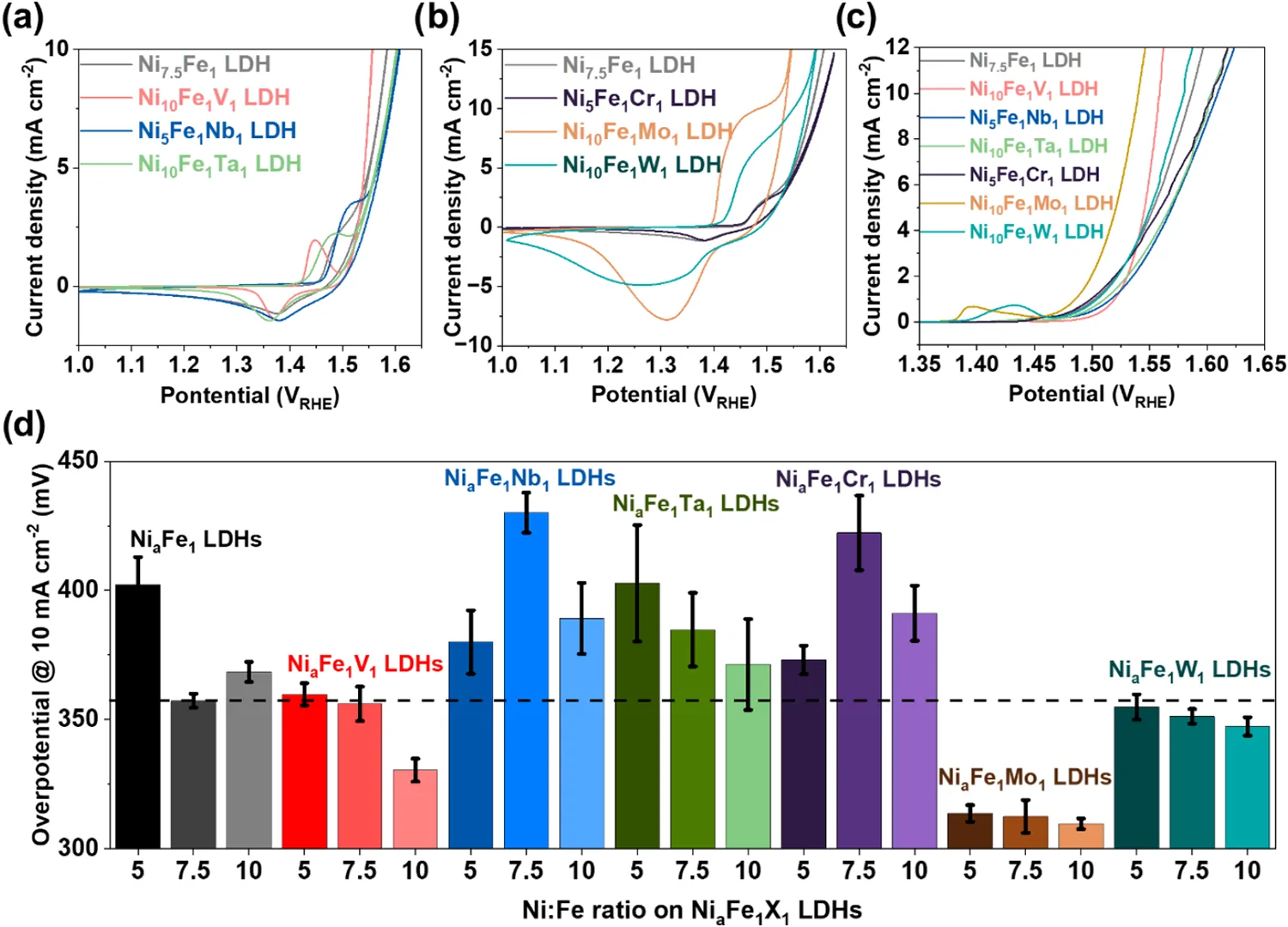
Electrochemical characterization and geometric activity. (a, b) 50th cycle of cyclic voltammetric curves (CVs) of the most active catalysts for groups 5 and 6, respectively, at a scan rate of 50 mV/s in 0.1 M KOH by rotating disk electrode (RDE) (1600 rpm). (c) Corresponding linear sweep voltammetric curves (LSVs) at a scan rate of 1 mV/s in 0.1 M KOH by RDE (1600 rpm). Catalyst loading on the GC electrodes: 0.1 mg/cm^2^. (d) Comparison of geometric overpotential at 10 mA/cm^2^ for groups 5 and 6. The horizontal dashed line in (d) demarcates the geometric overpotential at 10 mA/cm^2^ for Ni_7.5_Fe_1_ LDH. RDE measurements were performed at least three times to obtain a statistical average, and the corresponding standard deviation is illustrated by the error bars in (d).

#### 3.2.3 Catalytic OER Activity Trends

Linear sweep voltammetry was performed to investigate the basic geometric OER activity of ternary LDH catalysts. Figure 2c evidence that the Ni_10_Fe_1_Mo_1_ LDH leads the OER activity trend in terms of kinetic overpotential at a geometric current density of 10 mA/cm^2^. Ni_10_Fe_1_Mo_1_ LDH revealed an unprecedented kinetic enhancement of ∼47 mV compared to the most active binary Ni_7.5_Fe_1_ LDH. The next most active ternary LDH was Ni_10_Fe_1_V_1_ LDH displaying an overpotential shift of ∼27 mV thanks to its lower Tafel slope (∼35.1 mV/dec) compared to other catalysts (see Supporting Figure 9). Finally, the Ni_10_Fe_1_W_1_ LDH showed a more moderate enhancement (∼10 mV) compared to that of the Ni_7.5_Fe_1_ LDH. As shown in Figure 2d, the OER activities of other catalysts, such as Ni_a_Fe_1_Nb_1_ LDHs, Ni_a_Fe_1_Ta_1_ LDHs, and Ni_a_Fe_1_Cr_1_ LDHs, were inferior to those of our benchmark Ni_7.5_Fe_1_ LDH. Our data suggest the following geometric reactivity trend: Ni_10_Fe_1_Mo_1_ LDH > Ni_10_Fe_1_V_1_ LDH > Ni_10_Fe_1_W_1_ LDH > Ni_7.5_Fe_1_ LDH > Ni_10_Fe_1_Ta_1_ LDH > Ni_5_Fe_1_Cr_1_ LDH > Ni_5_Fe_1_Nb_1_ LDH, which is largely consistent with the geometric OER activity trends reported in the literature and measured in 1.0 M KOH.^17,20,21^ Furthermore, we note that our reference NiFe LDH catalyst, showing overpotentials close to 350 mV at 10 mA/cm^2^, represents the current state-of-the-art catalytic OER activity reported in 0.1 M KOH; this makes it a meaningful and challenging benchmark for any ternary compounds^8,9^

#### 3.2.4 Real ECSA and Intrinsic OER Activity Trend

The turnover frequency (TOF) is the metric of choice in catalysis and electrocatalysis to estimate and compare the intrinsic catalytic activity per catalytically active center at the surface. However, accurate intrinsic catalytic activities were rarely reported for Ni-based (oxy)­hydroxides, such as NiFe LDH, because of the complexity of calculating reliable surface concentrations of active sites of irregular nanoplate morphology combined with the uncertainty in the determination of the nature of active sites. Furthermore, other established procedures to estimate the intrinsic activity based on the ECSA calculated by normalizing the double-layer capacitance (*C*_DL_) to the specific capacitance (*C*_s_)^38^ are also unsuitable for NiFe LDH owing to its nonconductive character at nonfaradaic potentials.^24,39^ Recently, we have reported and verified for powdered NiFe LDH catalysts an adsorbate capacitance (*C*_a_)-based EIS technique- to extract accurate real ECSA values by using the equivalent electric circuit shown in Supporting Figure 10.^21,23,24^ Here, we adopted our technique and succeeded in extracting adsorbate capacitance (*C*_a_) and real ECSA values under OER conditions for our present set of NiFeX LDH catalysts. Our results constitute the largest ever reported set of intrinsic ECSA and activity values for NiFe LDH-based catalysts.

Figure 3a shows the intrinsic ECSA values (black line) and Ni oxidation charges (orange line) of all LDH OER catalysts investigated in this study. We note that the ECSA trends are closely correlating with those in the Ni oxidation charge. As expected, Ni_a_Fe_1_Nb_1_, Ni_a_Fe_1_Mo_1,_ and Ni_a_Fe_1_W_1_ LDHs with their flower-like open morphologies showed some of the largest ECSA values, whereas Ni_7.5_Fe_1_Cr_1_ LDH, with its highly agglomerated morphology, showed the lowest ones. Finally, Figure 3b,c reports the intrinsic OER activity trends. The Ni_7.5_Fe_1_Cr_1_ LDH catalyst displayed the lowest overall kinetic overpotential (i.e., largest intrinsic catalytic activity) at 0.5 mA/cm_ECSA_^2^ with significant kinetic benefits compared to Ni_10_Fe_1_ LDH, while the other catalysts have no enhancement in intrinsic activity. The flower-like open morphology with their large real ECSA values for Ni_5_Fe_1_Nb_1_ LDH and Ni_10_Fe_1_Nb_1_ LDH put them lower in the intrinsic activity order, similar to Ni_a_Fe_1_Mo_1_ LDHs and Ni_a_Fe_1_W_1_ LDHs. We hypothesize that the origin of their large ECSA lies in their specific synthesis involving an additional preheating step and the effect of CO ligands during the synthesis. Supporting Discussion 1 and Figures 11, 12 detail the experimental verification of our hypothesis of the correlation between synthesis and resulting ECSA, and show which parameter has the largest impact. We conclude that the flower-like open morphology, which is beneficial for the number of active sites and thus the ECSA characteristics of Ni_a_Fe_1_Mo_1_ LDHs and Ni_a_Fe_1_W_1_ LDHs, can be attributed to the presence of CO ligands during synthesis.

**Figure 3:**
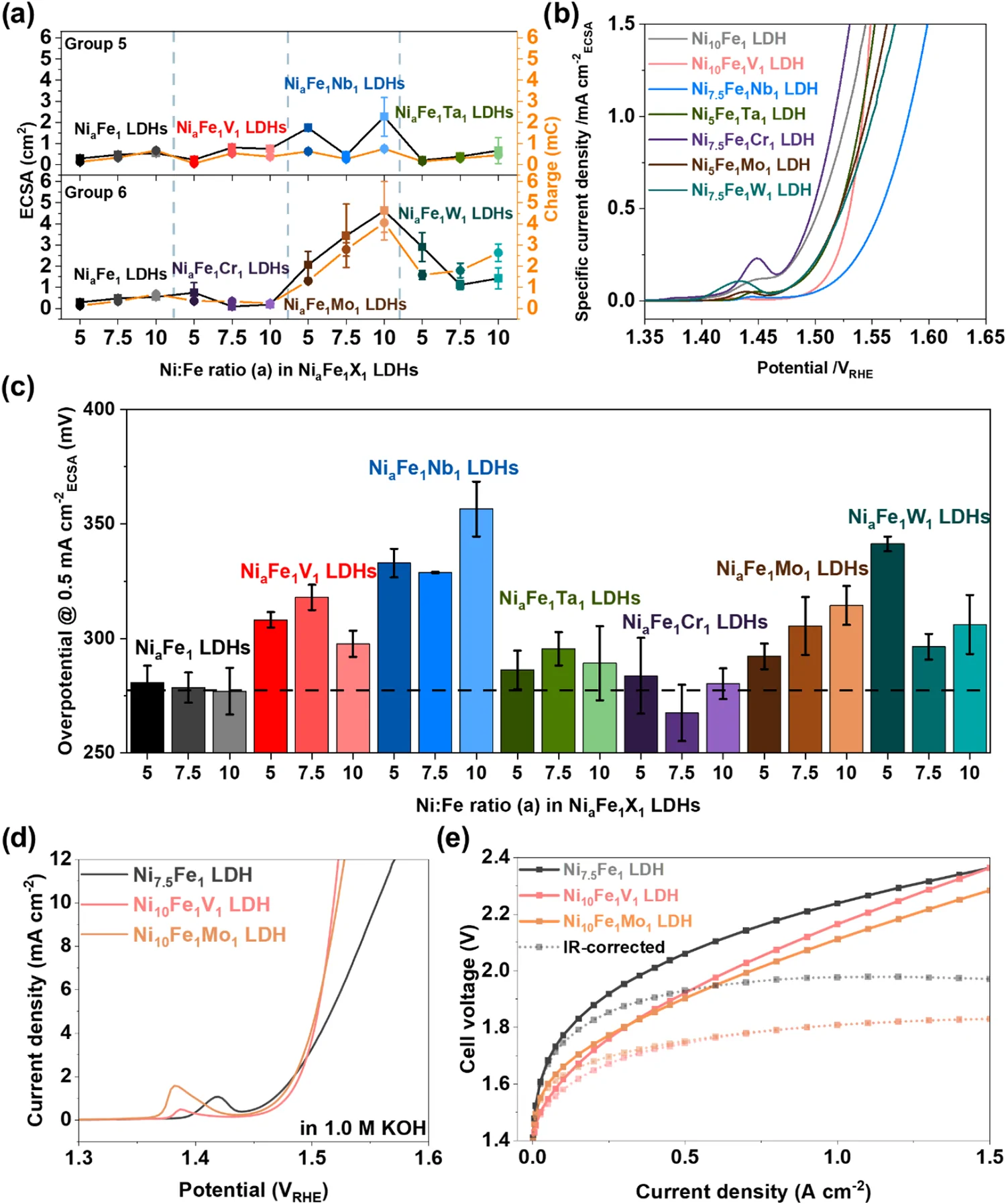
Surface-normalized intrinsic activity and MEA measurements. (a) ECSA values calculated by fitting Nyquist plots obtained from electrochemical impedance spectroscopy (EIS) at 1.60 V_RHE_ using an equivalent electric circuit model (see Supporting Figure 10) and the Ni oxidation charge obtained from the 50th cycle for groups 5 and 6. (b) Linear sweep voltammetric curves (LSVs) of each of the most intrinsically active catalysts showing specific current densities obtained by normalizing the current to the ECSA values at a scan rate of 1 mV/s in 0.1 M KOH by RDE (1600 rpm). (c) Comparison of intrinsic overpotential at 0.5 mA/cm_ECSA_^2^ for groups 5 and 6. The horizontal dashed line in (c) demarcates the intrinsic overpotential at 0.5 mA/cm_ECSA_^2^ of Ni_10_Fe_1_ LDH. Error bars represent the standard deviation. (d) Linear sweep voltammetric curves (LSVs) of selected catalysts (i.e., Ni_7.5_Fe_1_ LDH, Ni_10_Fe_1_V_1_ LDH, and Ni_10_Fe_1_Mo_1_ LDH) at a scan rate of 1 mV/s in 1.0 M KOH by RDE (1600 rpm). (e) AEMWE single-cell measurements employing selected catalysts (i.e., Ni_7.5_Fe_1_ LDH, Ni_10_Fe_1_V_1_ LDH, and Ni_10_Fe_1_Mo_1_ LDH) CCS-based anodes. Polarization curves were recorded employing a AEMWE single-cell (active area of 25 cm^2^) at 70 °C using 1.0 M KOH and a DURAION anion-exchange membrane (Evonik, 70 μm thickness). The anode catalysts (∼1.1 mg_**cat**_/cm^2^) and cathode catalyst Pt/C (∼0.2 mg_**Pt**_/cm^2^) were directly spray-coated onto a stainless-steel porous transport layer (PTL, 800 μm) supplied by Siemens Energy. Solid lines represent the measured cell voltages, while the dotted lines correspond to *IR-corrected* cell voltage based on the high-frequency resistance obtained from galvanostatic electrochemical impedance spectroscopy (GEIS) (see Supporting Figure 13). Further detailed information is available in Supporting Method 4.

While RDE measurements provide valuable insights into the intrinsic activity of the catalysts under well-defined and controlled conditions, they do not fully capture the complexities encountered in operating electrolyzers, where additional factors (e.g., mass transport, catalyst layer architecture, membrane-electrode interactions) are present.^40,41^ We therefore conducted single cell measurements to determine whether the activity trends observed in RDE experiments translate to improved activity in devices.

#### 3.2.5 MEA Measurements

Before conducting AEMWE single-cell measurements, the selected catalysts—Ni_7.5_Fe_1_ LDH as a benchmark catalyst, Ni_10_Fe_1_V_1_ LDH owing to its lowest Tafel slope and high geometric activity superior to Ni_7.5_Fe_1_ LDH, and Ni_10_Fe_1_Mo_1_ LDH owing to its highest geometric activity and highest ECSA—were evaluated by RDE measurements in 1.0 M KOH to verify whether the activity trend observed in lower electrolyte concentration (0.1 M KOH) is maintained under the electrolyte conditions (1.0 M KOH) used for single-cell tests. As shown in Figure 3d, all catalysts exhibited the expected increase in OER activity at higher pH, even on the RHE scale (supernernstian behavior), consistent with observations for NiFe LDH-based catalysts.^42^ The measurements further confirmed that the geometric activity trend among the investigated catalysts was preserved in 1.0 M KOH. Based on these results, AEMWE single-cell measurements were performed using the three selected catalysts, as shown in Figure 3 *e*, with 1.0 M KOH feeds (symmetric flow configuration). Polarization curves were recorded according to the *EU harmonized protocol.* ^26^ (see Supporting Method 4).

The non-IR-corrected polarization curves reveal that both cells with Ni_10_Fe_1_V_1_ and Ni_10_Fe_1_Mo_1_ LDHs, incorporating high-valent transition metals (V and Mo), outperform the reference cell with Ni_7.5_Fe_1_ LDH, in the entire current densities range up to ∼1.4–1.5 A/cm^2^. Particularly, at current densities below 0.3 A/cm^2^ the Ni_10_Fe_1_V_1_ LDH cell exhibits slightly higher performance than the Ni_10_Fe_1_Mo_1_ LDH cell. However, with increasing current density, the relative performance of the two catalysts reverses, with the Ni_10_Fe_1_Mo_1_ LDH cell outperforming at higher current densities. Notably, the same trend is preserved in the IR-corrected polarization curves, where the Ni_10_Fe_1_V_1_ LDH cell maintains a marginal advantage below 0.7 A/cm^2^, while both NiFeX LDH cells display comparable activities at higher current densities. These observations are consistent with the RDE measurements performed in 1.0 M KOH.

Furthermore, Ni_10_Fe_1_V_1_ and Ni_10_Fe_1_Mo_1_ LDH cells exhibit a nearly linear increase in cell potential with increasing current density, suggesting that the overall cell performance is predominantly governed by ohmic losses. In contrast, such behavior is not observed for the reference catalyst, Ni_7.5_Fe_1_ LDH. This deviation may originate from partial detachment of the catalyst layer from the PTL during operation. Indeed, catalyst-deficient regions were clearly visible on the PTL after testing, while residual catalyst deposits were observed on the membrane surface. Catalyst stability will be discussed in Section 3.4.

### 3.3 Molecular X-ray Based Characterization of LDH Catalysts

#### 3.3.1 Electronic Effects in As-Prepared NiFeX LDHs

We conducted XPS on a selected set of as-prepared pre-electrocatalysts in order to probe the chemical states of its constituent elements prior to catalytic testing. We refer to these materials as precatalysts because their catalytically active γ-phase generally forms under extended anodic polarization. This set included exactly one Ni_a_Fe_1_X_1_ LDHs ternary sample out of each group of three (*a* = 5, 7.5, 10), which turned out to be the catalytically most active during RDE testing.

Supporting Figure 15 displays the Ni-2p and Fe-2p photoemission core level spectra of the as-prepared precatalysts. The third metal “X” has no apparent influence on the chemical states of Ni or Fe ions in the catalysts. However, based on the fitted XPS spectra (Supporting Figure 6 and Supporting Table 3), the selected early d-block metals “X” can be assumed to be present in a redox state of > + III in the NiFeX lattice. This state should result in a higher electronegativity. We could show in a previous work that for the binary NiFe LDH, the catalytically active surface site is the surface μ_2_-oxo-bridge linking a Ni- and a Fe-cation at the edge of a 2D sheet (the so-called dual-metal edge site).^7^ As a result of this, we expected that a high-valent metal ion “X” in close proximity to the dual-atom site should draw electron density away from Ni/Fe centers, resulting in a core level shift toward higher binding energies. However, such a shift was absent, which made us conclude that direct electronic effects of X on the catalytic surface site are negligible in the as-prepared catalyst.

#### 3.3.2 In Situ Electronic Effects in Activating NiFeX LDH Catalysts

To probe the influence of the “X” elements on the electronic structure of the activated NiFe LDH host material during OER, we conducted in situ high-energy resolution fluorescence detected X-ray absorption spectroscopy (HERFD-XAS) experiments using the beamline ID26 at the European Synchrotron Radiation Facility (ESRF). HERFD-XAS was chosen because it yields high energy resolution, which allows us to properly investigate the changes in the pre-edge energy region. The so-called pre-edge region is particularly interesting for catalysis, as pre-edge peaks are associated with the dipole forbidden s → d transitions, thus providing valuable information about the density of states of the d-band with implications on structure and adsorption. In the case of NiFe LDH and NiFeX LDHs, the d-band should be only partially unfilled, and changes in the pre-edge range could therefore be indicative of changes in the adsorption behavior of reaction intermediates.^43^

For the *operando* X-ray absorption near-edge spectroscopy (XANES) experiments, we implemented an electrochemical cell with a Mylar window (see Supporting Figure 1) in fluorescence geometry. The catalyst was immobilized on glassy carbon rods and subjected to a range of electrode potentials spanning the pre-OER and OER regions (1.1 V, 1.4 V, 1.6 V, 1.1 V, 1.9 V, here all potential values are provided with respect to the RHE) in 0.1 M KOH electrolyte. We concentrated our effort mainly on the Ni–K-edge XAS, while the Fe–K-edge XAS spectra (Supporting Figure 16) and discussion (Supporting Discussion 2) can be found in the SI.

Before we move to the ternary catalysts, we provide a detailed spectral analysis of the reference material. The resulting Ni K-edge XANES spectrum of the NiFe LDH reference catalyst is shown in Figure 4a, and the inset shows its pre-edge region in more detail. Increasing the electrode potential anodically, the binary NiFe LDH-reference catalyst did not reveal any significant shift in its Ni K-edge energy position, yet displayed a monotonic decline in its white-line intensity, which is not recovered even after returning to 1.1 V. This behavior is typical of limited α to γ conversion, and often observed in thick catalyst layers of unsupported and powdered NiFe LDH in 0.1 M KOH.^44^ In our analysis, we focused on the dipole-forbidden pre-edge peak. A single pre-edge peak was discernible between 1.1 and 1.4 V, which we will refer to as peak 1. Increasing the potential more anodically, a broad pre-edge peak shoulder emerged on the high-energy side of peak 1, which extended and overlapped with the Ni K-edge onset. We assigned the appearance of this feature to the emergence of the structurally and electronically distinct, catalytically active γ-NiFe LDH phases. The shape of the pre-edge region is the result of the overlap of peak 1 with a second peak (henceforth referred to as pre-edge peak 2). This interpretation is in agreement with earlier pre-edge studies investigating NiFeO_*x*_H_*y*_ catalysts, where peak 2 is distinctly observable only at a high α to γ conversion.^33,45^ Thus, the broadness of the emerging pre-edge feature suggests an incomplete α to γ conversion in our NiFe LDH, which is plausible given the thick catalyst layer used here. When the applied electrode potential was returned to 1.1 V after a long 1.5 h hold at 1.6 V, the γ-pre-edge feature failed to immediately fade. This slow γ to α discharge hysteresis can be caused by poorly conductive α-phase domains associated with prolonged trapping of conductive γ-phase domains.^8^ The limited rate of the concomitant ion exchange of intercalated cations and anions during the phase transition adds to a slow discharge. Finally, we cannot exclude that this process is further exacerbated by the less-than-ideal electrical field distribution in the in situ electrochemical cell.

**Figure 4:**
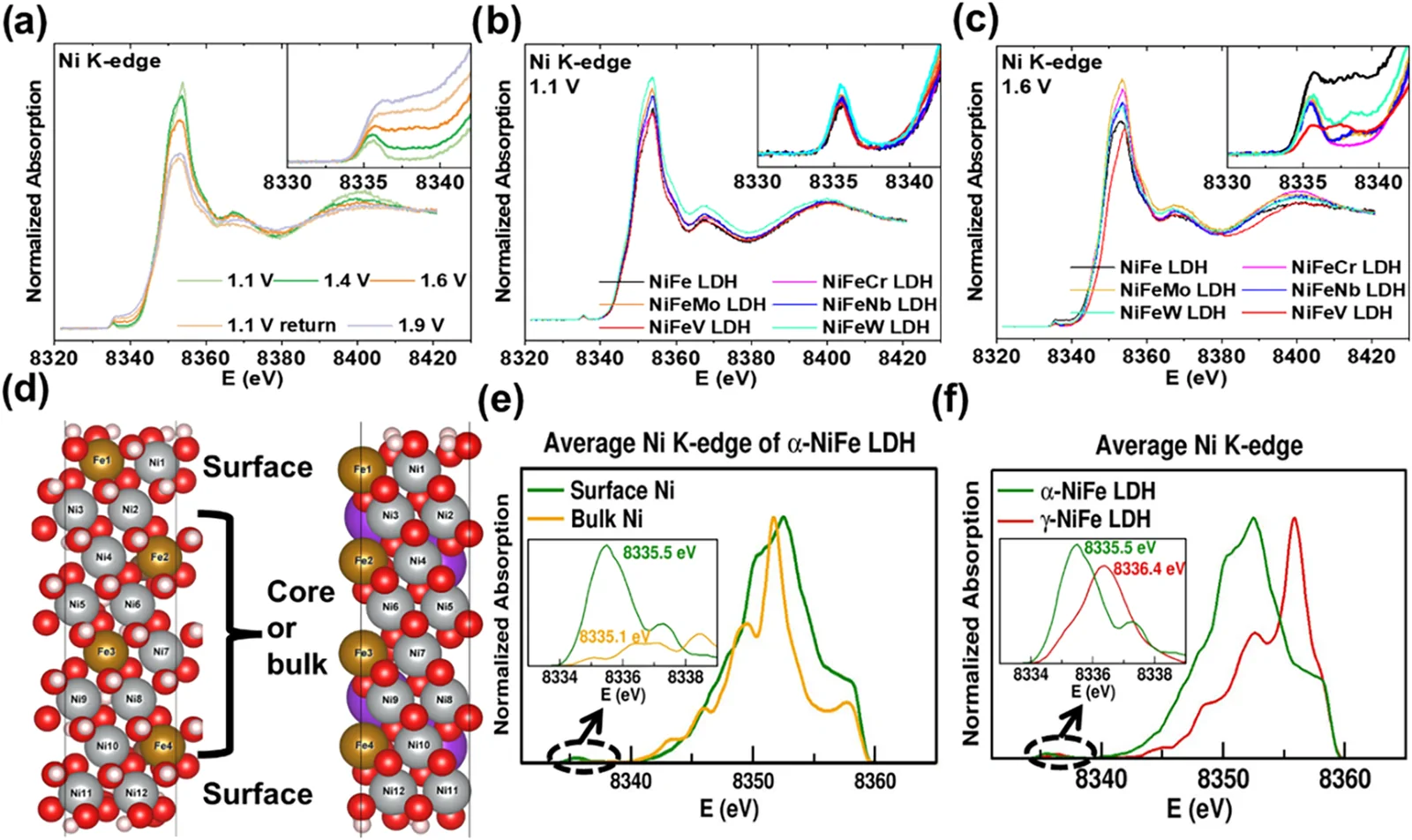
In situ HERFD-XAS and spectral DFT calculations. (a) Experimental HERFD-XAS Ni K-edge spectra of NiFe LDH at various applied potentials. The inset shows a magnified pre-edge region. (b, c) Experimental HERFD-XAS spectra of the Ni K-edge of NiFeX LDH at 1.1 and 1.6 V, respectively. Insets in (b, c) show the zoomed-in pre-edge regions of the spectra. (We note that the data set for NiFeNb overlaps almost completely with that of NiFeMo, making the former hard to see). (d) Front view of the atomic arrangement for the α-NiFe LDH surface saturated by OH, with CO_3_^2–^ ions intercalated into the interlayers (left), and for γ-NiFe LDH surface saturated by OH, with K^+^ cations intercalated (right). Water molecules were intercalated into the interlayers of both phases. The color codes for Ni, Fe, C, O, H, and K atoms are gray, gold, brown, red, pink, and purple, respectively. (e) Predicted Ni K-edge of Ni atoms with 2+ and 3+ oxidation states in the α-phase. (f) Predicted Ni K-edge of Ni atoms in the α- and γ-phases. Here, for the α-phase, only Ni atoms with 3+ oxidation state were considered. All curves were normalized to the maximum value over the entire range.

We now focus on Ternary NiFeX LDHs. Figure 4b,c compares the Ni K-edge XANES spectra of NiFeX LDHs at 1.1 and 1.6 V, respectively, showing the catalysts in the pre-OER regime as well as during OER. At 1.1 V, all NiFeX LDH catalysts showed very similar spectral traces inside the pre-edge region, confirming our conclusion that the addition of metal “X” did not inherently change the state nor geometry of Ni in the pristine catalysts. However, we observed that 1.6 V, the catalysts showed quite distinct spectral behaviors depending on the nature of the incorporated metal “X”.

The most distinct spectra were obtained for NiFeV LDH at 1.6 V. Not only was the XANES spectrum of NiFeV LDH the only one that showed a clear shift in the Ni K-edge position to higher energy, indicating a higher-valent Ni species, but the pre-edge peaks of this catalyst were also distinct in position and width (red symbols in Supporting Figure 17a,b) from those obtained for the NiFe LDH reference material and the other NiFeX LDHs. The pre-edge peak 2, marking the active γ-phase, was blue-shifted by ∼1.5 eV from peak 1, which is smaller compared to other NiFeX LDHs (see Supporting Figure 17a,b). This spectral behavior could reveal energetically lowered empty d-band states, indicating an electronic effect of the incorporated V-species on the NiFe LDH host material. An earlier pre-edge analysis of NiFeO_*x*_H_*y*_ with varying Fe contents reported no dependence of peak 2 on Fe%, which excludes compositional variation as origin of the shifts in peak 2 observed here.^33^ Further DFT calculations concerning the incorporation and effect of V in NiFeV LDH can be found in chapter 3.4.

In contrast to the unique small energy separation of peak 1 and 2 in NiFeV LDH, NiFeMo LDH, NiFeNb LDH, and NiFeW LDH similarly exhibited the formation of the activation pre-edge peak 2 at approximately 2.5 eV above the pristine pre-edge peak 1 (Supporting Figure 17a). For NiFeCr LDH, no emergence of peak 2 was detectable (hence absent in Supporting Figure 17), indicating very low conversion of α to γ, which is consistent with the low activity of this catalyst in RDE.

We emphasize that HERFD-XAS in fluorescence detection geometry averages over the illuminated catalyst layer, so the relative intensity of pre-edge peak 2 reports the fraction of Ni centers that have converted to the γ-phase environment within the probed volume, rather than an intrinsically local or atom-specific electronic signature. Because this converted fraction should scale with the electrochemically accessible surface area of the layer, we cannot fully exclude that the peak 2 intensities observed for the high-ECSA NiFeMo, NiFeNb, and NiFeW LDHs compared to low-ECSA NiFeCr LDH partly reflect a larger active fraction of the sample rather than an intrinsically facilitated phase transition. We want to stress however that the complete absence of peak 2 in NiFeCr LDH suggests that some X species may hinder the formation of the active phase altogether. The NiFeV case is more directly interpretable in regard to electronic effects: here, the diagnostic feature is a shift in the energy position of peak 2 rather than simply its intensity, and NiFeV LDH does not display an enhanced ECSA (Figure 3a).

In conclusion, our analysis of the sensitive pre-edge region of the Ni K-edge appears to suggest that direct electronic effects of most X species on the dual-atom surface sites are not significant and can be excluded as the origin of variations in catalytic OER rate. However, the analysis revealed that the metals X affect the degree to which the various catalysts activate into their γ-NiFeX LDH phase. Furthermore, a spectral feature in γ-NiFeV LDH suggests an electronic effect, which might act as a distinctly different performance modulation mechanism than previously suspected.

#### 3.3.3 DFT Computational Spectral Verification

To verify and better understand our hypothesis as to the shift in the pre-edge features between the inactive α-phase and active γ-phase, we carried out DFT calculations to investigate the electronic structures (magnetic moments, μ_B_) and Ni K-edge absorption spectra of NiFe LDH catalysts. As in our earlier works,^7–9^ we adopted a model of the α-NiFe LDH with the formula Ni_6_Fe_2_CO_3_(OH)_16_·4H_2_O (bulk Ni^2+^ and Fe^3+^) and a model of γ-NiFe LDH with the formula Ni_6_Fe_2_K_2_O_16_·4H_2_O (bulk 4 Ni^4+^, 2 Ni^3+^, Fe^4+^), as shown in Figure 4d. In the α-phase, the surface Ni atoms (Ni1, Ni11, and Ni12) are in an oxidation state of +3, rather than an oxidation state of +2 as in the bulk, because they are partially oxidized by OH adsorption on the under-coordinated top site. The magnetic moments associated with the Ni^2+^ and Ni^3+^ atoms were 1.8 and 1.0 μ_B_, respectively. For the γ-phase, the Ni atoms are in +3 and +4 oxidation states, characterized by magnetic moments of 1.0 and 0.0 μ_B_, respectively.

Figure 4e compares the predicted K-edge of XANES for Ni atoms with +2 and +3 oxidation states in the α-phase. Notable differences were observed in the pre-edge region, i.e., a clear peak is only visible for Ni atoms with +3 oxidation states, at 8335.5 eV. This more intense peak for surface Ni atoms is consistent with the related more intense Ni 4p and 3p states above the Fermi level in the projected density of states (PDOS) (see Supporting Figure 18a), and 1s → 4p/3d transition for Ni^3+^ pre-edge. The notable Ni 4p states are likely due to strong hybridization between the 3d and 4p states of Ni^3+^. For Ni^2+^ there is no p-d mixing; therefore, no dipole-allowed transition exists, and hence, only a very weak quadrupole peak is observed. Consequently, the experimentally observed pre-edge feature in the α-NiFe LDH predominantly arises from partially oxidized surface Ni^3+^ metal centers, in contrast to bulk Ni^2+^. To understand the experimental energy shift in the pre-edge feature we compared the predicted Ni K-edge XANES of Ni^3+^ centers in α-NiFe LDH to the one of Ni centers in γ-NiFe LDH (Figure 4f). A clear, well-defined, yet quite distinct pre-edge peak feature was observed for the α- and γ-phases at 8335.5 and 8336.4 eV, respectively. A pre-edge peak energy shift of 1.0 eV was calculated between the two phases, which is congruent with our experimental findings above. Thus, the positive energy shift in the pre-edge peak position indicated the formation of higher valent metal states under OER conditions. From the PDOS of γ-NiFe LDH (see Supporting Figure 18b), the entire states, especially clearly seen for the 3d and 4p states within the band gap, are shifted down by ∼1 eV. Considering a similar downshift of the 1s level, and the nearly fixed unoccupied 4p and 3d states (i.e., 0.5–1 eV above the Fermi level), the 1s → 3d and 4p transition would lead to a positive energy shift of the pre-edge peak for the catalytically active γ-phase compared to the catalytically inactive α-phase. To summarize, DFT calculations confirmed our assumption that the second pre-edge peak 2 is assigned to the formation of the active γ-phase. As such, the pre-edge peak 2 is a valid measure for studying the conversion of the catalysts to the γ-phase, that is the activation of the catalyst.

### 3.4 DFT Calculations on NiFeV

To gain further insight into the role of V in γ-NiFeV LDH, we performed DFT calculations examining V as a dopant both at the surface and in the subsurface. We first assessed the thermodynamic stability of bulk γ-NiFeV LDH by calculating its free energy of formation based on the reaction of binary reference compounds (see Supporting Discussion 5). The calculated free energy of formation of γ-NiFeV LDH (+0.05 eV/M) indicates slight metastability with respect to the selected reference species, suggesting a thermodynamic driving force for V segregation and dissolution. To examine the possible distribution of Fe and V within the γ-NiFe LDH framework, we considered two representative bulk arrangements with different relative atomic positions of Fe and V (see Supporting Figure 27). The two bulk arrangements are nearly degenerate (Δ*E* = 26 meV/Ni_6_FeVK_2_O_16_·4H_2_O), suggesting that both configurations may coexist and are therefore both relevant for constructing surface models. In both bulk configurations, V adopts a +5 oxidation state, while Fe is +4 in the most stable configuration and +5 in the slightly less stable one. The average Ni oxidation state is +3.3 for the most stable and +3.5 for the slightly less stable bulk configuration. This highlights that the incorporation of V induces electronic structure modulation already in the bulk.

To disentangle the catalytic role of surface V from the electronic modulation effects of subsurface V, we constructed two surface models based on the nearly degenerate bulk arrangements: an Fe-terminated surface (Figure 5a, V located in the third layer) and a V-terminated surface (Figure 5b, Fe located in the third layer). The calculated surface energies of the two terminations differ by only 18 meV, confirming that both surface terminations are energetically favorable and may coexist under experimental conditions. For each surface, we first constructed surface phase diagrams to identify steady-state surface configurations under OER conditions, before calculating the OER reaction free energy profiles following a Mars van Krevelen-type mechanism, in which the reaction begins from the deprotonation of surface OH species at bridge sites.

**Figure 5:**
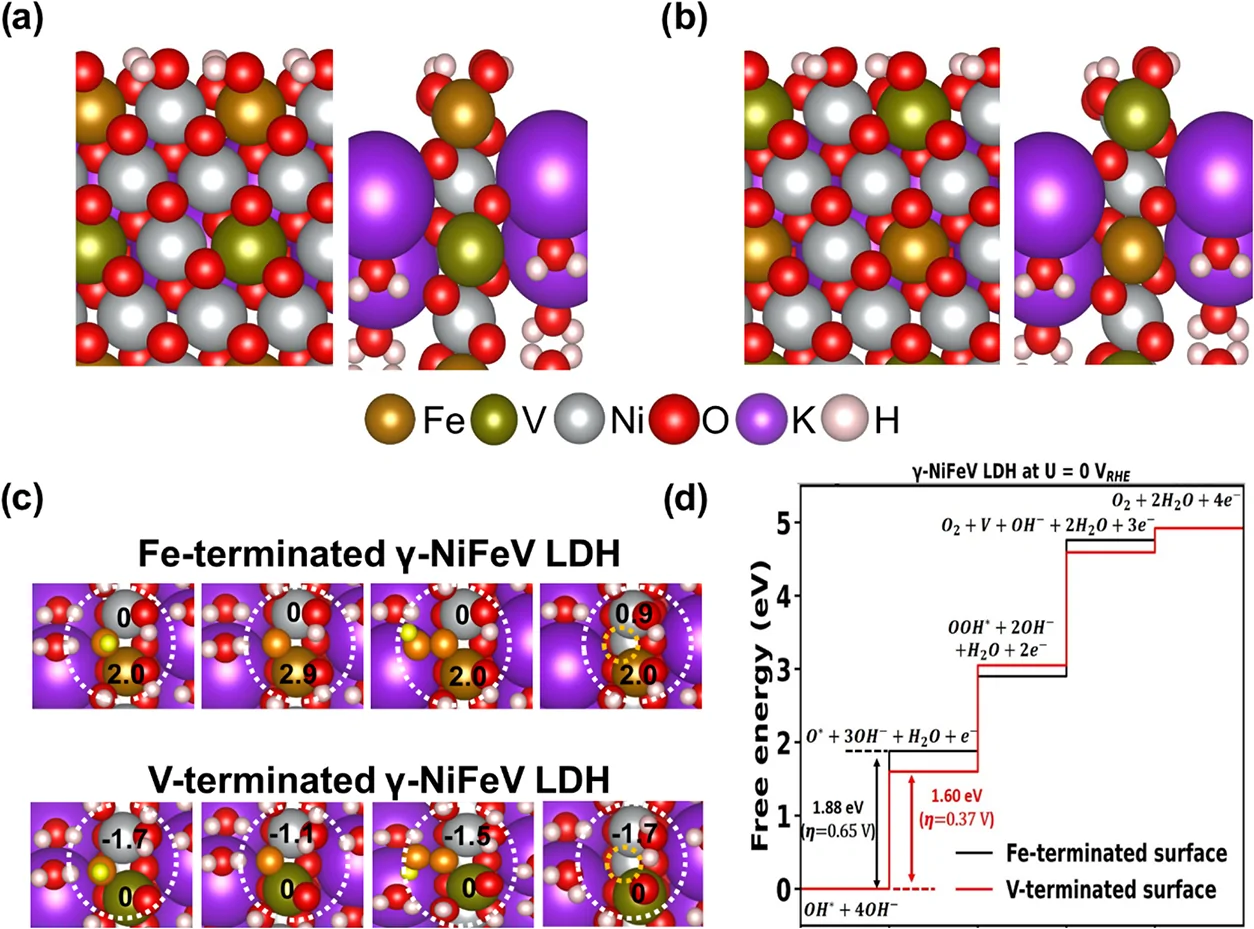
NiFeV LDH surfaces and DFT calculations. (a) Fe-terminated and (b) V-terminated surfaces of γ-NiFeV LDH. (c) Structures of different surface phases and OER intermediates on the Fe-terminated and V-terminated surfaces; OER intermediates are differentiated by colors (yellow instead of white for hydrogen and orange instead of red for oxygen, respectively). A dashed orange circle indicates the formation of a surface O vacancy. The reaction centers are highlighted by large white dotted circles. The magnetic moments of Ni and Fe during the OER are also indicated. (d) Reaction free-energy diagrams for OER on Fe- and V-terminated surfaces of γ-NiFeV LDH; the potential limiting steps and the overpotentials are also given.

For the Fe-terminated surface, the potential-determining step (PDS) is the deprotonation of the Fe^4+^–OH–Ni^4+^ bridge OH species, with a free energy barrier of 1.88 eV, corresponding to an overpotential of η = 0.65 V. This represents no improvement compared to γ-NiFe LDH (η = 0.45 V), which we attribute to the altered electronic structure of the reaction center: the presence of subsurface V stabilizes Ni in a higher oxidation state, shifting the reaction center from Fe^4+^–OH–Ni^3+^ as in γ-NiFe LDH to Fe^4+^–OH–Ni^4+^, which is less favorable for OER. This result demonstrates that subsurface V alone, without direct surface exposure, is insufficient to improve OER activity when Fe occupies the active bridge site.

In contrast, the V-terminated surface yields a reduced overpotential of 0.37 V (free energy barrier of 1.60 eV), an improvement of 0.08 V over γ-NiFe LDH, which is semiquantitatively consistent with the experimentally observed overpotential shift of ∼27 mV at 10 mA/cm^2^. On this surface, the active reaction center is the V^5+^–OH–Ni^2+^ bridge site, where the PDS is again OH* deprotonation, but now accompanied by Ni^2+^ oxidation rather than Fe oxidation. The key insight is that surface V^5+^, by virtue of its high oxidation state and strong electronegativity, acts as an electronic modulator that tunes the oxidation state of the neighboring Ni center, enabling a more favorable Ni-based redox pathway for OER. Crucially, the oxidation state of V itself remains unchanged throughout the OER cycle. Taken together, these results reveal that the OER enhancement in NiFeV LDH is governed by the surface exposure of V, which electronically activates the adjacent Ni center, while subsurface V alone is insufficient. Further computational details including surface phase diagrams, chemical potential corrections, and reaction free energy profiles are provided in the Supporting Information.

### 3.5 Chemical Stability of NiFeX LDH

To further study the activation of the catalysts and their dynamic behavior under operating conditions, we conducted metal ion stability measurements of the NiFeX LDH samples. In this section, we focused on leaching of the selected “X” metals (i.e., V, Mo, and W), since it has been reported that leaching of Ni is minor, while leaching and redeposition of Fe has been described in detail before by Tyndall et al., Bao et al, and Chung et al. respectively.^46–48^ Our goal is to support our conclusions and explanations for the distinct trends in geometric current densities, including the absence of intrinsic activity enhancement. Chemical stability measurements were performed using ICP-MS in a fully glass-free PTFE RDE cell before and after activation by cyclic voltammetry (full protocol in Supporting Methods 6). In these experiments, we were able to show that a large portion of the ternary metal “X” was present into the electrolyte after the electrochemical experiments (see Supporting Figure 19). NiFeMo, NiFeW and NiFeV LDH were selected as examples, being the three most active catalysts in the geometric current density trend. After cycling the potential including in the OER potential regime, up to 40% of the initial atomic W content was present in the electrolyte, with Mo and V in considerable amounts, as well, up to 33% and 46%, respectively, which is consistent with the calculated metastability of NiFeV described before. Additionally, Supporting Figure 20 shows that intrinsic activity trend seems to be dependent on Fe content (at %) very similarly to binary NiFe LDH.^6^ To provide a more comprehensive perspective on catalyst stability under OER conditions, we performed identical location SEM analysis combined with EDX measurements (Supporting Methods 2.2) before and after RDE testing on glassy carbon electrodes coated with NiFe, NiFeMo, NiFeV, and NiFeCr LDH (Supporting Figures 22–25).

The SEM comparison reveals noticeable morphological evolution after electrochemical testing, including partial delamination of the catalyst layers in all NiFeX systems. Importantly, distinct morphological differences are already present prior to electrochemical testing. For example, NiFeV LDH and NiFeMo LDH samples exhibited a more interconnected, sheet-like morphology, whereas NiFe LDH and NiFeCr LDH samples appeared more discontinuous and less uniformly distributed on the electrode surface. Interestingly, these initial structural differences were preserved to varying extent after RDE testing, although all samples underwent some degree of electrochemically induced restructuring and exhibited intense delamination, similar to that observed for NiFeV and NiFeMo LDHs in the single-cell tests. The absence of an abrupt decrease in CV profiles during consecutive CV cycles (Supporting Figure 26) suggests that the effect of delamination of the catalyst layer on ECSA values is limited in the time scale of these experiments. The delaminated part most likely consisted of a loosely bound fraction of the catalyst layer that did not undergo electrochemical activation. However, this delamination can lead to overestimation of the leaching of X through ICP-MS measurements of the electrolyte.

For this reason, EDX analysis (see Supporting Table 5) was performed on the same electrode locations before and after testing. The results indicate a slight decrease in the relative content of the incorporated X species compared to Ni and Fe. For example, 46% V leaching was estimated from ICP-MS of the electrolyte, while only 14% is observed with EDX of the electrode. Despite EDX being semiquantitative and surface-sensitive, and therefore not fully reliable for precise compositional changes, particularly in porous and rough catalyst layers, these complementary measurements confirm partial leaching of X metals during electrochemical operation. The leaching of the “X” metals likely partially accounts for the exceptionally high ECSAs observed in the NiFeX LDH catalysts (see Figure 3a), especially for NiFeMo and NiFeW LDHs. We note that cation vacancies can form as a result of X leaching within the NiFeX LDH lattice. Previous studies have shown that cationic vacancies improve stability and activity of NiFe-based LDHs by modifying the local electronic structure and increasing the number of catalytically accessible sites.^49,50^ Decoupling the cationic-vacancies-related effects is a challenging and future research direction in understanding and developing stable NiFe-based OER catalysts.

Previous studies have demonstrated the excellent long-term durability of NiFe LDHs up to 1000 h under OER conditions.^51^ Furthermore, the incorporation of high-valent transition metals into NiFe-based LDH catalysts can further improve the stability of activated catalysts by strengthening the metal–oxygen frame. For example, Vanadium-doped NiFe LDH has been reported to sustain stable operation up to 240 h in 1.0 M KOH.^52^ Stability tests of NiFeX LDHs on longer time scales comparable to NiFe LDHs are required to fully validate the long-term durability of this beneficial effect.

Taken together, our results indicate that the incorporation of different X elements influences both the initial catalyst-layer morphology and its structural evolution under OER conditions, including adhesion and the delamination of catalyst layers. Further studies combining quantitative dissolution analysis (e.g., ICP-MS of electrolyte), operando structural characterization, and controlled electrode architecture will be required to fully elucidate the interplay between leaching, reconstruction, and catalyst-layer integrity in AEM-based OER systems.

## 4 Conclusion

In this study, we conducted a systematic screening of the intrinsic OER activity of rigorously phase-pure ternary NiFeX LDHs and correlated their performance to experimental and computational electronic structure investigations. The third metal X was selected from high-valence transition metals across groups 5 and 6 (V, Nb, Ta, Cr, Mo, and W), incorporated into the NiFe LDH host structure with carefully controlled stoichiometry and crystal structure—a level of material control that sets the present study apart from previous approaches.

While NiFeMo LDH and NiFeV LDH demonstrated significant improvements in geometric current densities, normalization to their real electrochemically active surface area unambiguously revealed that none of the ternary NiFeX LDH catalysts exhibited enhanced intrinsic OER activity compared to the NiFe LDH reference within the margin of error. This key finding establishes that geometric activity enhancements in NiFeX LDH are not of intrinsic electronic origin at the active site level. XPS confirmed that the incorporation of X did not significantly modify the chemical states of Ni or Fe in the as-prepared precatalysts. In situ HERFD-XAS measurements under OER-relevant potentials, however, revealed that the incorporation of X influences the extent and, in the case of V, the electronic character of the α to γ activation behavior. DFT calculations confirmed this interpretation and provided atomistic insight into the Ni oxidation states under catalytic conditions.

Among all investigated catalysts, NiFeV LDH exhibited a uniquely distinct activation behavior, characterized by a blue shift of the γ-phase pre-edge peak 2 of the Ni K-edge to lower energies and a clear shift of the edge position itself, indicating an electronically modified γ-phase. DFT calculations rationalize this observation through the role of surface V^5+^ as an electronic modulator at the V^5+^–OH–Ni^2+^ bridge site, which lowers the OER overpotential by enabling a more favorable Ni-based redox pathway. The resulting smaller Tafel slope of NiFeV LDH might reflect this mechanistically distinct pathway and contribute to its superior geometric activity. In stark contrast, incorporation of Cr was found to completely suppress the formation of the active γ-phase, rationalizing its inferior OER performance. These findings collectively demonstrate that the nature of the incorporated metal X governs the α- to γ-phase transition, which emerges as a critical descriptor of OER activity in ternary NiFeX LDH systems.

The activity enhancements of NiFeV LDH, driven by its unique electronic promotional effect, and NiFeMo LDH, driven by its substantially increased ECSA, were successfully translated to 25 cm^2^ active-area anion exchange membrane water electrolyzer single-cells, confirming the practical relevance of the identified mechanisms and the promise of these materials for large-scale alkaline water electrolysis.

## Supplementary Material



## Data Availability

The data supporting the findings of this study are available within this Article and its Supporting Information files or from the corresponding author upon reasonable request. Data produced at the ESRF facility can be accessed through ESRF from the year 2027.^53^

## Notes

Descriptions of the methods used; XAS experiments; normalized XRD patterns of as-synthesized Ni_a_Fe_1_V_1_ LDH; TEM image; XPS spectra of “X” metals; Fe-K-edge spectra of NiFeX LDHs at 1.1 V; SEM before and after RDE experiments of NiFeMo LDH coated glassy carbon electrode; OER mechanism on the V-terminated γ-NiFeV LDH surface; summary of metal content obtained from ICP-OES; EDX derived chemical composition; verifying the origin of large ECSA values of Ni_a_Fe_1_Mo_1_ and Ni_a_Fe_1_W_1_ LDHs; discussion of the Cell used in the XAS Experiments at ID26; beam damage and mitigation thereof; discussion on the NiFeV LDH DFT calculations (PDF)
