# Supplementary material for: Disentangling Electronic and Geometric Effects in Ternary NiFeX Layered Double Hydroxide Catalysts for Oxygen Evolution
Source: J Am Chem Soc. 2026 Sep 7;148(37):40198–212. doi: 10.1021/jacs.6c12678 (PMC13614199; doi:10.1021/jacs.6c12678)
Supplement: Supplementary file 1 [file ja6c12678_si_001.pdf]

Supporting Information for

Disentangling Electronic and Geometric Effects in Ternary NiFeX Layered Double Hydroxide Catalysts for Oxygen Evolution

D. Choi,[+,1] P. W. Buchheister,[+,1] R. Somaiya,[+,2,3] D. Ligay,[1] G. Podbielski,[1] J. Lu,[1] P. Schröer,[1] E. Bumenn,[4] I. Galkina,[5] F. Scheepers,[5] Z. Brejwo,[1] A. Hartmann,[1] B. Paul,[1] T. N. Thanh,[1] M. Klingenhof,[1] A. Karl,[4] E. Jodat,[4] R.-A. Eichel,[4] Z. Zeng,[*,3] F. Dionigi,[*,1] P. Strasser[1]

[1] The Electrochemical Energy, Catalysis, and Materials Science Laboratory, Department of Chemistry, Chemical Engineering Division, Technical University Berlin, Straße des 17. Juni 124, 10623 Berlin, Germany.

[2] School of Physics and Electronics, Hunan University, Changsha, 410082, China

[3] Tarpo Department of Chemistry, Davidson School of Chemical Engineering, School of Materials Engineering, Institute of Physical AI, Purdue University, West Lafayette, IN 47907, USA

[4] Institute of Energy Technologies, IET-1: Fundamental Electrochemistry, Forschungszentrum Jülich GmbH, Wilhelm-Johnen-Straße, 52428 Jülich.

[5] Institute of Energy Technologies, IET-4: Electrochemical Process Engineering, Forschungszentrum Jülich GmbH, Wilhelm-Johnen-Straße, 52428 Jülich.

[+] These authors contributed equally to this work.

[*] E-mail: fabio.dionigi@tu-berlin.de; zeng46@purdue.edu

Supporting Information

Supplementary Figures

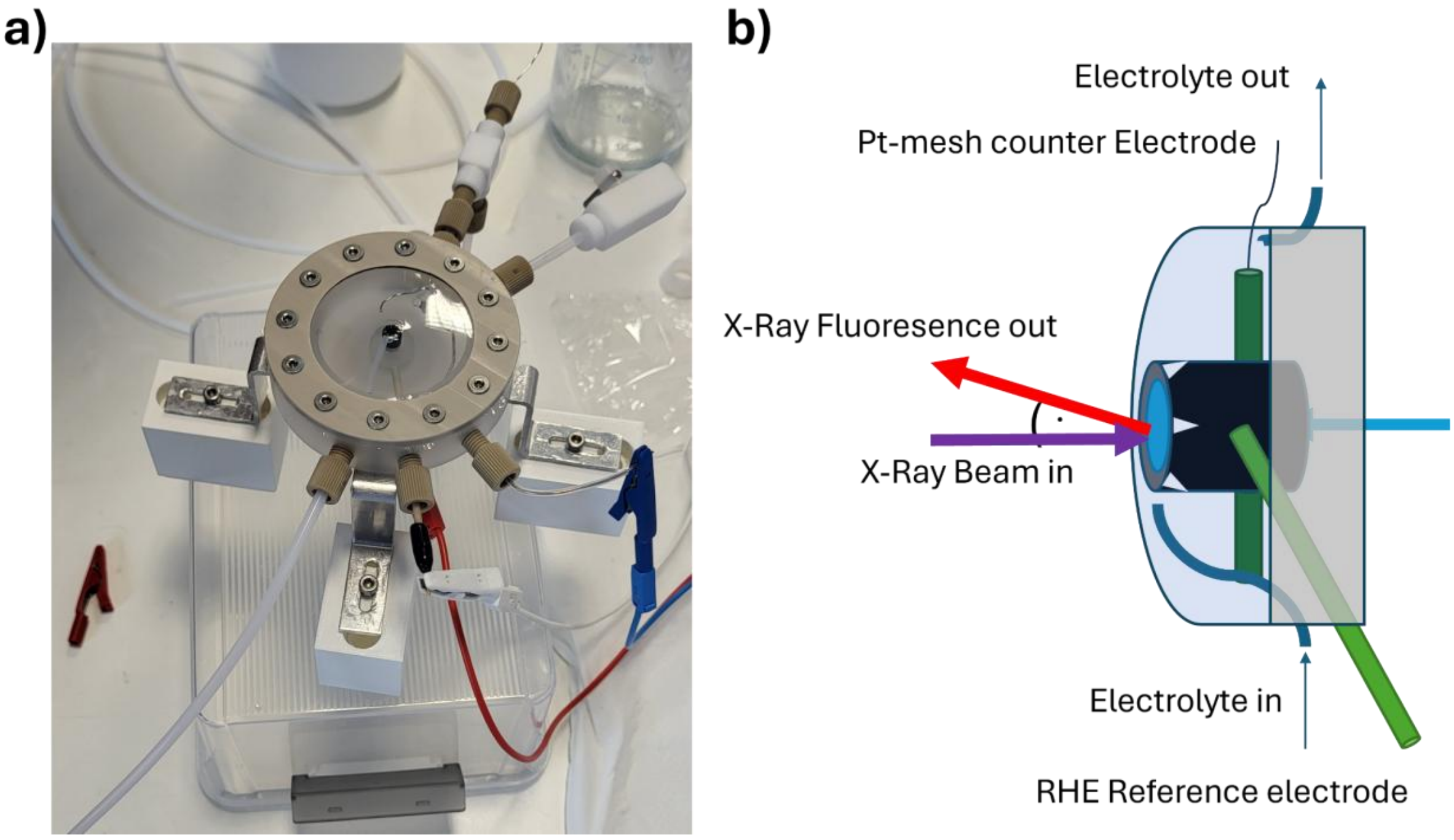

Supplementary Figure 1. a) Picture and **b)** annotated schematics of the *in-situ* electrochemical cell used at ID26 for the XAS experiments

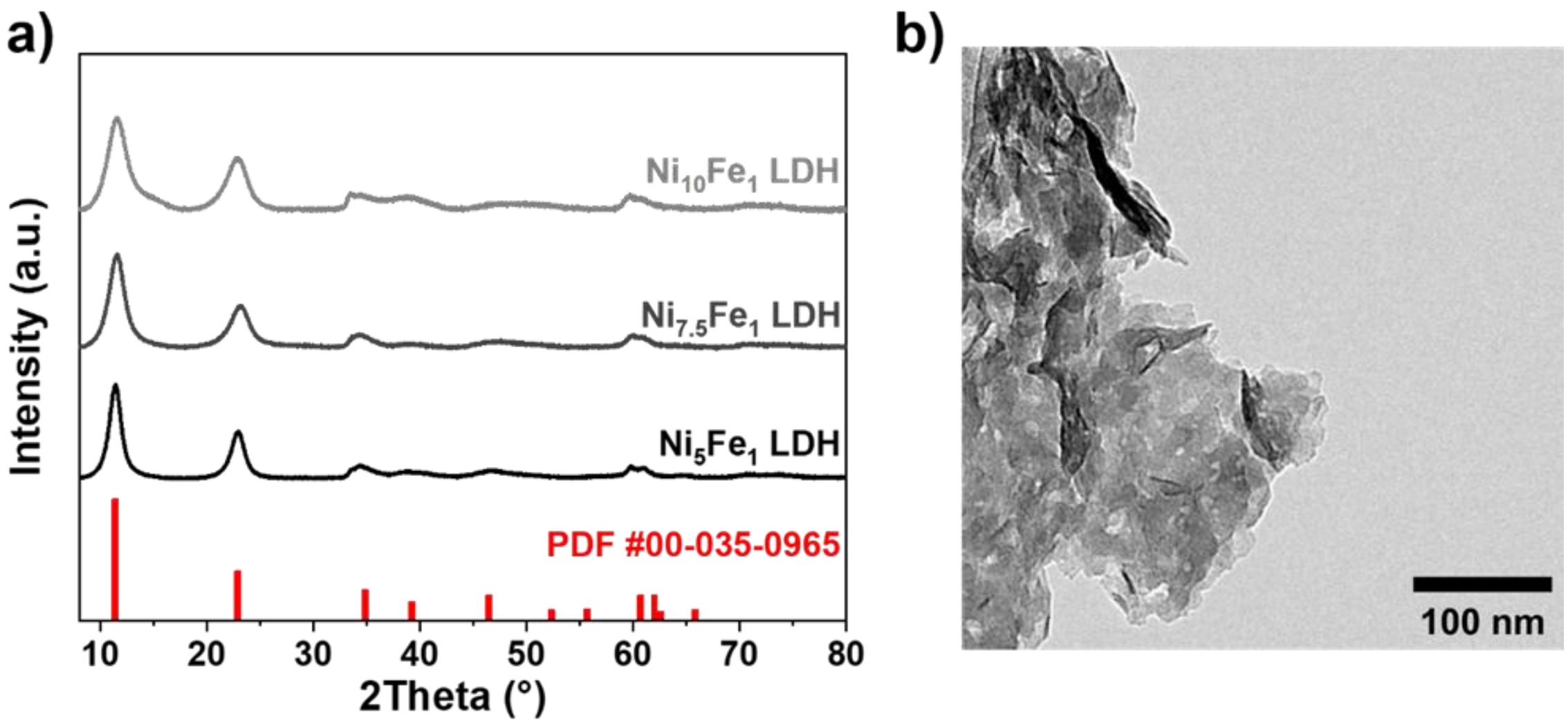

Supplementary Figure 2. a) normalized XRD patterns of as-synthesized Ni_aFe_1 LDH. The reference XRD pattern of hydrotalcite structure is also shown in red (PDF #00-035-0965). **b)** TEM image of $Ni_{7.5}Fe_1$ LDH.

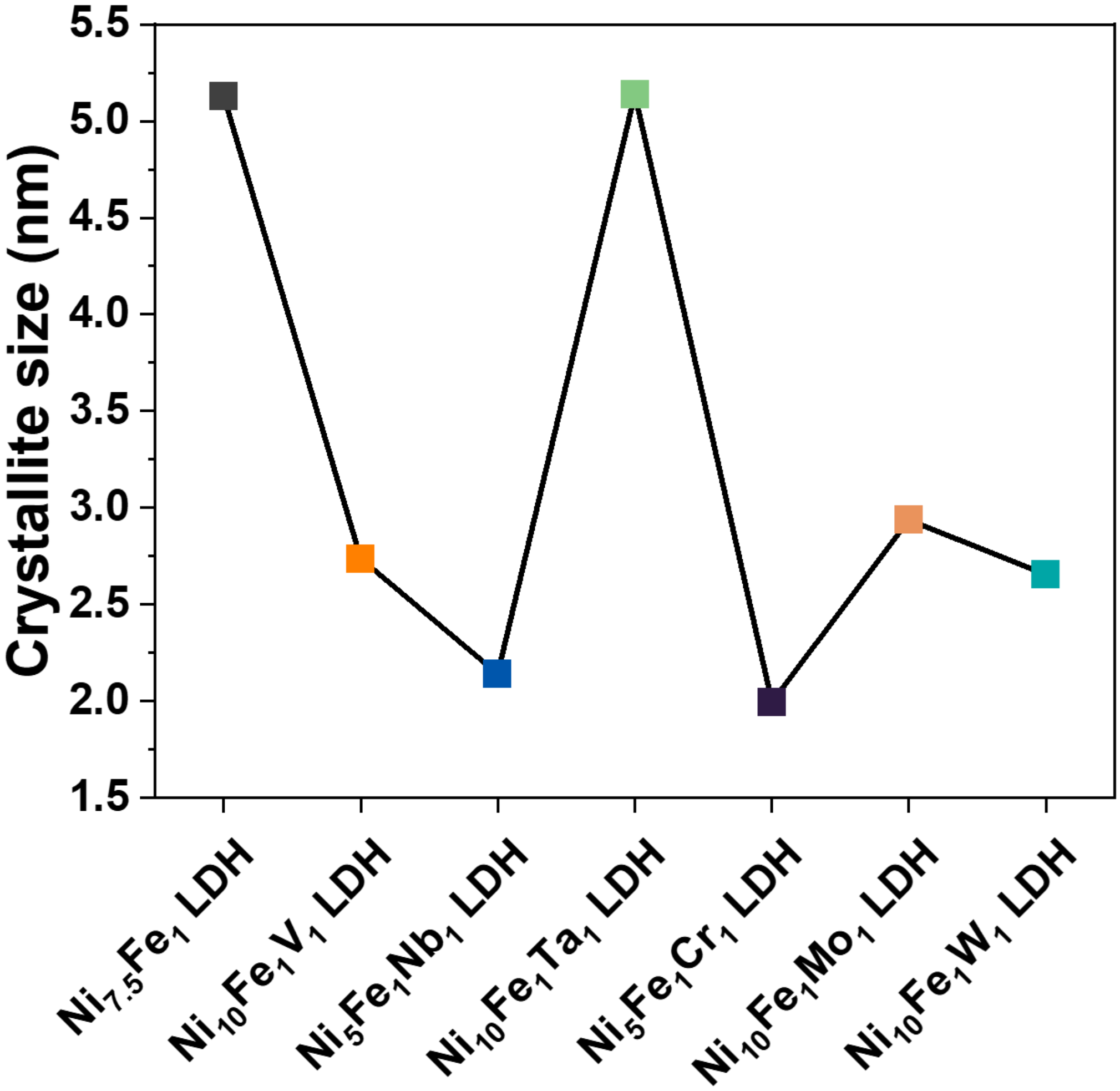

Supplementary Figure 3. Crystallite size of the catalytically most active Ni_aFe_1 LDH and $Ni_aFe_1X_1$ LDHs chosen based on their geometric overpotential at 10 mA/cm^2 in 0.1 M KOH. Crystallite size for each material is calculated based on (003) peak using Scherrer equation. All ternary $Ni_aFe_1X_1$ LDHs, apart from $Ni_{10}Fe_1Ta_1$ LDH, show smaller crystallite size compared to $Ni_{7.5}Fe_1$ LDH, which we hypothesized been caused by incorporation of X into the NiFe LDH host structure. The exception of $Ni_{10}Fe_1Ta_1$ LDH is consistent with this hypothesis, due to the small X content (3 at% Ta), in contrast to the other ternary NiFeX LDH samples (see Supplementary Table 1).

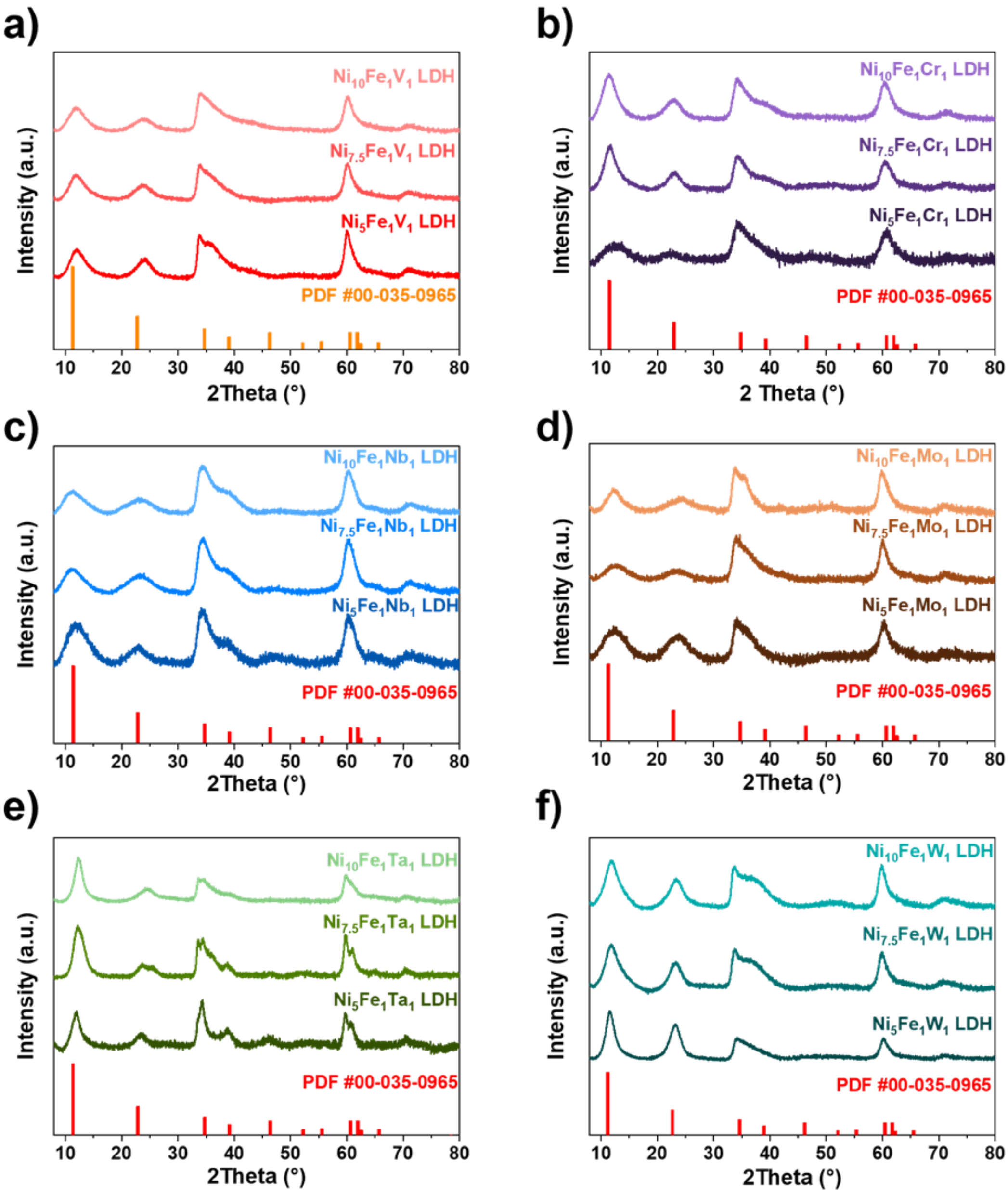

Supplementary Figure 4. a), **c)**, **e)** normalized XRD patterns of as-synthesized $Ni_aFe_1V_1$ LDH **a)**, $Ni_aFe_1Nb_1$ LDH **c)**, $Ni_aFe_1Ta_1$ LDH **e)** for Group 5. **b)**, **d)**, **f)** normalized XRD patterns of as-synthesized $Ni_aFe_1Cr_1$ LDH **b)**, $Ni_aFe_1Mo_1$ LDH **d)**, $Ni_aFe_1W_1$ LDH **f)** for Group 6. The reference XRD pattern of hydrotalcite structure is also shown in red, but in orange in **a)** (PDF #00-035-0965).

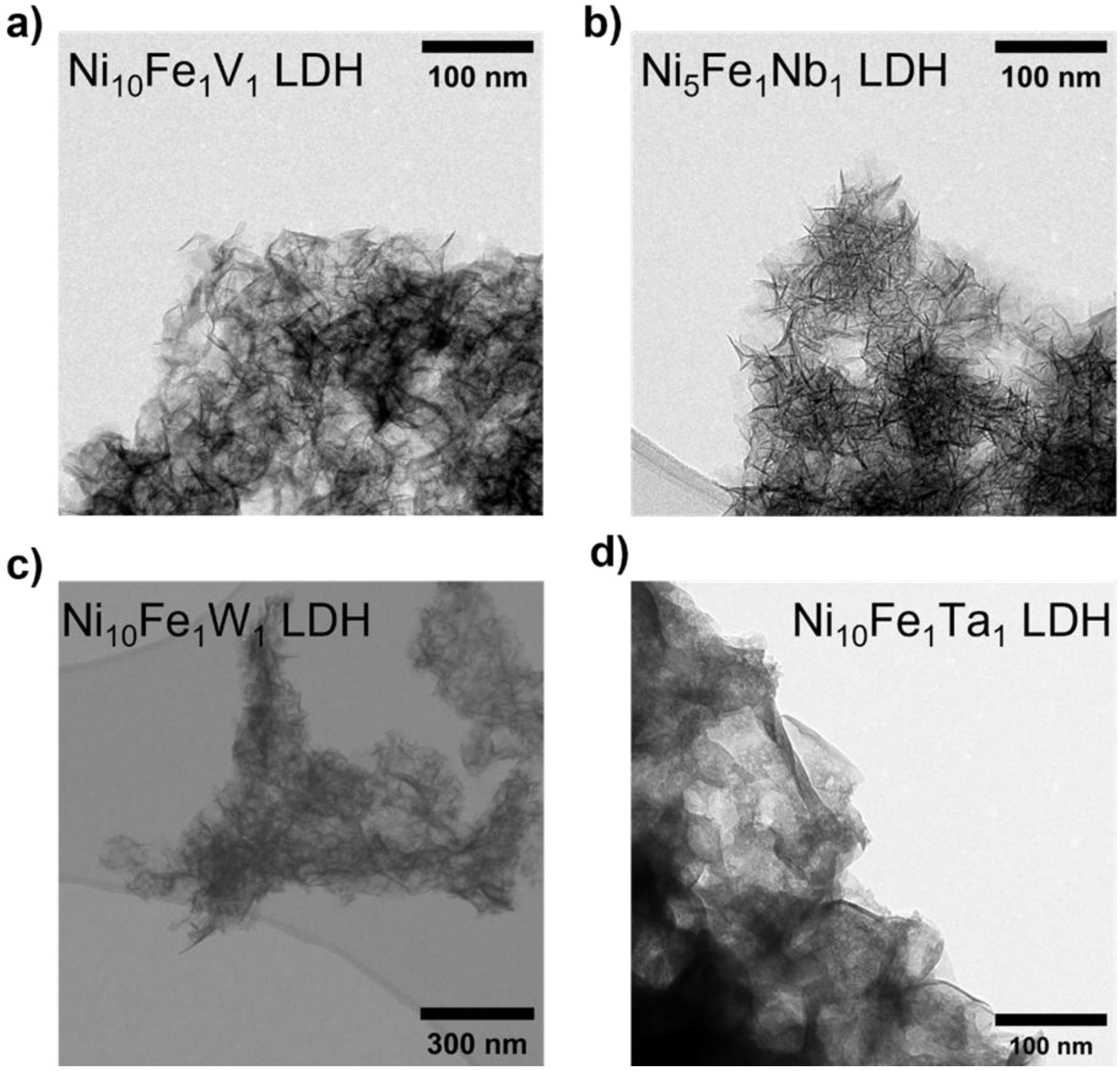

Supplementary Figure 5. a), b), c), d) TEM image of $Ni_{10}Fe_1V_1$ LDH, $Ni_5Fe_1Nb_1$ LDH, $Ni_{10}Fe_1W_1$ LDH, $Ni_{10}Fe_1Ta_1$ LDH, respectively.

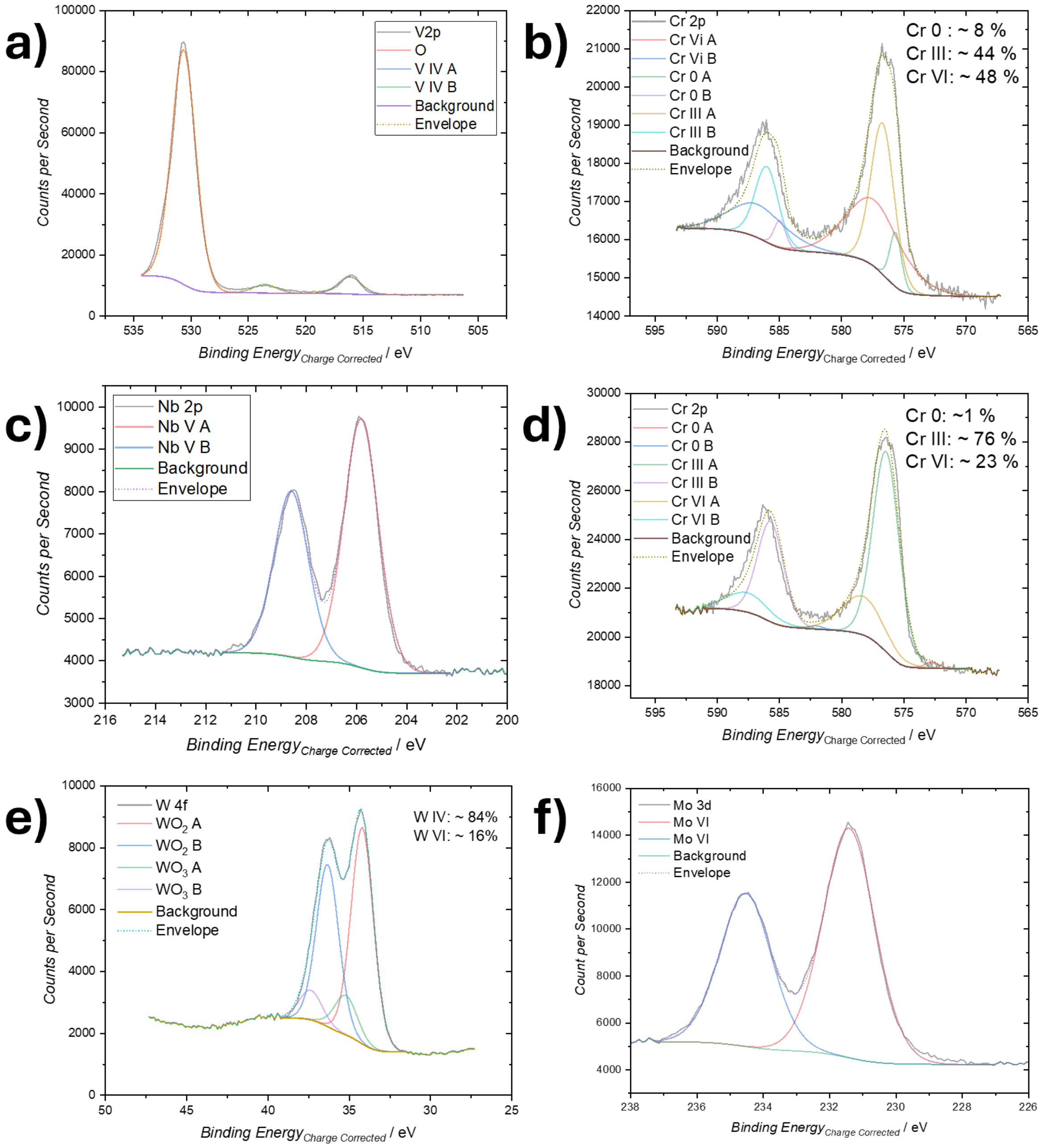

Supplementary Figure 6. XPS spectra of 'X' metals. Charge corrected and fitted XPS spectra of **a)** NiFeV (V 2p), **b)** NiFeCr-A (Cr 2p), **c)** NiFeNb (Nb 3d), **d)** NiFeCr-B (Cr 2p), and **e)** NiFeW (W 4f) **f)** NiFeMo (Mo 3d)

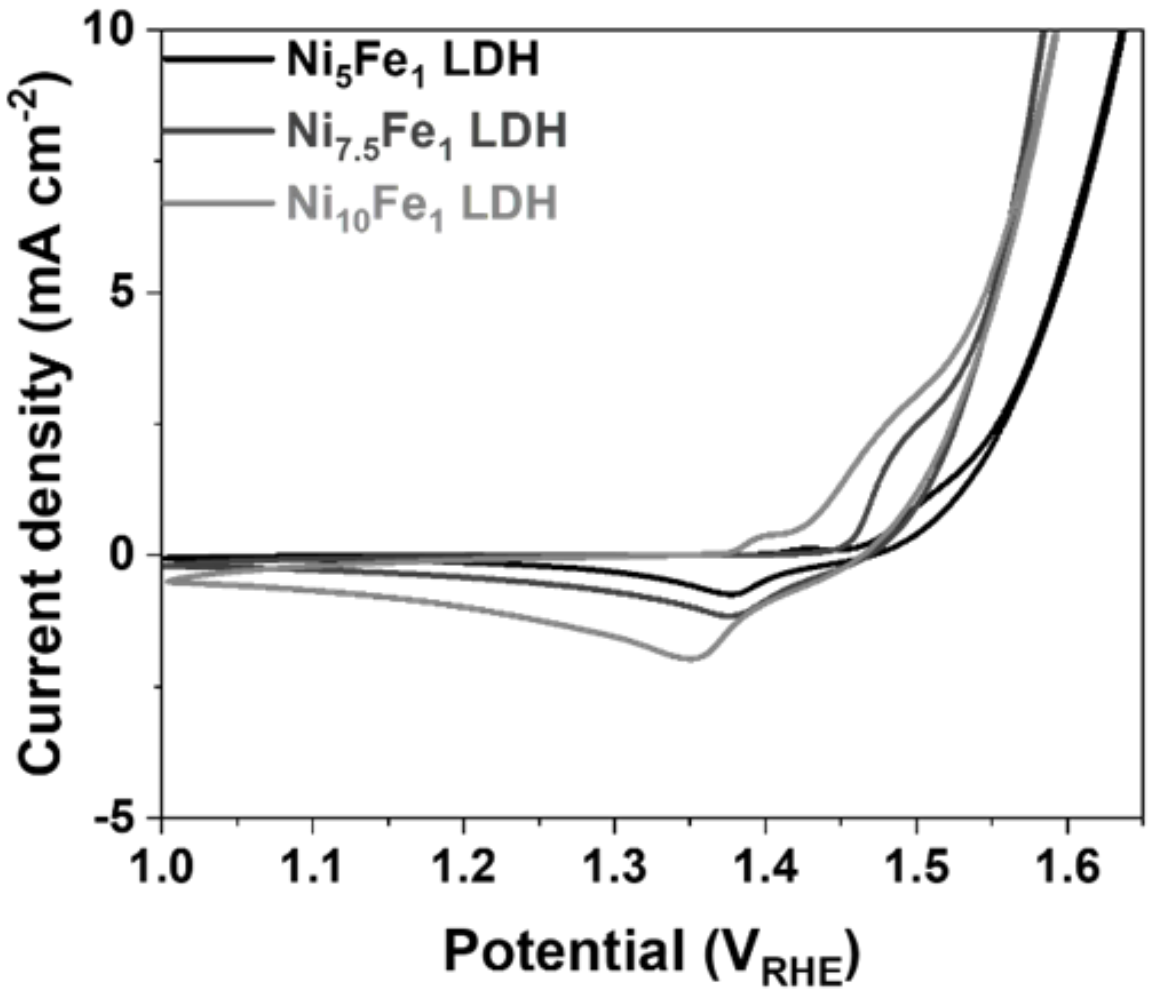

Supplementary Figure 7. 50th cycles of cyclic voltametric curves (CVs) of Ni_aFe_1 LDH. Cyclic voltammetry (CV) was performed at a scan rate of 50 mV/s in 0.1 M KOH by RDE (1600 rpm). Catalyst loading on GC electrodes: 0.1 mg/cm^2 (see detailed *Supplementary Methods* section *3.4*).

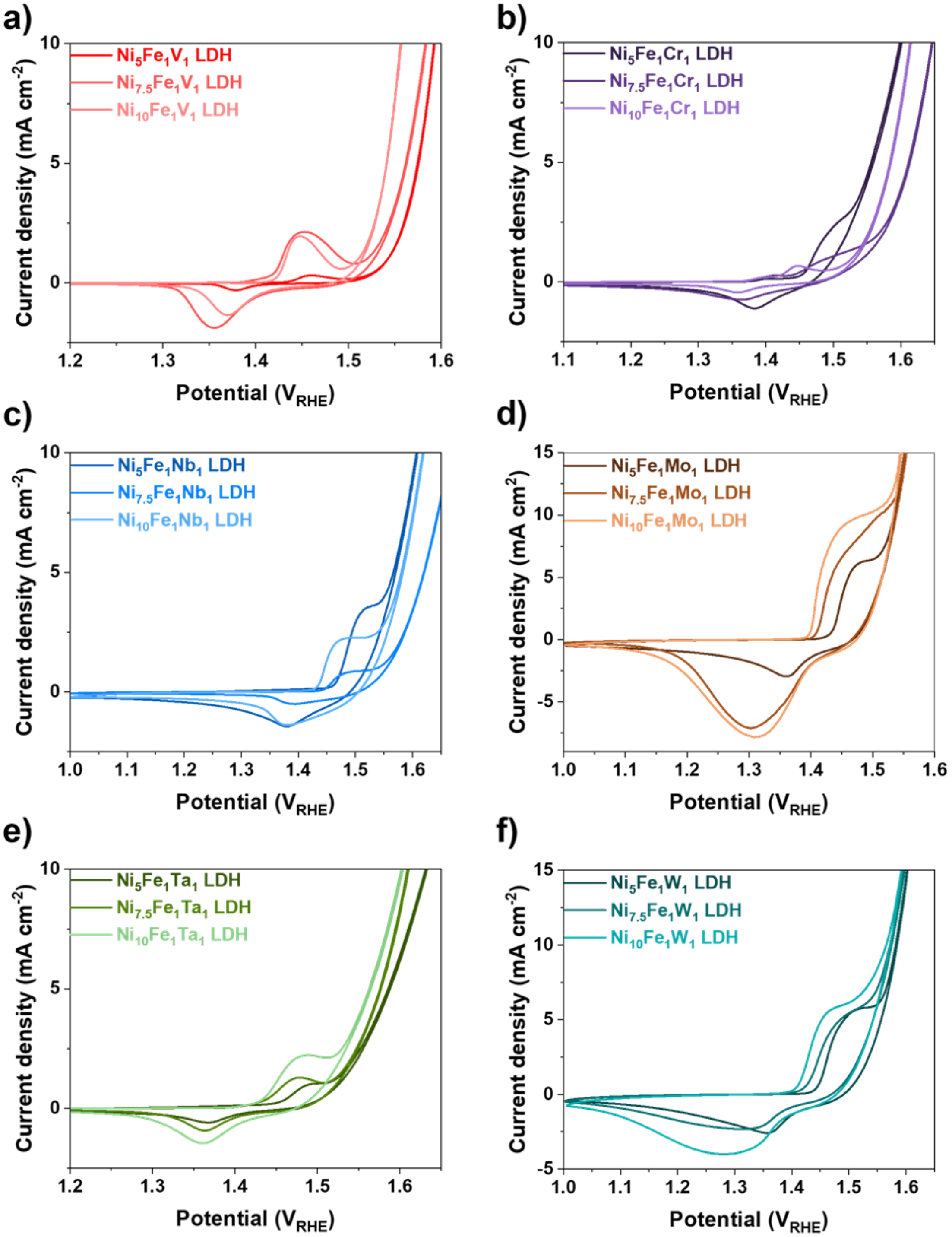

Supplementary Figure 8. 50th cycles of cyclic voltametric curves (CVs) of $Ni_aFe_1X_1$ LDHs in 0.1 M KOH. a) $Ni_aFe_1V_1$ LDHs, **c)** $Ni_aFe_1Nb_1$ LDHs, **e)** $Ni_aFe_1Ta_1$ LDHs for Group 5. **b)** $Ni_aFe_1Cr_1$ LDHs, **d)** $Ni_aFe_1Mo_1$ LDHs, **f)** $Ni_aFe_1W_1$ LDHs for Group 6.

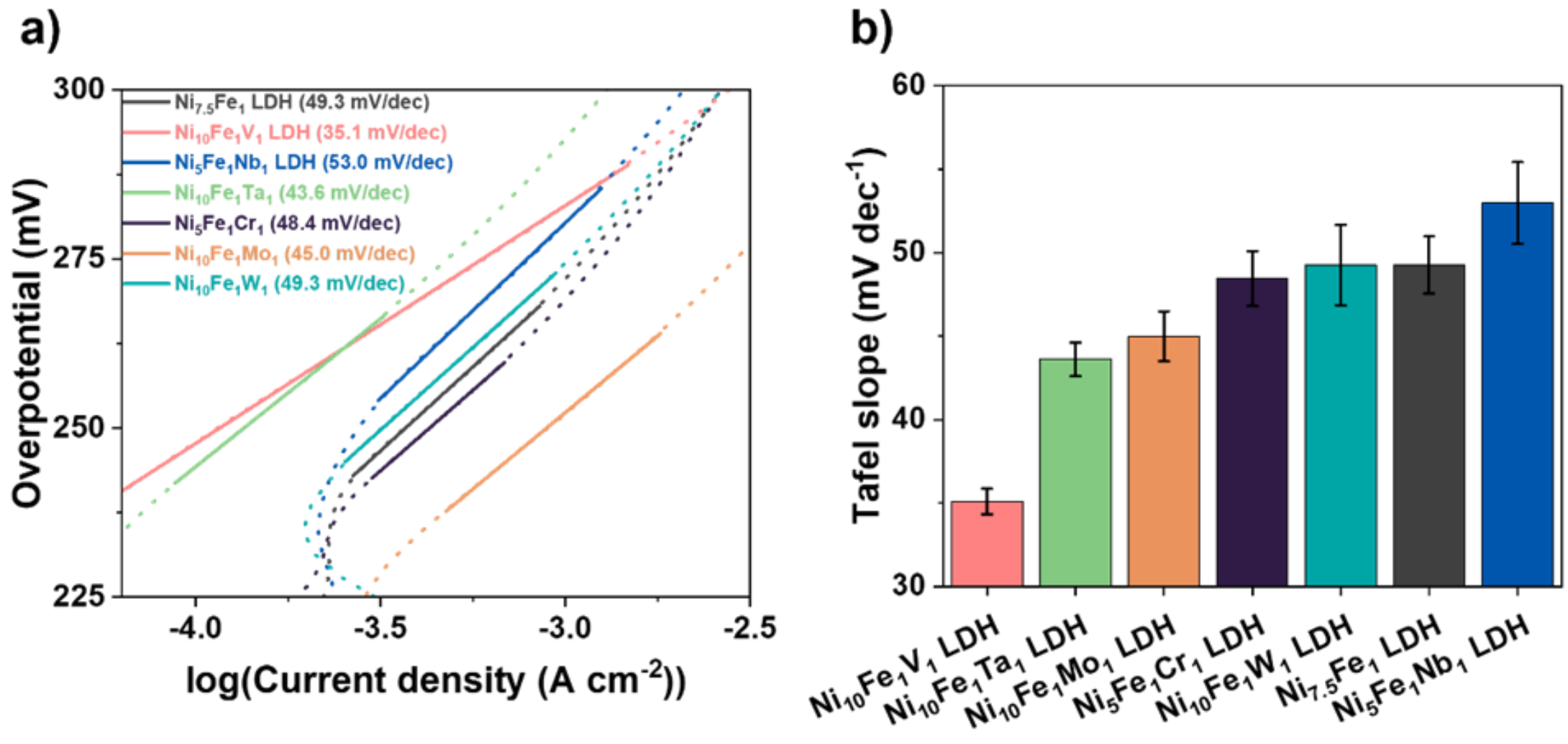

Supplementary Figure 9. Tafel plots of each most active Ni_aFe_1 LDH and $Ni_aFe_1X_1$ LDH based on the geometric overpotential at 10 mA/cm^2. a) Tafel plots in the low linear overpotential range, **b)** the corresponding Tafel slopes. The fitting is performed at least 3 times to obtain statistical average and its corresponding standard deviation. The error bars represent the statistical standard deviations.

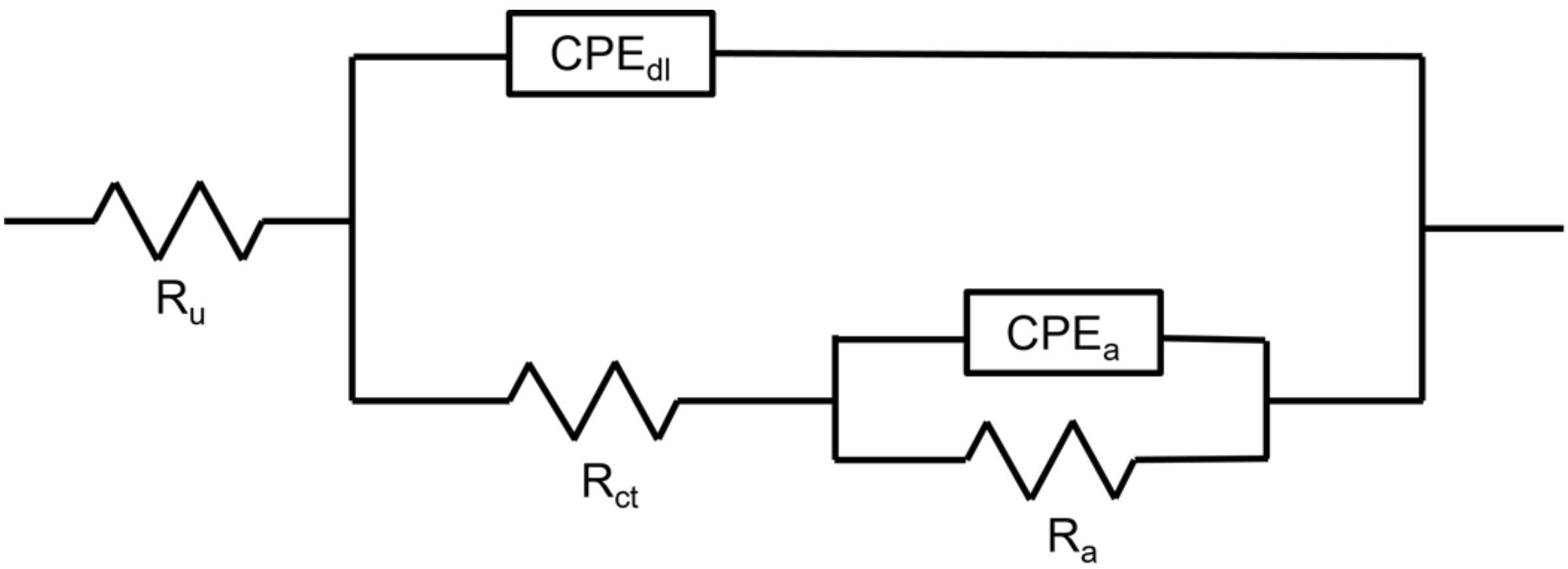

Supplementary Figure 10. Schematic representation of the equivalent electric circuit for fitting the Nyquist plot obtained from electrochemical impedance spectroscopy (EIS) in this study. The equivalent electric circuit consists of uncompensated resistance (R_u), charge transfer resistance (R_{ct}), double layer capacitance described as constant phase element (CPE_{dl}), OER adsorbates resistance (R_a), and OER adsorbates capacitance described as constant phase element (CPE_a).

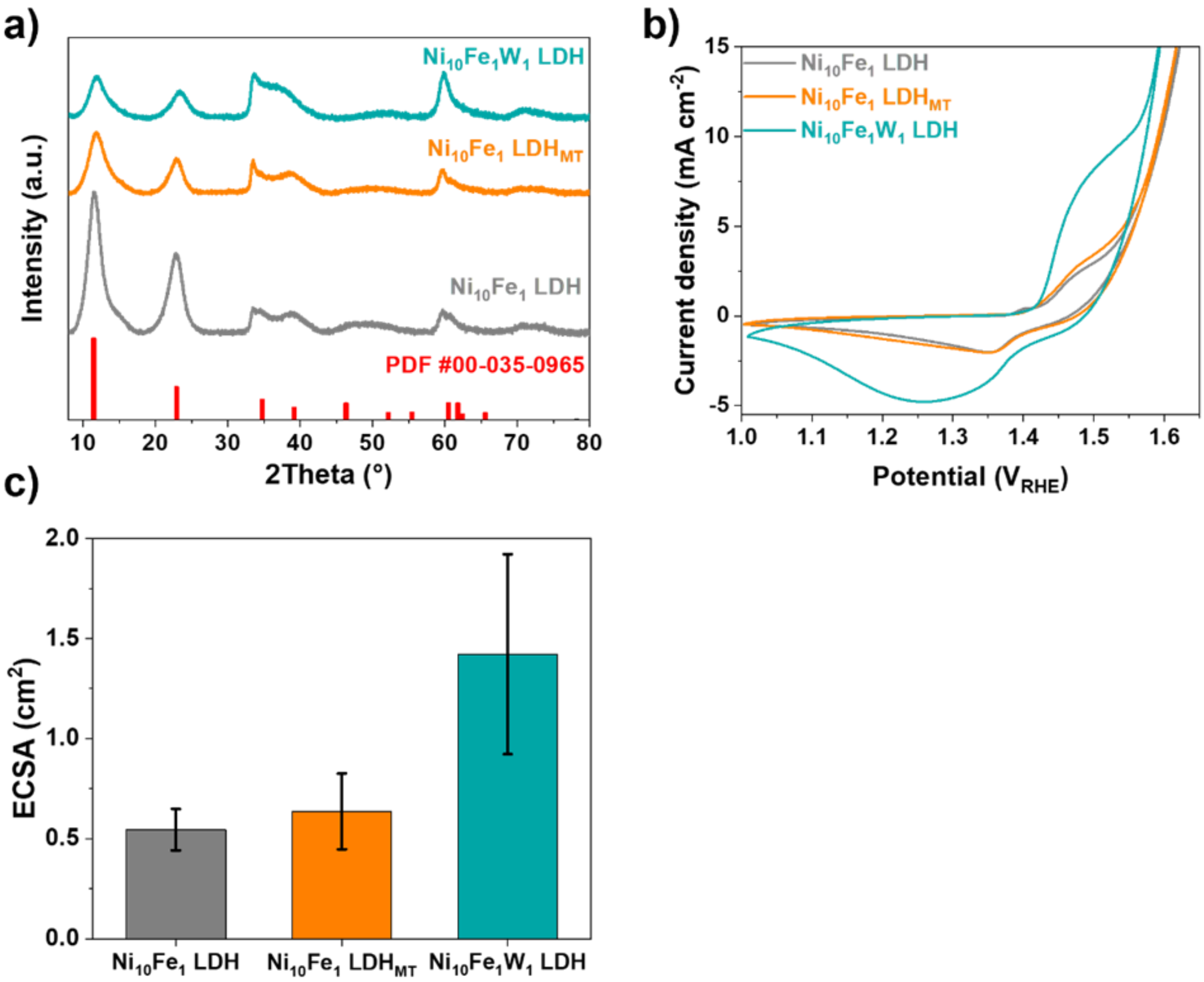

Supplementary Figure 11. Comparison of crystalline structures, surface redox behaviors, and ECSA values considering $Ni_{10}Fe_1$ LDH, $Ni_{10}Fe_1$ LDH_{MT}, and $Ni_{10}Fe_1W_1$ LDH. a) normalized XRD patterns of $Ni_{10}Fe_1$ LDH, $Ni_{10}Fe_1$ LDH_{MT} and $Ni_{10}Fe_1$ LDH. The reference XRD patterns of the hydrotalcite structure are also shown in red (PDF #00-035-0965), **b)** the corresponding 50th cycles of cyclic voltammetric curves (CVs), and **c)** the corresponding ECSA values obtained by electrochemical impedance spectroscopy (EIS). The statistical standard deviations are also shown in **c)**.

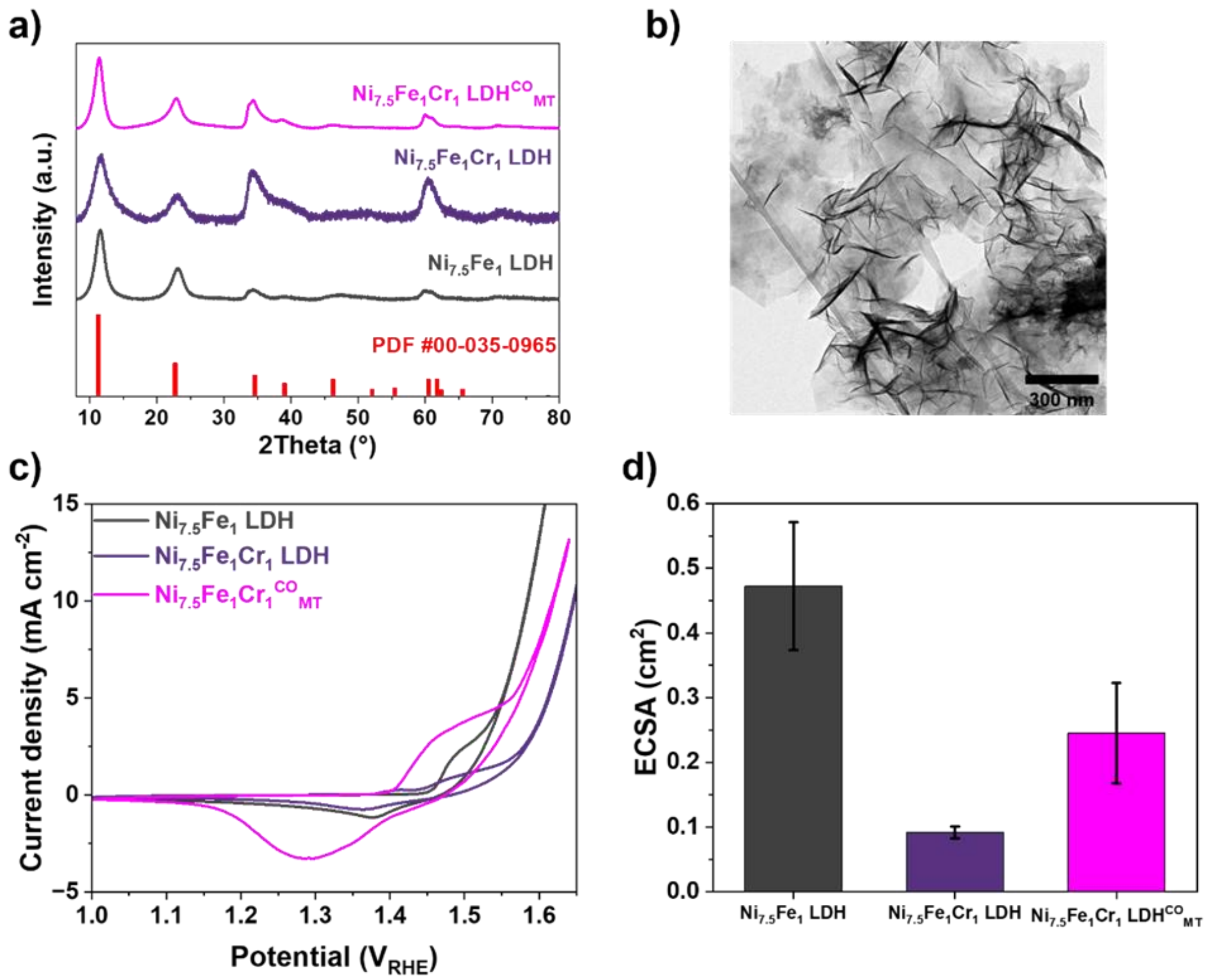

Supplementary Figure 12. Comparison of crystalline structures and morphology, surface redox behaviors and ECSA values considering $Ni_{7.5}Fe_1$ LDH, $Ni_{7.5}Fe_1Cr_1$ LDH, and $Ni_{7.5}Fe_1Cr_1$ LDH^{CO}_{MT}. a) normalized XRD patterns of $Ni_{7.5}Fe_1$ LDH, $Ni_{7.5}Fe_1Cr_1$ LDH, and $Ni_{7.5}Fe_1Cr_1$ LDH^{CO}_{MT}. The reference XRD pattern of the hydrotalcite structure is also shown in **a)** (PDF #00-035-0965). **b)** TEM image of $Ni_{7.5}Fe_1Cr_1$ LDH^{CO}_{MT}, **c)** 50th cycles of cyclic voltammetric curves (CVs) of $Ni_{7.5}Fe_1$ LDH, $Ni_{7.5}Fe_1Cr_1$ LDH, and $Ni_{7.5}Fe_1Cr_1$ LDH^{CO}_{MT}, and **d)** the corresponding ECSA values obtained by electrochemical impedance spectroscopy (EIS). The error bars represent the statistical standard deviations.

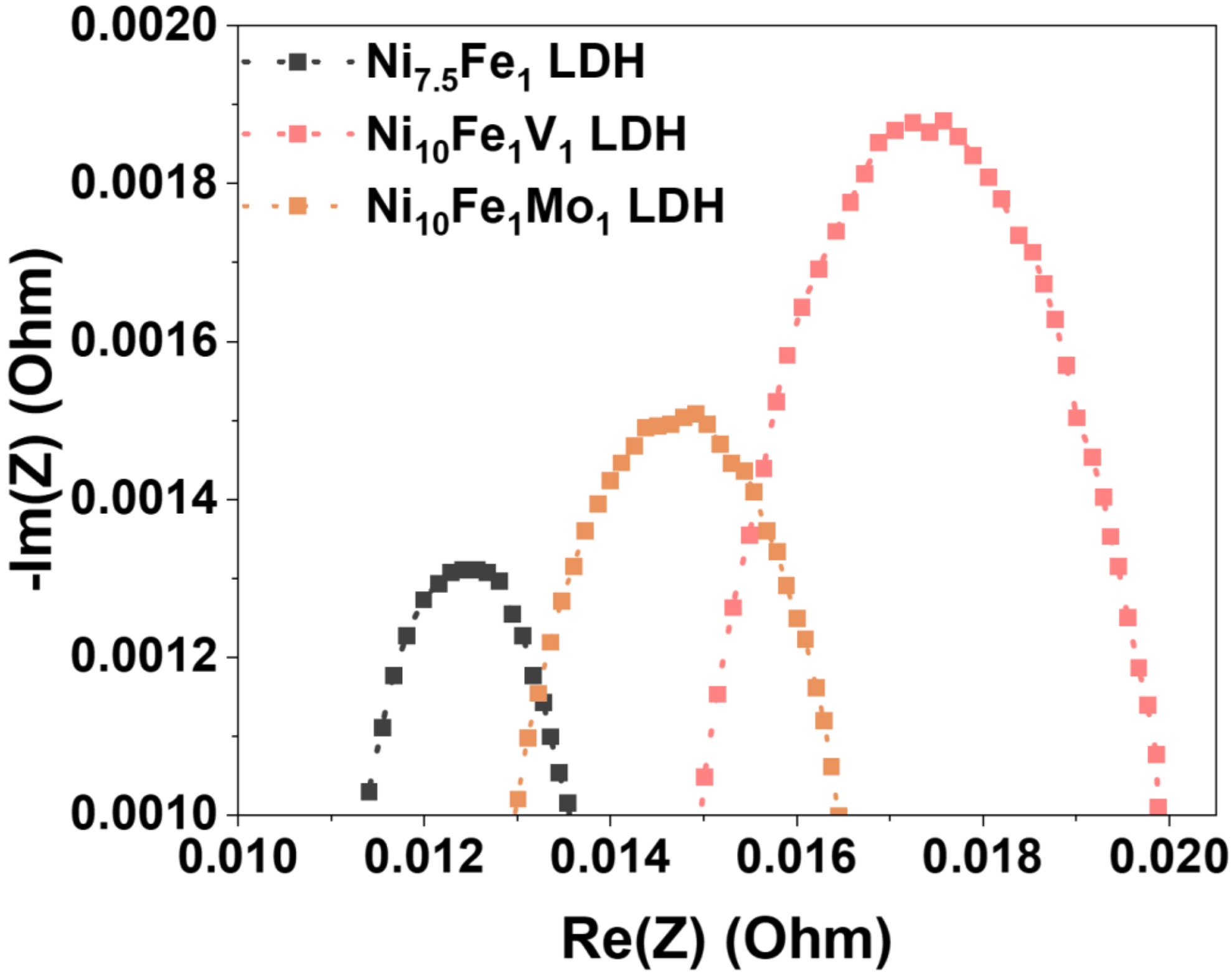

Supplementary Figure 13. Comparison of GEIS spectra for $Ni_{7.5}Fe_1$ LDH, $Ni_{10}Fe_1V_1$ LDH, and $Ni_{10}Fe_1Mo_1$ LDH in AEMWE single-cell measurements. Impedance spectra were obtained at 1 A/cm^2 over a frequency range of 5 kHz to 5 Hz using a current perturbation amplitude 2.5 A. (*see supplementary method 4*)

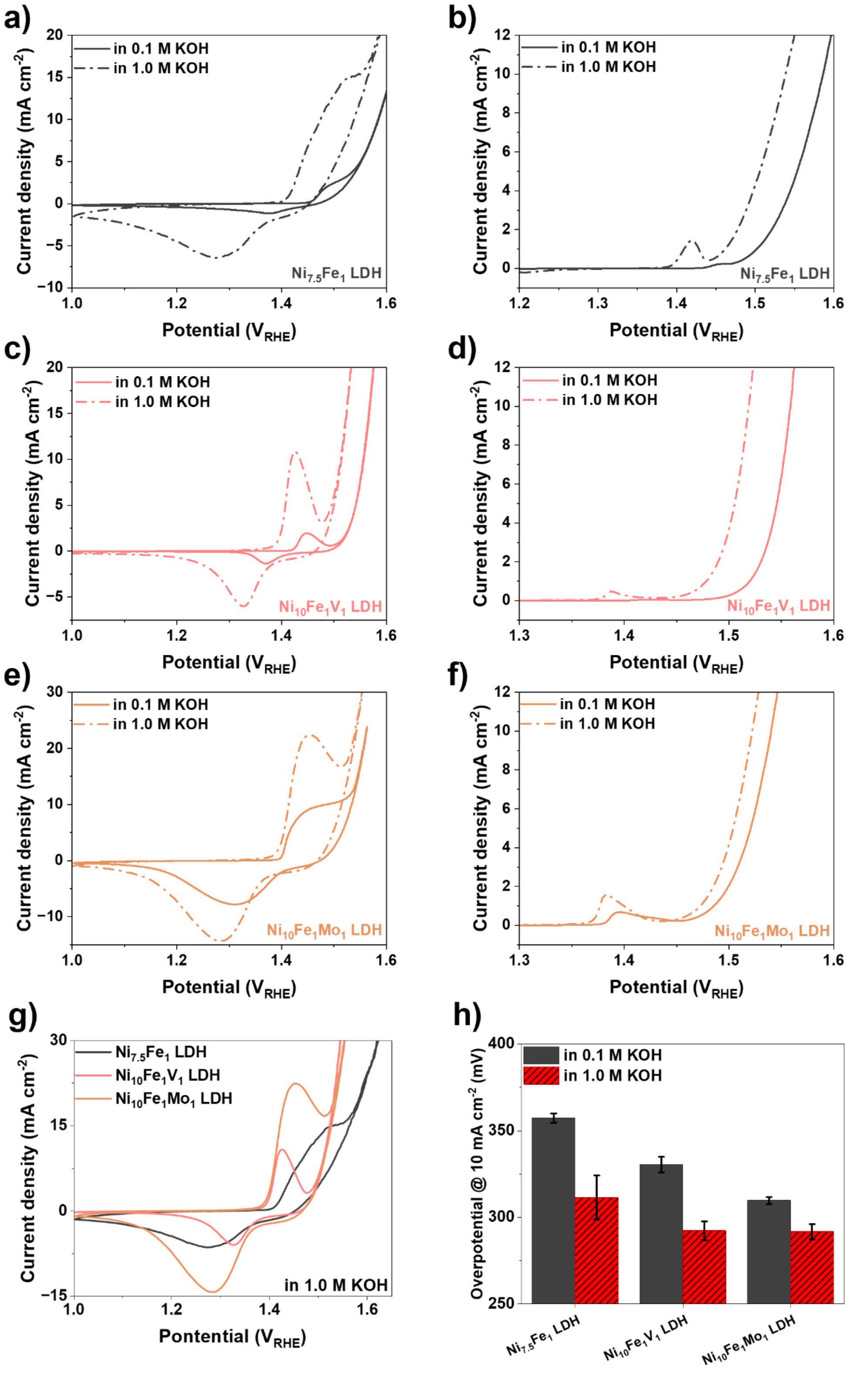

Supplementary Figure 14. Comparison of surface redox behaviors and geometric OER activities in 0.1 M KOH and in 1.0 M KOH considering $Ni_{7.5}Fe_1$ LDH, $Ni_{10}Fe_1V_1$ LDH, and $Ni_{10}Fe_1Mo_1$ LDH. 50th cycles of cyclic voltammetric curves (CVs, 50 mV/s) and the corresponding linear sweep voltammetric curves (LSVs, 1 mV/s) for **a)** and **b)** $Ni_{7.5}Fe_1$ LDH, **c)** and **d)** $Ni_{10}Fe_1V_1$ LDH, **e)** and **f)** $Ni_{10}Fe_1Mo_1$ LDH. **g)** Comparison of surface redox behaviors. **h)** Comparison of overpotentials at 10 mA/cm^2 in 0.1 M and 1.0 M KOH.

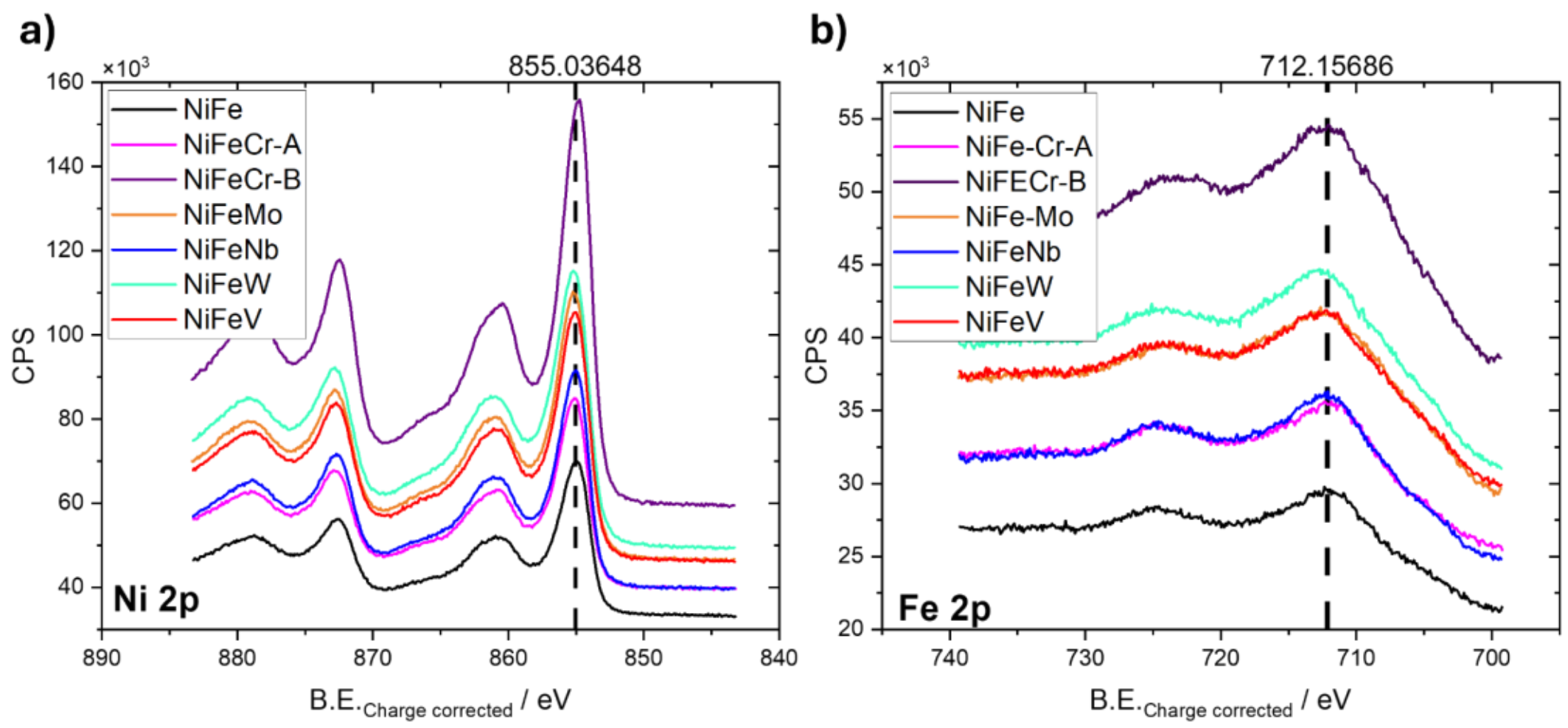

Supplementary Figure 15. Comparison between the a) Ni-2p XPS spectra and the b) Fe-2p XPS spectra of all investigated NiFe and NiFeX LDHs. The dotted line serves as a visual guide to the eye and marks the peak position in the NiFe LDH reference material. All spectra have been charge-corrected.

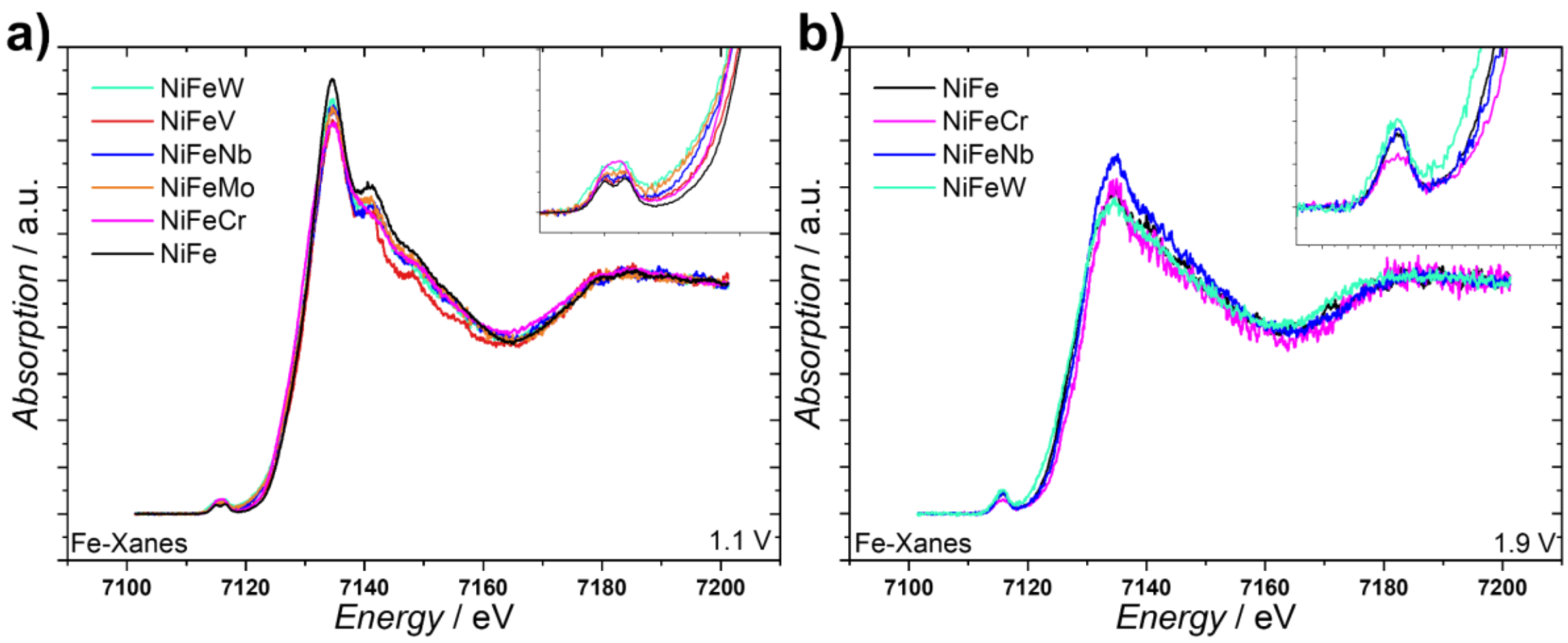

Supplementary Figure 16. Fe-K-edge spectra of NiFeX LDHs at 1.1 V a) and 1.9 V b), respectively. Note that not all catalysts could be measured at 1.9 V due to time constraints during the allotted beamtime.

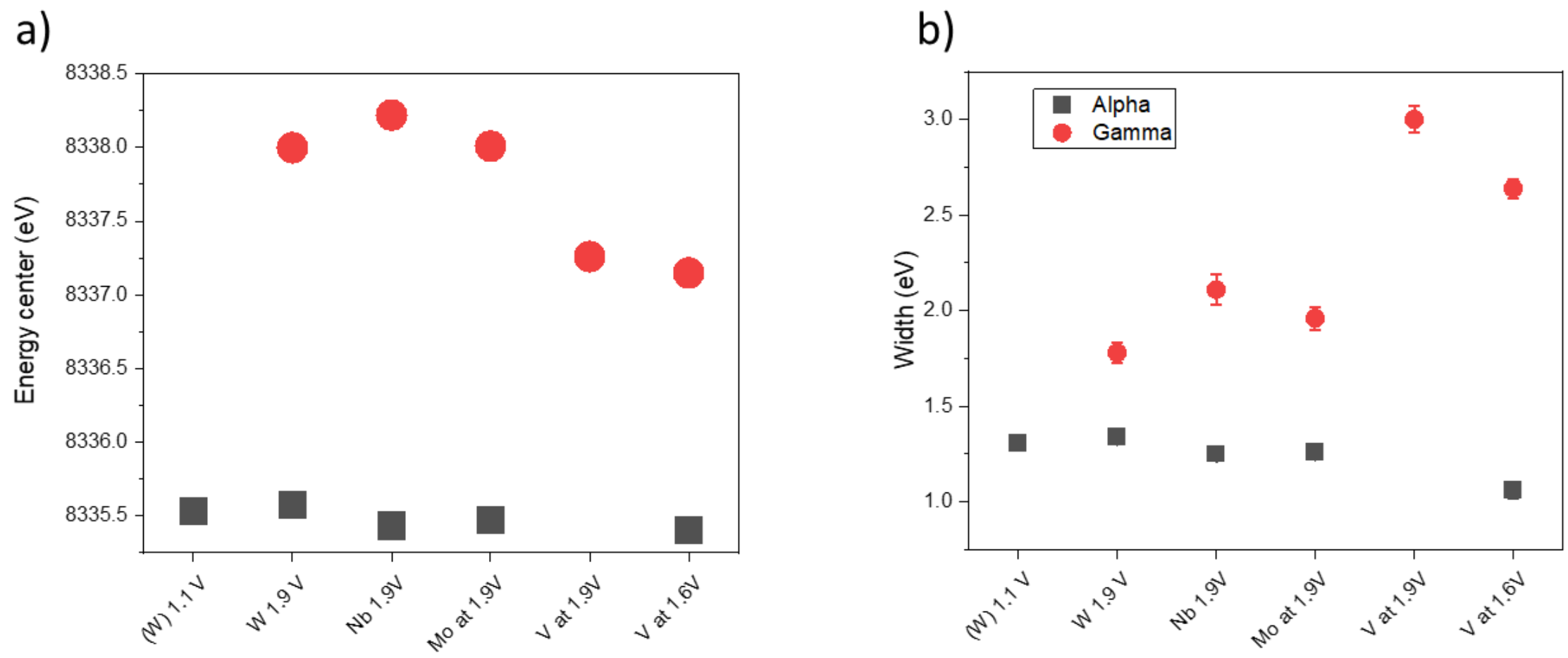

Supplementary Figure 17. Analysis of pre-peak regions from in situ HERFD-XAS. a) Energy centre and **b)** peak width of the pre-edge peaks associated with the inactive (alpha) and activated (gamma) phases from gaussian peak fit. The measured NiFeX LDH and the applied potential are shown in the x-axis (the metal X is used for indicating the corresponding LDH). For the potential of 1.1 V, only the peak for NiFeW LDH was fitted, as a reference, since the pre-peaks of all NiFeX LDH overlap (see *Figure 4b*). Thus, the letter W of the corresponding label is within brackets, to indicate that the data is from NiFeW LDH, but valid for all X. NiFe LDH pre-peak is so broad at high potentials that its fitting was not possible and it is thus not shown. For NiFeV LDH, the results of the fit of the pre-peak at two different OER potentials are shown, i.e. at 1.6 V and 1.9 V. At 1.9 V, the remaining alpha peak is not visible anymore, indicating a high conversion percentage of alpha-NiFeV LDH into gamma. Thus, only one peak was needed in the fit and only one point is shown in the graph. This point corresponds to a gamma-phase, as can be seen from the comparison with the data at 1.6 V.

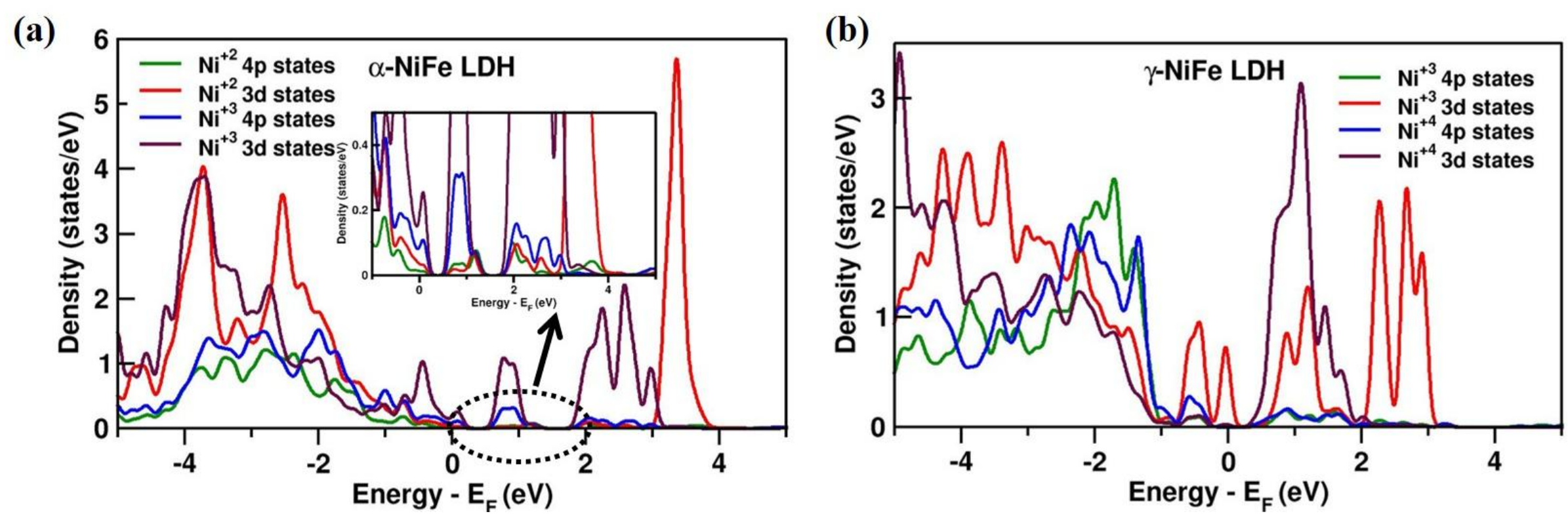

Supplementary Figure 18. Simulated Projected density of states (PDOS): Simulated Projected density of states (PDOS) of 3d and 4p orbitals of Ni atoms for **a)** α-phase with +2 and +3 oxidation states and **b)** γ-phase with +3 and +4 oxidation states. The inset in (a) is a magnification to better visualize the low intensity 4p states above the Fermi level. The 4p orbitals are enlarged by 10 times. The Fermi level is set at 0 eV.

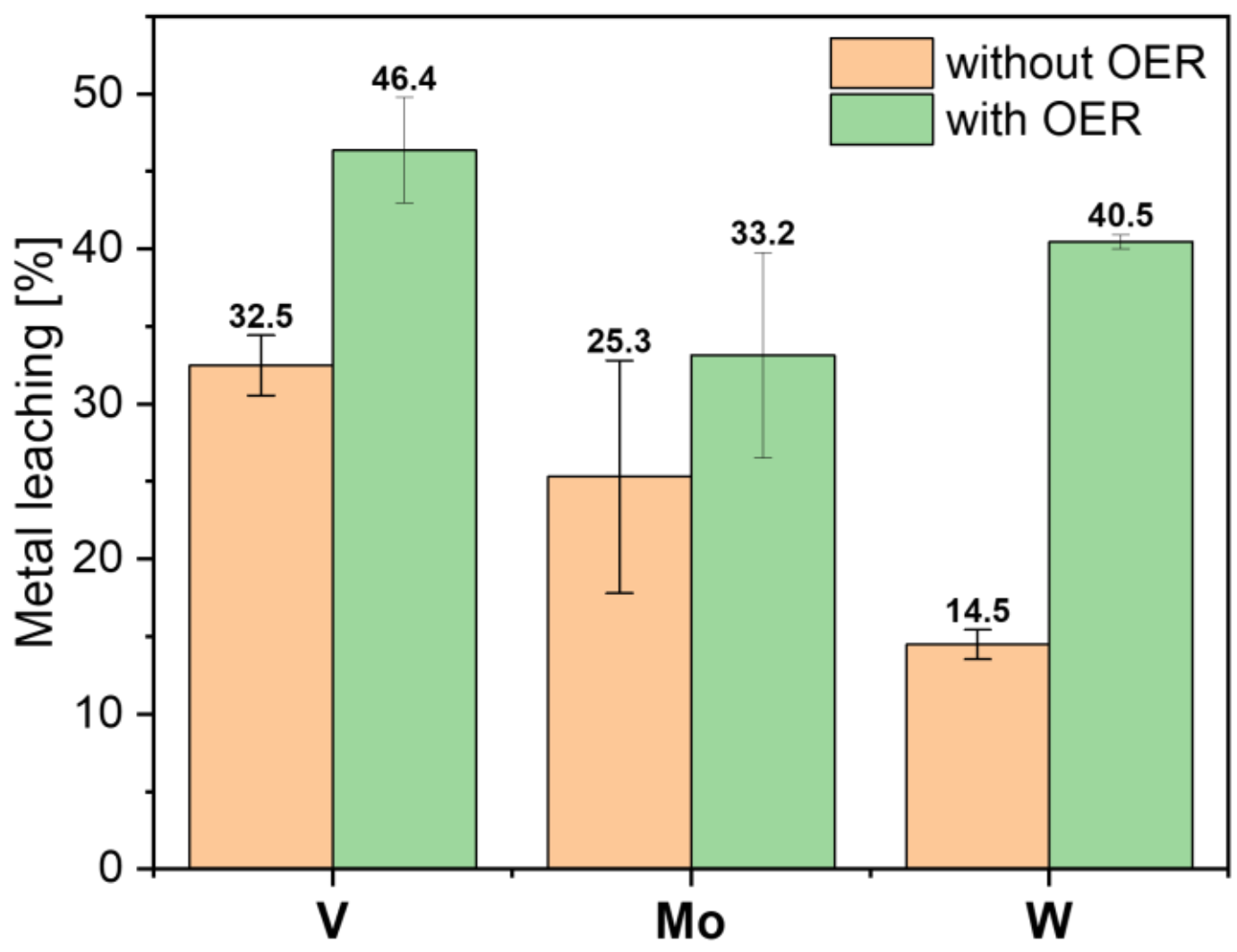

Supplementary Figure 19. Chemical Stability of selected NiFeX LDH Catalysts. Metal leaching percentages of NiFeV, NiFeMo and NiFeW LDHs with and without the standard electrochemical procedure described in *Supplementary Methods 3.4* applied. Measurements were taken before the experiment as well as after 60 minutes and calculated based on the mass of catalysts and the atomic ratios shown in *Supplementary Table 4.*

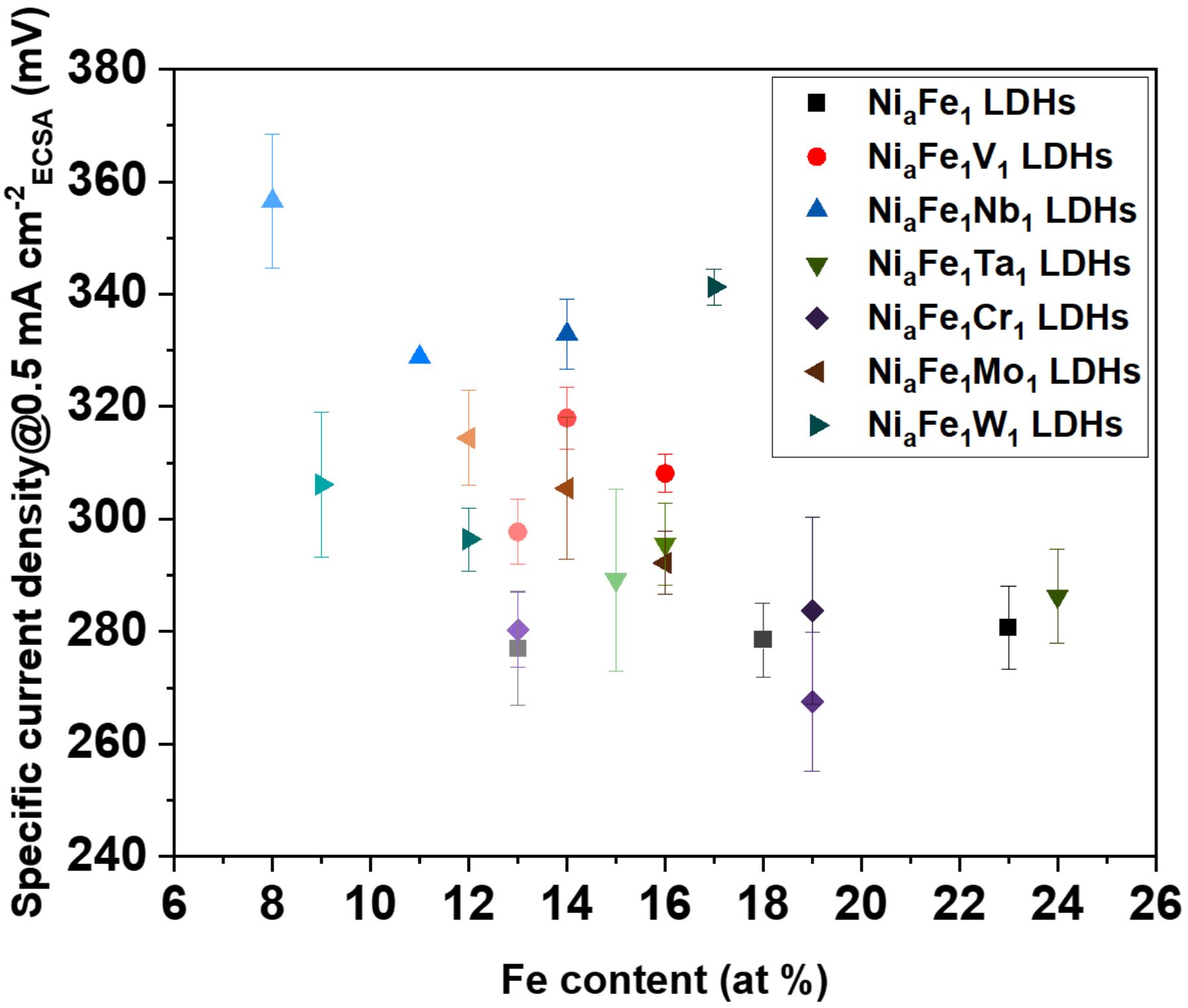

Supplementary Figure 20. Comparison of intrinsic activity as a function of Fe content (at%). The intrinsic activities were obtained by normalizing LSV curves to each ECSA value obtained by electrochemical impedance spectroscopy using the equivalent circuit described in *Supplementary Figure 10*. Fe content (at%) was obtained by ICP-OES (see *Supplementary Table 1*).

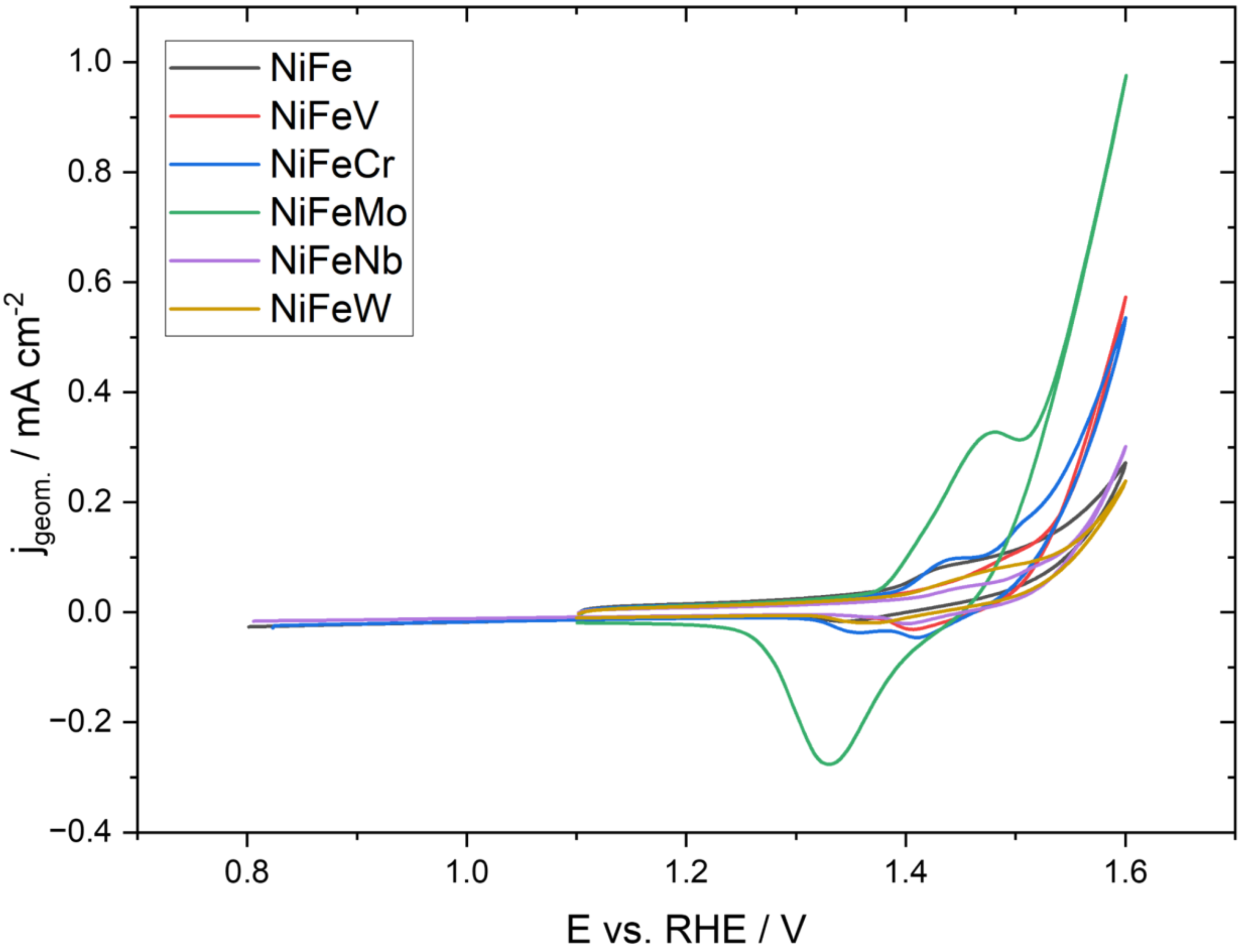

Supplementary Figure 21. Electrochemistry measured during XAS experiments. The graph shows the electrochemical performance before the XAS experiments in the cell used for the XAS experiments. CVs were performed to check if the catalyst was in contact with the electrolyte. The presence of a hysteresis or peak indicating oxidation/reduction of material was sufficient for us to start the XAS experiments. The actual electrochemistry during illumination by the beam is not shown here.

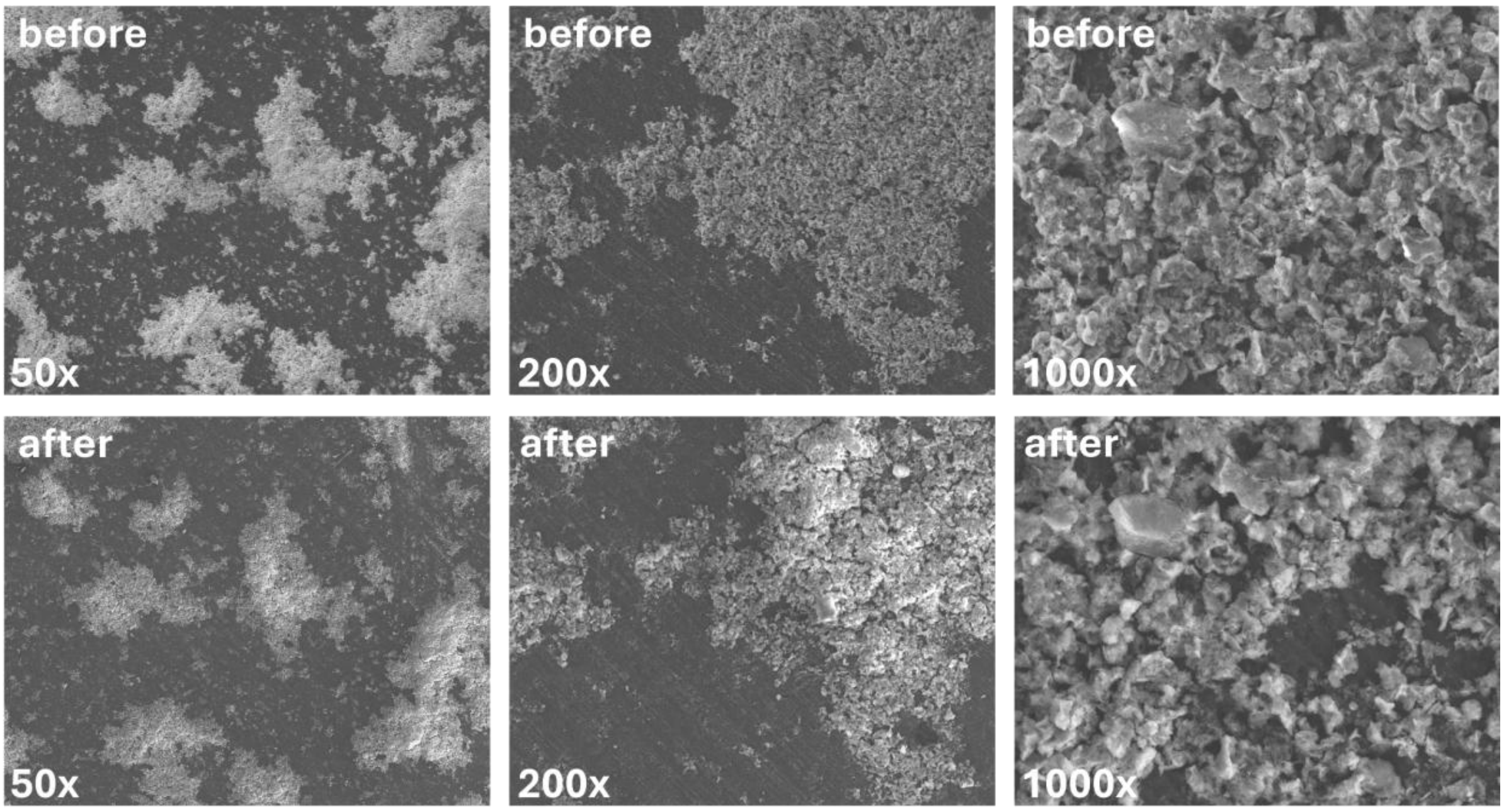

Supplementary Figure 22. SEM before and after RDE experiments of NiFe LDH coated glassy carbon electrode. Pictured is a glassy carbon electrode coated with NiFe LDH as described in Supplementary Methods 3.3. The catalyst loading was doubled compared to the performance tests. 50x, 200x and 1000x were chosen as magnification, with EDX being performed at the 1000x magnification. The same spot was found by previously marking the electrode and then combining triangulation and searching until the position was regained.

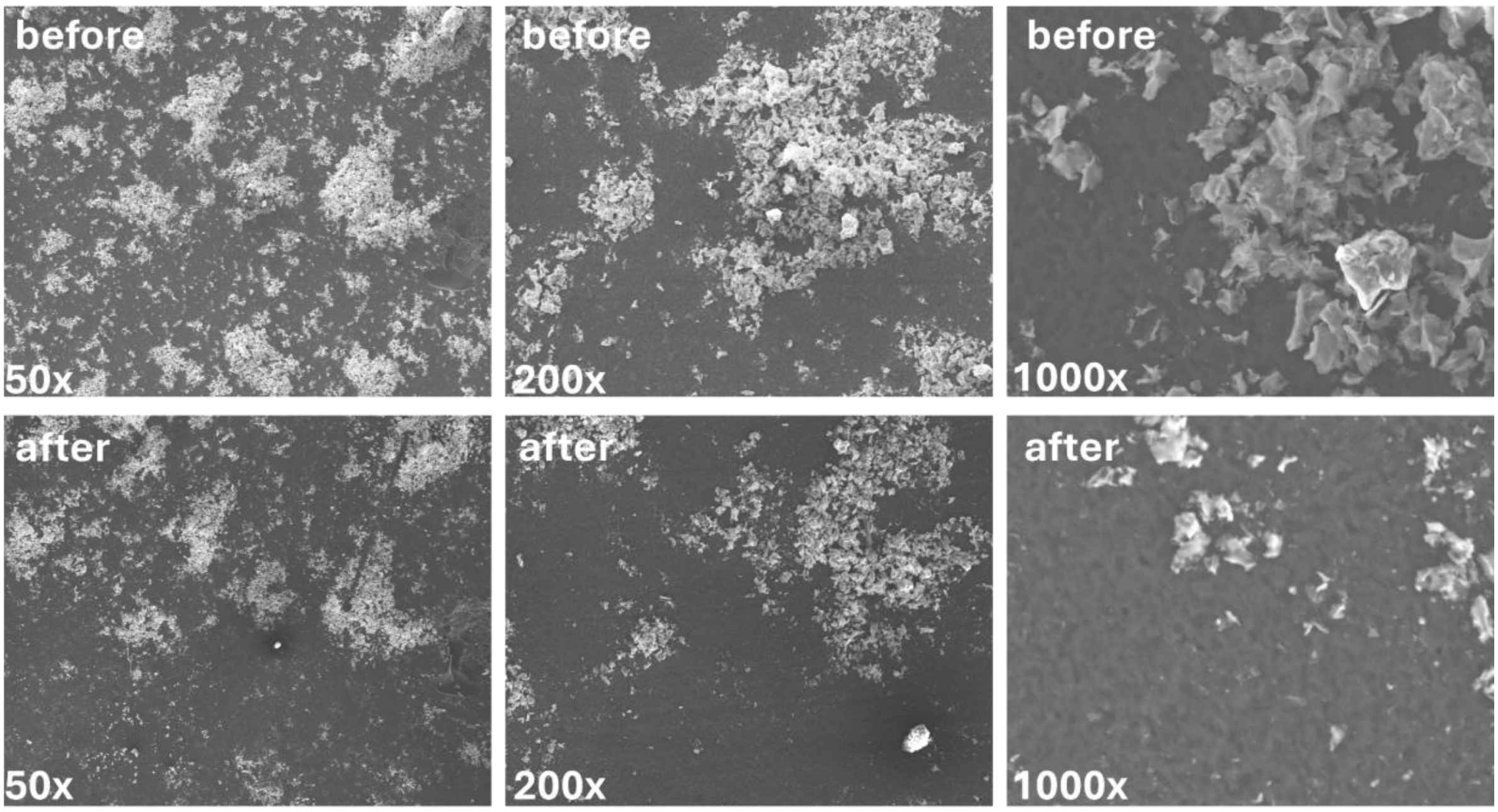

Supplementary Figure 23. SEM before and after RDE experiments of NiFeCr LDH coated glassy carbon electrode. Pictured is a glassy carbon electrode coated with NiFeCr LDH as described in Supplementary Methods 3.3. The catalyst loading was doubled compared to the performance tests. 50x, 200x and 1000x were chosen as magnification, with EDX being performed at the 1000x magnification. The same spot was found by previously marking the electrode and then combining triangulation and searching until the position was regained.

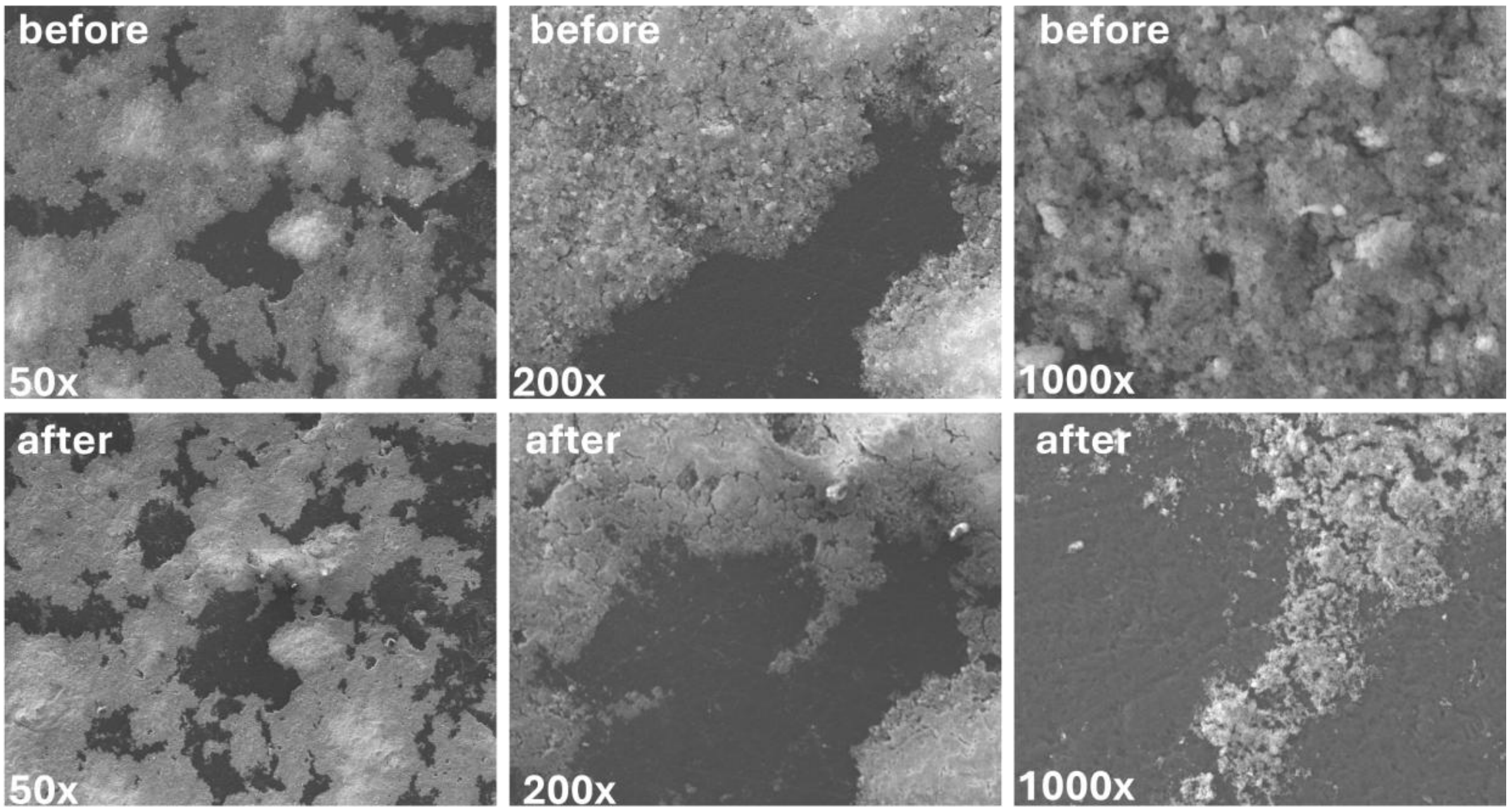

Supplementary Figure 24. SEM before and after RDE experiments of NiFeMo LDH coated glassy carbon electrode. Pictured is a glassy carbon electrode coated with NiFeMo LDH as described in Supplementary Methods 3.3. The catalyst loading was doubled compared to the performance tests. 50x, 200x and 1000x were chosen as magnification, with EDX being performed at the 1000x magnification. The same spot was found by previously marking the electrode and then combining triangulation and searching until the position was regained.

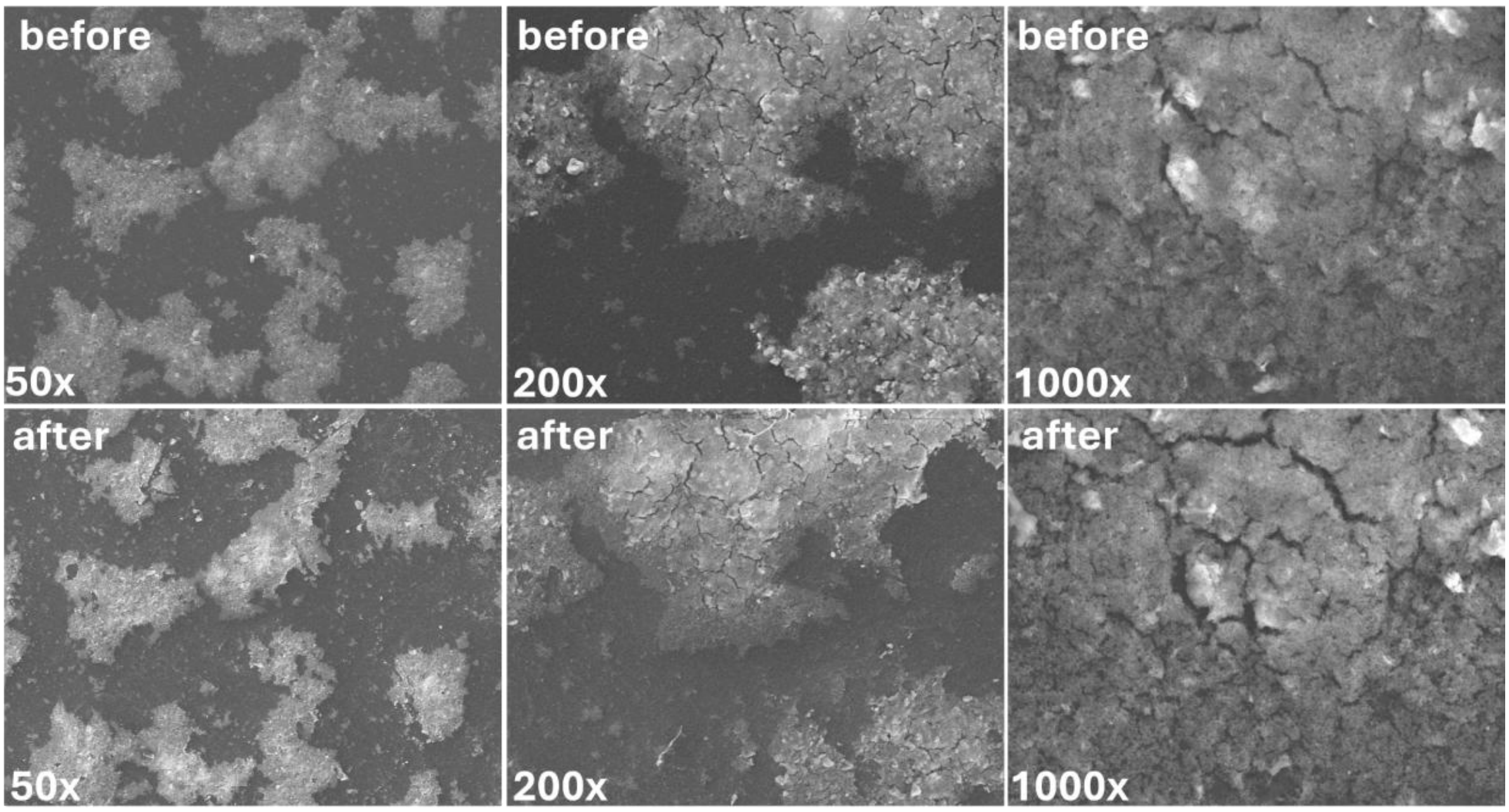

Supplementary Figure 25. SEM before and after RDE experiments of NiFeV LDH coated glassy carbon electrode. Pictured is a glassy carbon electrode coated with NiFeV LDH as described in Supplementary Methods 3.3. The catalyst loading was doubled compared to the performance tests. 50x, 200x and 1000x were chosen as magnification, with EDX being performed at the 1000x magnification. The same spot was found by previously marking the electrode and then combining triangulation and searching until the position was regained.

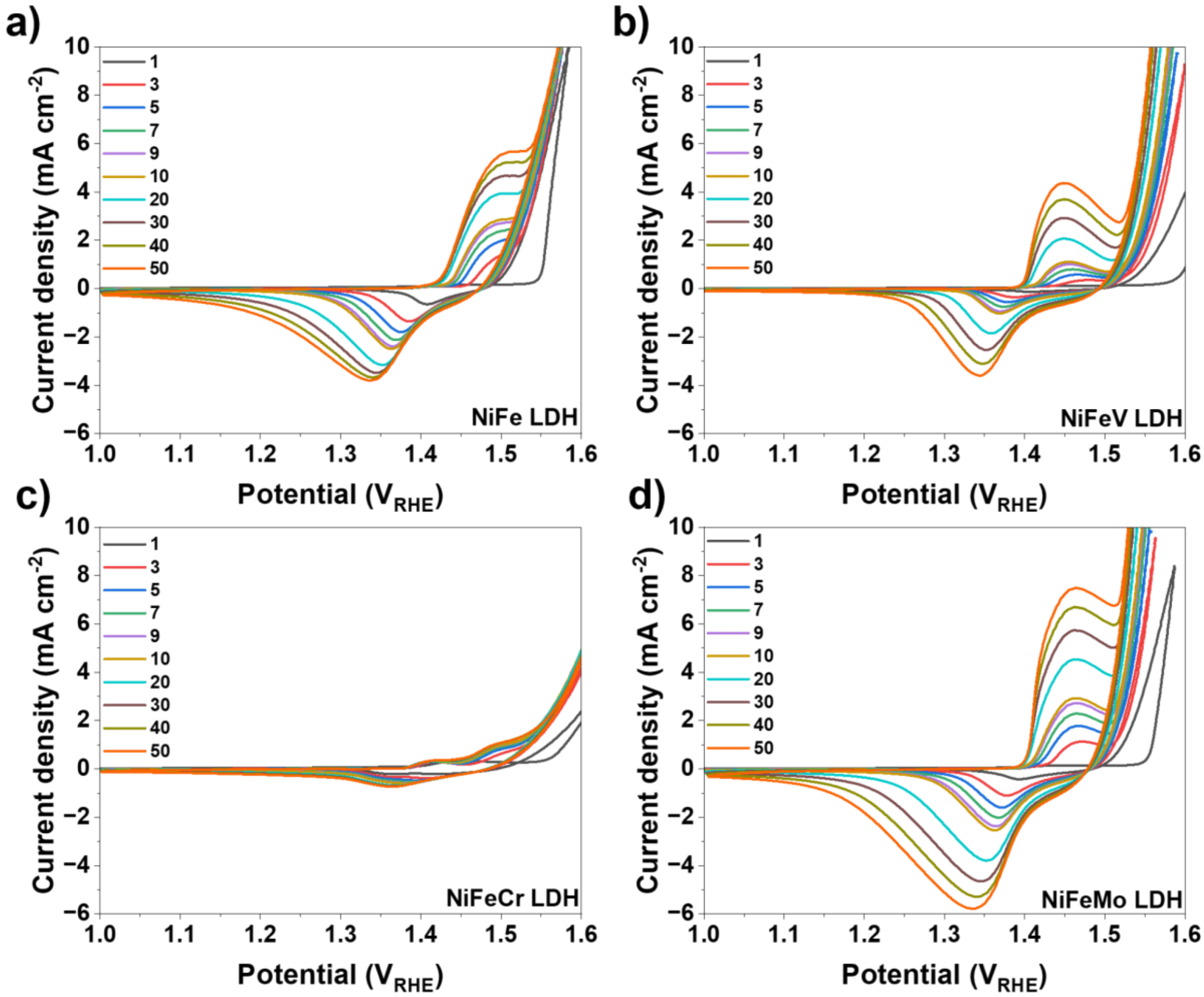

Supplementary Figure 26. Evolution of cyclic voltammetric curves (CVs, 50 mV/s) of selected NiFeX LDH catalyst used for post-mortem morphology analysis, recorded during RDE measurements (in 0.1 M KOH, 1600 rpm). a) NiFe LDH, **b)** NiFeV LDH, **c)** NiFeCr LDH, and **d)** NiFeMo LDH.

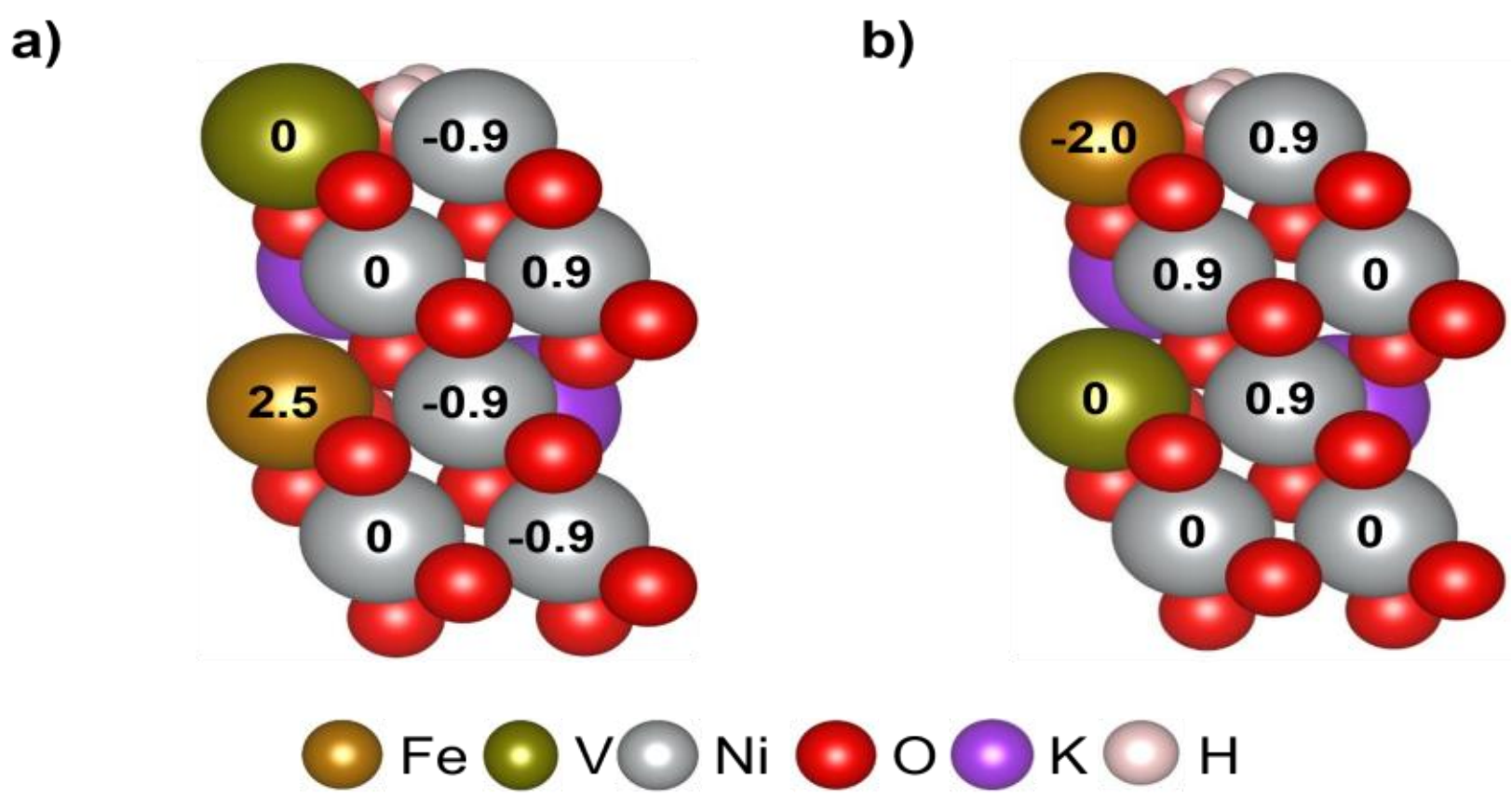

Supplementary Figure 27. Front view of the two representative V dopant configurations in the ternary γ-NiFeV LDH saturated with intercalated K^+ cations and water molecules. The bulk configuration as in **a)** shows a calculated energy difference of 26 meV/$Ni_6FeVK_2O_{16}.4H_2O$ as compared to **b)**, indicating they are nearly degenerate. The local magnetic moments are given for each metal atom.

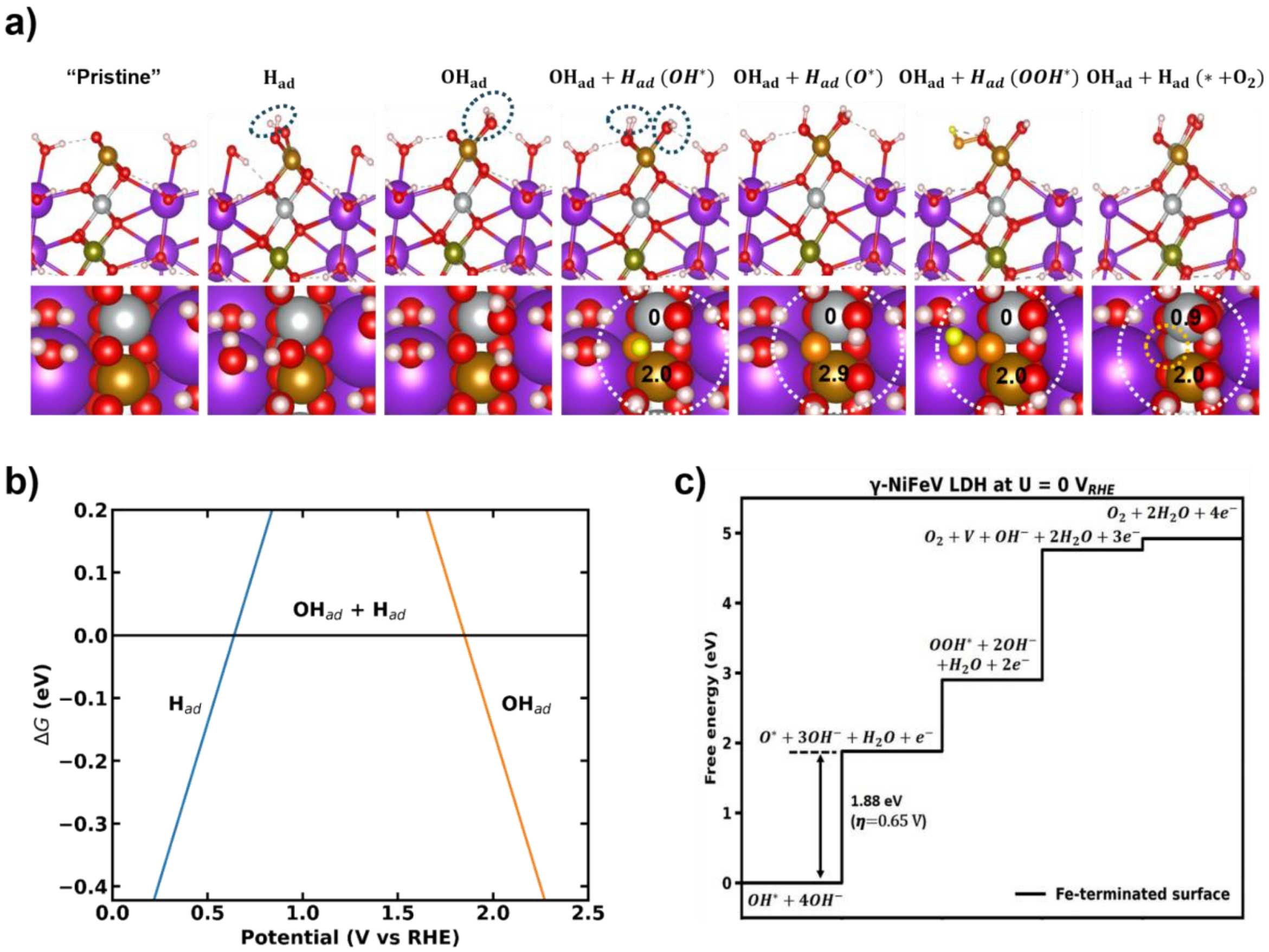

Supplementary Figure 28. OER mechanism on the Fe-terminated γ-NiFeV LDH surface. a) structures of different phases and OER intermediates; adsorbates of surface phases are highlighted by blue circles on the side views, and OER intermediates are differentiated by colors (yellow instead of white for hydrogen and orange instead of red for oxygen, respectively). A dashed orange circle indicates the formation of a surface O vacancy. The reaction centers are highlighted by large white dotted circles. The magnetic moments of Ni and Fe during the OER are also indicated on the top views. **b)** Surface phase diagram of Fe-terminated γ-NiFeV LDH surface. All the representative surface phases are given in **a)**. **c)** Reaction free-energy diagrams of OER on the Fe-terminated surface of γ-NiFeV; the potential limiting steps and the overpotentials are also given.

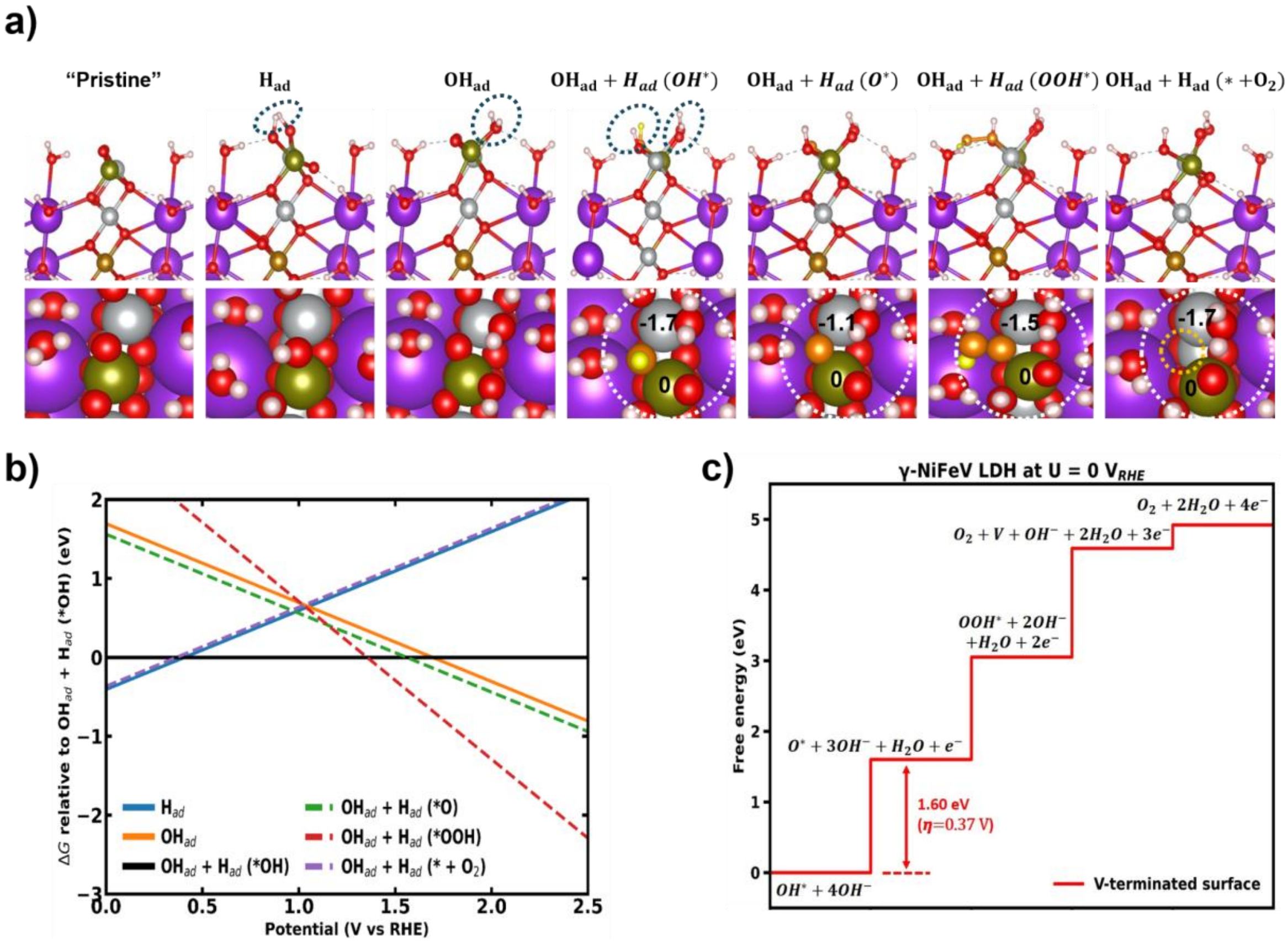

Supplementary Figure 29. OER mechanism on the V-terminated γ-NiFeV LDH surface. a) structures of different surface phases and OER intermediates; adsorbates of surface phases are highlighted by blue circles on the side views, and OER intermediates are differentiated by colors (yellow instead of white for hydrogen and orange instead of red for oxygen, respectively). A dashed orange circle indicates the formation of a surface O vacancy. The reaction centers are also indicated on the top views. **b)** Surface phase diagram of V-terminated γ-NiFeV LDH Surface. All the representative surface phases are given in **a)**. **c)** Reaction free-energy diagrams of OER on the V-terminated surface of γ-NiFeV; the potential limiting steps and the overpotentials are also given.

Supplementary Tables

Supplementary Table 1. **Summary of metal content obtained from ICP-OES.** All samples investigated via ICP-OES are as-synthesized. For calculating metal atomic content, Ni content and X content were normalized by Fe content.

Samples	Metal atomic ratio (starting)	Metal atomic ratio (final)	Metal atomic ratio (at%), (final)
**Ni_5Fe_1 LDH**	5:1	3.2:1	76:23
**$Ni_{7.5}Fe_1$ LDH**	7.5:1	4.4:1	82:18
**$Ni_{10}Fe_1$ LDH**	10:1	6.4:1	87:13
**$Ni_5Fe_1V_1$ LDH**	5:1:1	4:1:1.1	66:16:18
**$Ni_{7.5}Fe_1V_1$ LDH**	7.5:1:1	5.1:1:1	71:14:15
**$Ni_{10}Fe_1V_1$ LDH**	10:1:1	6:1:1.1	74:13:13
**$Ni_5Fe_1Nb_1$ LDH**	5:1:1	5.2:1:0.8	75:14:11
**$Ni_{7.5}Fe_1Nb_1$ LDH**	7.5:1:1	7.5:1:0.7	81:11:8
**$Ni_{10}Fe_1Nb_1$ LDH**	10:1:1	10.1:1:0.7	86:8:6
**$Ni_5Fe_1Ta_1$ LDH**	5:1:1	2.8:1:0.4	68:24:8
**$Ni_{7.5}Fe_1Ta_1$ LDH**	7.5:1:1	5:1:0.25	80:16:4
**$Ni_{10}Fe_1Ta_1$ LDH**	10:1:1	5.4:1:0.2	82:15:3
**$Ni_5Fe_1Cr_1$ LDH**	5:1:1	3.4:1:0.9	64:19:17
**$Ni_{7.5}Fe_1Cr_1$ LDH**	7.5:1:1	3.4:1:1	64:19:17
**$Ni_{10}Fe_1Cr_1$ LDH**	10:1:1	5.9:1:1	75:13:12
**$Ni_5Fe_1Mo_1$ LDH**	5:1:1	4.2:1:1.1	66:16:18
**$Ni_{7.5}Fe_1Mo_1$ LDH**	7.5:1:1	5.0:1:1.1	71:14:15
**$Ni_{10}Fe_1Mo_1$ LDH**	10:1:1	6.3:1:0.9	77:12:11
**$Ni_5Fe_1W_1$ LDH**	5:1:1	4.4:1:0.6	73:17:10
**$Ni_{7.5}Fe_1W_1$ LDH**	7.5:1:1	6.4:1:0.8	78:12:10
**$Ni_{10}Fe_1W_1$ LDH**	10:1:1	9.4:1:1	82:9:9

Supplementary Table 2. Summary of X precursors, synthesis methods, and thermal treatments for the synthesis of catalysts investigated in this study. Two-step synthesis consists of co-precipitation and solvothermal synthesis (see *Supplementary Methods* section *1.3*). ST = Standard thermal treatment (see *Supplementary Methods* section *1.1*). MT = Modified thermal treatment including pre-heating step (see *Supplementary Methods* section *1.6*)

Samples	X precursor	Synthesis method	Thermal treatment
**Ni_aFe_1 LDH**	·	Solvothermal synthesis	ST
**$Ni_aFe_1V_1$ LDH**	$V(acac)_3$	Solvothermal synthesis	ST
**$Ni_aFe_1Nb_1$ LDH**	$NH_4Nb(C_2O_4)_3$	Two-step synthesis	ST
**$Ni_aFe_1Ta_1$ LDH**	$TaCl_5$	Solvothermal synthesis	ST
**$Ni_aFe_1Cr_1$ LDH**	$Cr(NO_3)\cdot 9H_2O$	Solvothermal synthesis	ST
**$Ni_aFe_1Mo_1$ LDH**	$Mo(CO)_6$	Solvothermal synthesis	MT
**$Ni_aFe_1W_1$ LDH**	$W(CO)_6$	Solvothermal synthesis	MT
**$Ni_{10}Fe_1$ LDH_{MT}**	·	Solvothermal synthesis	MT
**$Ni_{7.5}Fe_1Cr_1$ LDH^{CO}_{MT}**	$Cr(CO)_6$	Solvothermal synthesis	MT

Supplementary Table 3. Summary of XPS results of 'X' metal. The XPS spectra were analyzed and charge corrected. The spectra were subsequently fitted (see *Supplementary Figure 6*) and the peak positions and proportions were extracted and compared to tabulated values.

Catalyst	Orbital	Peak 1 [eV]	Peak 2 [eV]	Oxidation State of 'X' and %
**NiFeV**	V 2p	516.1	523.7	IV
**NiFeCr**	Cr 2p	576.5	586.3	0 (8%); III (44%); VI (48 %)
**$NiFeCr^{CO}$**	Cr 2p	576.5	586.2	0 (1%); III (76%); VI (23%)
**NiFeNb**	Nb 3d	205.8	208.5	V
**NiFeMo**	Nb 3d	231.4	234.5	VI
**NiFeW**	W 4f	34.3	36.4	IV (84%); VI (16%)

Supplementary Table 4. Summary of metal atomic ratio (at%) obtained from ICP-OES, geometric overpotential (mV) at 10 mA/cm^2, and Intrinsic overpotential (mV) at 0.5 mA/cm$^2_{ECSA}$ of all samples investigated in this study. Linear sweep voltammetry (LSV) was performed at a scan rate of 1 mV/s in 0.1 M KOH (see detailed *Supplementary Methods* section *3*) by RDE (1600 rpm) and repeated at least 3 times to obtain statistical average and standard deviation. Catalyst loading on GC electrodes: 0.1 mg/cm^2.

Sample	Metal atomic ratio (at%), (final)	Geometric overpotential at 10 mA/cm^2 (mV)	Intrinsic overpotential at 0.5 mA/cm$^2_{ECSA}$ (mV)
**Ni_5Fe_1 LDH**	76:23	402±11	281±7
**$Ni_{7.5}Fe_1$ LDH**	82:18	357±3	278±7
**$Ni_{10}Fe_1$ LDH**	87:13	386±4	277±10
**$Ni_5Fe_1V_1$ LDH**	66:16:18	360±4	308±3
**$Ni_{7.5}Fe_1V_1$ LDH**	71:14:15	356±7	318±6
**$Ni_{10}Fe_1V_1$ LDH**	74:13:13	330±5	298±6
**$Ni_5Fe_1Nb_1$ LDH**	75:14:11	380±12	333±6
**$Ni_{7.5}Fe_1Nb_1$ LDH**	81:11:8	430±8	329±0.2
**$Ni_{10}Fe_1Nb_1$ LDH**	86:8:6	389±14	356±12
**$Ni_5Fe_1Ta_1$ LDH**	68:24:8	403±23	286±8
**$Ni_{7.5}Fe_1Ta_1$ LDH**	80:16:4	384±14	295±7
**$Ni_{10}Fe_1Ta_1$ LDH**	82:15:3	371±18	289±16
**$Ni_5Fe_1Cr_1$ LDH**	64:19:17	373±6	284±17
**$Ni_{7.5}Fe_1Cr_1$ LDH**	64:19:17	422±14	267±12
**$Ni_{10}Fe_1Cr_1$ LDH**	75:13:12	391±11	280±7
**$Ni_5Fe_1Mo_1$ LDH**	66:16:18	314±3	292±6
**$Ni_{7.5}Fe_1Mo_1$ LDH**	71:14:15	313±6	305±13
**$Ni_{10}Fe_1Mo_1$ LDH**	77:12:11	310±2	314±9
**$Ni_5Fe_1W_1$ LDH**	73:17:10	355±5	341±3
**$Ni_{7.5}Fe_1W_1$ LDH**	78:12:10	351±3	296±7
**$Ni_{10}Fe_1W_1$ LDH**	82:9:9	347±4	306±13

Supplementary Table 5. EDX derived chemical composition of selected catalyst layers before and after RDE experiments. The results are all normalized to the amount of Ni detected.

Sample	Ni	Fe	X
**NiFe LDH (before)**	1	0.18	
**NiFe LDH (after)**	1	0.17	
**NiFeCr (before)**	1	0.29	0.27
**NiFeCr (after)**	1	0.30	0.21
**NiFeMo* (before)**	1	0.13	0.10
**NiFeMo* (after)**	1	0.13	0.08
**NiFeV (before)**	1	0.11	0.14
**NiFeV (after)**	1	0.11	0.12

***** Note that the signal of Mo significantly overlaps with that of sulphur (S) present in the binder material

Supplementary Methods

1. Synthesis of Ni_aFe_1 LDH and $Ni_aFe_1X_1$ LDH (X = V, Nb, Ta, Cr, Mo, and W)

In this study, we followed the solvothermal method reported by Klingenhof et al.[1] and co-precipitation method depending on the third metal X. For the synthesis of $Ni_aFe_1X_1$ LDH, 0.6 M of metal precursor solutions (e.g $Ni(OAc)_2{\cdot}4H_2O$ and $Fe(NO_3)_3{\cdot}9H_2O$ and the third metal X precursors) were employed. The volume of Ni metal precursor solution was fixed at 1200 μl. In contrast to Ni salt precursor solution, the volume of Fe and the third metal X precursor solutions varied depending on the desired starting molar ratios. The process of separation, washing and drying was completely the same irrespective of the third metal X. For example, after thermal treatment, the solution was centrifuged at 8500 rpm for 15 mins to collect the precipitate. Subsequently, the collected precipitate was centrifuged with 10 ml of Milli-Q water at 8500 rpm for 15 mins, with 10 ml of absolute ethanol at 8500 rpm for 15 mins and 10 ml of Milli-Q water at 8500 rpm for 15 mins to eliminate the residue and organic solvent. Finally, the washed precipitate was dried in a freeze dryer.

1.1 Ni_aFe_1 LDH

The metal salt precursor solutions were mixed with a certain volume, corresponding to the desired starting molar ratios. (e.g. 1200 μl of Ni salt precursor solution and 240 μl of Fe salt precursor solution for Ni_5Fe LDH, 1200 μl of Ni salt precursor solution and 160 μl of Fe salt precursor solution for $Ni_{7.5}Fe$ LDH, 1200 μl of Ni salt precursor solution and 120 μl of Fe salt precursor solution for $Ni_{10}Fe$ LDH). The mixed solutions were added to 6 ml of DMF and stirred at 600 rpm overnight. After stirring, 4 ml of DMF and 8 ml of Milli-Q water were added and heated at a stirring rate of 1200 rpm via "ST" (standard thermal treatment). (e.g. 120 ℃ for 60 mins and 160 ℃ for 30 mins, successively.) After thermal treatment, the precipitates were collected, washed and dried as described in section 1.

1.2 $Ni_aFe_1V_1$ LDH

For the synthesis of $Ni_aFe_1V_1$ LDH, $V(acac)_3$ was employed as V metal salt precursor. 0.6 M of $V(acac)_3$ solution was prepared by dissolving it in DMF. The rest of the procedures was the same as described in section 1.1.

1.3 $Ni_aFe_1Nb_1$ LDH

For the synthesis of $Ni_aFe_1Nb_1$ LDH, $NH_4Nb(C_2O_4)_3$ was employed as Nb metal salt precursor. 0.6 M of $NH_4Nb(C_2O_4)_3$ was prepared by dissolving it in DMF. $Ni_aFe_1Nb_1$ LDH were synthesized via two-step synthesis consisting of co-precipitation and solvothermal synthesis. Employing a two-step synthesis method, the desired starting molar ratios were obtained by mixing the metal salt precursor solutions as described in section 1.1. The mixed solutions were added to 6 ml of 1.0 M of Na_2CO_3 aqueous solution and subsequently stirred at 600 rpm overnight. After stirring, 4 ml of DMF and 8 ml of Milli-Q water were added. The rest of the procedures was the same as described in section 1.1.

1.4 $Ni_aFe_1Ta_1$ LDH

For the synthesis of $Ni_aFe_1Ta_1$ LDH, $TaCl_5$ was employed as Ta metal salt precursor. 0.6 M of $TaCl_5$ was prepared by dissolving it in DMF. The rest of the procedures was the same as described in section 1.1.

1.5 $Ni_aFe_1Cr_1$ LDH

For the synthesis of $Ni_aFe_1Cr_1$ LDH, $Cr(NO_3)_3{\cdot}9H_2O$ was employed as Cr metal salt precursor. 0.6 M of $Cr(NO_3)_3{\cdot}9H_2O$ was prepared by dissolving it in DMF. The rest of procedures was the same as described in section 1.1.

1.6 $Ni_aFe_1Mo_1$ LDH

For the synthesis of $Ni_aFe_1Mo_1$ LDH, $Mo(CO)_6$ was employed as Mo metal salt precursor. The metal salt precursor solutions and $Mo(CO)_6$ metal salt precursor were mixed corresponding to the desired starting molar ratios. (e.g. 1200 µl of Ni salt precursor solution, 240 µl of Fe salt precursor solution, 38.0 mg of powder $Mo(CO)_6$, and 240 µl of DMF for Ni_5FeMo LDH, 1200 µl of Ni salt precursor solution, 160 µl of Fe salt precursor solution, 25.3 mg of powder $Mo(CO)_6$, and 160 µl of DMF for $Ni_{7.5}FeMo$ LDH, and 1200 µl of Ni salt precursor solution, 120 µl of Fe salt precursor solution, 19.0 mg of powder $Mo(CO)_6$, and 120 µl of DMF for $Ni_{10}FeMo$ LDH). The mixed solutions were added to 10 ml of DMF and 8 ml of Milli-Q water. After adding, the solutions were heated at a stirring rate of 1200 rpm via "MT" (modified thermal treatment including pre-heating step at 50 °C for 15 mins). (e.g. at 50 °C for 15 mins, at 120 °C for 60 mins, and 160 °C for 30 mins, successively.) The procedure of separation, washing and drying was consistent with section 1.

1.7 $Ni_aFe_1W_1$ LDH

For the synthesis of $Ni_aFe_1W_1$ LDH, $W(CO)_6$ was employed as W metal salt precursor. The metal salt precursor solutions and $W(CO)_6$ metal salt precursor were mixed corresponding to the desired starting molar ratios. (e.g. 1200 µl of Ni salt precursor solution, 240 µl of Fe salt precursor solution, 50.7 mg of powder $W(CO)_6$, and 240 µl of DMF for Ni_5FeW LDH, 1200 µl of Ni salt precursor solution, 160 µl of Fe salt precursor solution, 33.8 mg of powder $W(CO)_6$, and 160 µl of DMF for $Ni_{7.5}FeW$ LDH, and 1200 µl of Ni salt precursor solution, 120 µl of Fe salt precursor solution, 25.3 mg of powder $W(CO)_6$, and 120 µl of DMF for $Ni_{10}FeW$ LDH). The mixed solutions were added to 10 ml of DMF and 8 ml of Milli-Q water. The solutions were heated as described in section 1.6. The procedure of separation, washing and drying was consistent with section 1.

1.8 $Ni_{10}Fe_1$ LDH_{MT}

1200 µl of Ni salt precursor solution and 120 µl of Fe salt precursor solution were mixed. The mixed solution was added to 10 ml of DMF and 8 ml of Milli-Q water. After adding, the solution was heated as described in section 1.6. The procedure of separation, washing, and drying was consistent with section 1.

1.9 $Ni_{7.5}Fe_1Cr_1$ LDH^{CO}_{MT}

For the synthesis of $Ni_{7.5}FeCr$ LDH, $Cr(CO)_6$ was employed as Cr metal salt precursor. 1200 µl of Ni salt precursor solution, 160 µl of Fe salt precursor solution, 21.1 mg of powder $Cr(CO)_6$ metal salt precursor solution, and 160 µl of DMF were mixed. The mixed solution was added to 10 ml of DMF and 8 ml of Milli-Q water. After adding, the solution was heated as described in section 1.6. The procedure of separation, washing, and drying was consistent with section 1.

2. Physico-chemical Characterization

2.1 X-Ray-Diffractometry

XRD patterns were obtained by X-ray diffractometer (D8 Advance; Brucker) and X-ray tube (KFL CU 2K). A small amount of powder sample was loaded on the silicon wafer. The loaded powder samples were measured in the 2θ range 8° ~ 80° with a step size of 0.04° and 6 s measurement time for each step. An atomic emission spectrometer (715-ES; Varian) was employed for the ICP-OES measurement to investigate metal contents. About 1 mg of a powder sample was dissolved in 10 ml of acid consisting of 2 ml of sulfuric acid, 2 ml of nitric acid, and 6 ml of hydrogen chloride and subsequently heated 180 °C for 20 mins. The samples are diluted by adding Milli-Q water until 50 ml. 5 ml of diluted solution was taken and diluted with 5 ml of Milli-Q water. For the calibration curves, the standard solutions, which have different concentrations such as 1 mg/l, 3 mg/l, 5 mg/l, 7 mg/l, and 9 mg/l were prepared. To determine the concentrations of metal, the intensity at 230.299 nm for Ni, at 238.204 nm for Fe, at 311.837 nm for V, at 313.078 nm for Nb, at 263.558 nm for Ta, at 283.563 nm for Cr, at 281.615 nm for Mo, and at 207.912 nm for W was measured. TEM images were obtained using Tecnai G2 20 s-Twin, equipped with a LaB_6 cathode and a GATAN MS794 P CCD-detector at ZELMI Centrum, Technical

university of Berlin. A small amount of powder sample was dispersed in the mixture of isopropanol and Milli-Q water with a ratio 4:1 and 10 µl of dispersed solution was loaded on the Cu mesh (400 Mesh) and dried at 60 ℃ for 10 mins.

2.2 Same-spot Scanning Electron Microscopy

Following catalyst application, all disc electrodes were marked to enable relocation of specific surface regions for pre- and post-electrochemical comparison. Prior to any electrochemical treatment, scanning electron microscopy (SEM) was performed on all electrodes at magnifications of 50x, 200x, 1000x, and 20,000x, accompanied by energy-dispersive X-ray spectroscopy (EDX) at 1000x magnification to characterize the elemental composition of the catalyst layer. Oxygen evolution reaction (OER) measurements were subsequently carried out using a rotating disk electrode (RDE) setup following the standard protocol (*see* Supplementary Methods 3.4). After electrochemical testing, the electrodes were dried, rinsed with MilliQ water, and dried again before post-experiment characterization.

SEM and EDX analyses were repeated under identical conditions — the same magnification series and spectroscopy parameters — to assess any morphological or compositional changes induced by the electrochemical treatment. The same surface regions imaged prior to the experiment were relocated using a combination of triangulation from the fiducial marks and systematic visual searching, enabling direct comparison of pre- and post-OER electrode surfaces.

3. Electrochemical Characterization

3.1 Experimental Setup

A rotating disc electrode was employed with a three-electrode setup for electrochemical characterization. A glassy carbon electrode (GC, diameter 5 mm) as working electrode coated with as-synthesized catalysts was mounted in a rotator. A platin mesh electrode was employed as a counter electrode. A reversible hydrogen electrode (RHE) connected by Luggin capillary was employed as a reference electrode. The three electrodes are immersed in an unpurified 0.1 M KOH electrolyte, which was prepared with Milli-Q water and high-purity KOH pellets (semiconductor grade, 99.99 % trace metal basis, Sigma Aldrich). The measurement was controlled using a potentiostat (SP-200, BioLogic) and analyzed using software (EC-Lab V11.10).

3.2 Preparation of Electrodes

A GC was polished on a nylon cloth with an alumina polish (particle size 1.0 µm) for 1 min and subsequently on a micro cloth with an alumina polish (particle size 0.05 µl). After polishing, the GC electrode was sonicated with 5 ml of Milli-Q water, 5 ml of Isopropanol, and 5 ml of Milli-Q water, respectively, for 5 mins to eliminate the residue and subsequently dried with nitrogen gas flow.

3.3 Preparation of Ink

An Ink was prepared to coat the GC electrode. 1.97 mg of as-synthesized powder catalyst was added to the ink solution consisting of 800 µl of Isopropanol, 200 µl of Milli-Q water, and 5 µl of Nafion (Perfluorinated resin solution containing Nafion™ 1100W, 5 wt%, Sigma Aldrich). The ink solution was sonicated with a horn sonifier for 20 mins, so that the powder catalyst is homogeneous dispersed in the ink solution. 10 µl of ink solution was loaded on the GC electrode by drop casting and dried in an oven at 50 ℃ for 7 mins. The resulting loading on the GC electrode was 0.1 mg/cm^2.

3.4 Electrochemical Measurement Protocol

We followed the electrochemical measurement protocol reported by Dionigi et al.[2] An electrochemical measurement glass cell was filled with 0.1 M KOH, which was prepared with KOH pellets (semiconductor grade, 99.99 % trace metal basis, Sigma Aldrich). It was purged with nitrogen gas flow to eliminate the bubbles and to

be saturated by nitrogen gas. After purging, The GC electrode coated with catalyst was put in the electrolyte and rotated at rotating rate of 1600 rpm. After 3 minutes to stabilize the system, the measurement started as elucidated below. The steps from 1 to 6 were performed to investigate surface redox behaviors and the geometric overpotential at 10 mA/cm^2 of the catalysts. The step 7 and 8 were performed to obtain the Nyquist plot for the evaluation of ECSA values at 1.60 V_{RHE} selected by the fact, that OER adsorbates capacitance is saturated for Ni-based oxyhydroxide (e.g. NiFe LDH).[3]

1. OCP for 1 min: to evaluate the equilibrium potential
2. CA (E = OCP) for 1 min: to stabilize the current
3. PEIS (E = OCP, ν_1 = 100 kHz, ν_2 = 1.0 Hz, 10 points per decade): to evaluate the uncompensated resistance (R_u) caused by the solution
4. CV (E_1 = 1.0 V_{RHE}, E_2 = 1.75 V_{RHE}, ν = 50 mV/s, n = 50): to active the catalyst from α- (as synthesized phase) to γ-phase (active phase)
5. LSV (E_1 = 1.0 V_{RHE}, E_2 = 1.75 V_{RHE}, ν = 1 mV/s): to evaluate the geometric overpotential at 10 mA/cm^2
6. PEIS (E = OCP, ν_1 = 100 kHz, ν_2 = 1.0 Hz, 10 points per decade): to double check the uncompensated resistance (R_u)
7. CA (E = 1.60 V_{RHE}) for 1 min to stabilize the system for step 8
8. PEIS (E = 1.60 V_{RHE}, ν_1 = 100 kHz, ν_2 = 0.2 Hz, 10 points per decade): to obtain the Nyquist plot for evaluating OER adsorbates capacitance (C_a)

where,

E_1 = the starting potential
E_2 = the terminal potential
ν_1 = the highest frequency
ν_2 = the lowest frequency
ν = scan rate
n = the number of cycles

4. AEMWE Single-cell Measurement

4.1 Experimental Setup

All AEMWE single-cell measurements were performed using an AEM-Direkt consortium electrolyzer cell with an active area of 25 cm².[4] The cell was operated in a recirculating electrolyte configuration with separate anode and cathode electrolyte loops. Each loop consisted of a 700 ml reservoir, a peristaltic pump, a pulsation damper, and the electrolysis cell. The reservoirs additionally served as gas–liquid separators.

The electrolyte was 1.0 M KOH prepared from Milli-Q water and potassium hydroxide pellets (≥85%, EMSURE®, Merck). Electrolyte was circulated through both compartments at a flow rate of 100 ml/min. Pulsation dampers were installed upstream of the anode and cathode inlets to minimize flow fluctuations induced by the peristaltic pumps.

Both the electrolyte and cell hardware were maintained at 70 °C throughout operation. To ensure consistent testing conditions, the same cell hardware was used for all catalyst systems. Between measurements, the bipolar plates (BPPs) were regenerated by immersion in a 100 g/l oxalic acid solution at 60 °C for 7 min, followed by rinsing with Milli-Q water and drying before reuse.

4.2 Preparation of Electrodes and Fabrication of MEA

Catalyst inks were prepared using the synthesized anode catalysts ($Ni_{7.5}Fe_1$ LDH, $Ni_{10}Fe_1V_1$ LDH, and $Ni_{10}Fe_1Mo_1$ LDH), a 5 wt% anion-conducting ionomer solution (DURAION®, Evonik), ethanol (EtOH, >99.8%, VWR Chemicals), and ultrapure water. A representative anode ink batch consisted of 200 mg catalyst powder,

1000 mg ionomer solution, 7.9 mg EtOH, and 6.2 mg ultrapure water. The catalyst powder was first dispersed in water using a high-shear disperser (Ultra-Turrax, IKA) at 4000 rpm for 5 min. Subsequently, the ionomer solution and EtOH were added, followed by an additional 5 min of high-shear mixing and 10 min of ultrasonic homogenization using a horn sonicator (SONOPULS, BANDELIN; MS73 sonotrode, 50% amplitude) under continuous ice-bath cooling.

Cathode catalyst inks were prepared following the same procedure using commercial Pt/C catalyst powder (60 wt% Pt supported on high-surface-area carbon, Alfa Aesar). A representative cathode ink batch consisted of 250 mg Pt/C, 1333.3 mg ionomer solution, 20.3 mg EtOH, and 16.0 mg ultrapure water. The ionomer content was fixed at 20 wt% and 25 wt% of the total solids for anode and cathode inks, respectively.

Catalyst-coated substrates (CCSs) were fabricated by ultrasonic spray coating of the catalyst inks onto stainless-steel porous transport layers (PTLs, 800 µm thickness, Siemens Energy) using an ExactaCoat system (Sono-Tek) equipped with an AccuMist nozzle. The PTLs were mounted on a porous vacuum plate maintained at 80 °C and fixed using a stainless-steel mask defining an active area of 25 cm^2. The inks were supplied at a flow rate of 0.7 ml/min and deposited directly onto the PTLs in a layer-by-layer meander pattern. To improve coating uniformity, successive layers were sprayed in both x- and y-directions with alternating positive and negative offsets.

Catalyst loadings were controlled by the number of deposited layers and verified gravimetrically using reference substrates coated in parallel. The final anode catalyst loadings were 1.114, 1.110, and 1.084 mg_{cat}/cm^2 for $Ni_{7.5}Fe_1$ LDH, $Ni_{10}Fe_1V_1$ LDH, and $Ni_{10}Fe_1Mo_1$ LDH, respectively. The corresponding cathode Pt loadings were 0.248, 0.229, and 0.194 mg_{Pt}/cm^2. The resulting catalyst-coated substrates were dried under ambient conditions prior to assembly.

Membrane electrode assemblies (MEAs) were fabricated using a DURAION® anion-exchange membrane supplied by Evonik. The anode and cathode CCSs were positioned on either side of the membrane and assembled into the electrolyzer hardware for electrochemical characterization.

4.3 Single-cell Measurement Protocol

MEA pretreatment, cell conditioning, and polarization measurements were conducted according to the *EU harmonised polarization curve test method for low-temperature water electrolysis*.[5]

Prior to cell assembly, catalyst-coated substrates (CCSs) were immersed in 100 ml of 1.0 M KOH for 3 h at room temperature. To ensure complete ion exchange, the membrane was pretreated by immersion in two separate 1.0 M KOH solutions (~150 ml each) at 60 °C for 1 h each, with replacement of the KOH solution between treatments. The membrane was subsequently immersed in a fresh 1.0 M KOH solution at 60 °C for ~16 h. In total, the membrane underwent three consecutive immersions in 1.0 M KOH.

The cell was assembled using a torque of 2.5 N m. For all measurements, 1.0 M KOH was supplied to both the anode and cathode compartments, with electrolyte volumes of 700 mL per compartment. Electrolyte was circulated at 100 ml/min (4 $mL\ min^{-1}\ cm^{-2}$) unless otherwise noted. When required, thermal insulation was placed beneath the cell to minimize heat loss to the supporting surface.

Following cell assembly, the electrolyte was heated under open-circuit conditions until the inlet temperature reached 70 °C. The cell was subsequently conditioned using a galvanostatic current-density ramp from 0.01 to 1.0 A/cm^2 at a scan rate of 0.4 $mA/cm^2{\cdot}s$ with a current-density increments of 0.1 A/cm^2. After reaching 1.0 A/cm^2, the cell was operated galvanostatically at this current density for 1 h. Subsequently, galvanostatic electrochemical impedance spectroscopy (GEIS) measurements were performed at 1 A/cm^2. Impedance spectra were obtained over a frequency range of 300 kHz to 300 mHz using a current perturbation amplitude 2.5 A.

Prior to polarization measurements, the cell was equilibrated at 0.001 A/cm^2 for 30 min to establish thermal and electrochemical steady-state conditions. Polarization curves were subsequently recorded in 1.0 M KOH at 70 °C with an electrolyte flow rate of 100 ml/min on both the anode and cathode sides. Measurements were performed galvanostatically between 0.001 and 2.0 A/cm^2 using the current-density sequence specified in the *EU harmonised*

polarization curve test method for low-temperature water electrolysis.

5. HERFD-XAS Experiments at ESRF ID26

5.1 Sample Preparation

A catalyst ink was prepared as described in *Supplementary Methods Section 3.3* and subsequently 10 µL of these inks were dropcast on laser-engraved glassy carbon disks (d = 5 mm).

5.2 Sample Environment

0.1 M KOH was chosen as electrolyte, and a flow of 25 mL per minute through the in-situ cell depicted in *Supplementary Figure 1* was kept constant. The pressure inside the cell was controlled by shifting the vertical position of the electrolyte reservoir such that a globe of electrolyte was present above the sample.

5.3 Electrochemical Protocol

The electrochemical protocol was as follows:

1. OCV → skipped after stabilisation
2. PEIS (E = OCP, ν_1 = 5MkHz, ν_2 = 1.0 Hz, 6 points per decade)
3. CV (E_1 = OCP, E_2 = 1.6 V_{RHE}, E_3 = 1.1 V_{RHE}, ν = 20 mV/s, n = 2)
4. MP:
    a. E = 1.1 V_{RHE} → skipped after XAS finished
    b. Scan 1.1 V_{RHE} → 1.4 V_{RHE}, v = 5 mV/s
    c. E = 1.4 V_{RHE} → skipped after XAS finished
    d. Scan 1.4 V_{RHE} → 1.6 V_{RHE}, v = 5 mV/s
    e. E = 1.6 V_{RHE} → skipped after XAS finished
    f. Scan 1.6 V_{RHE} → 1.1 V_{RHE}, v = 5 mV/s
    g. E = 1.1 V_{RHE} → skipped after XAS finished
    h. Scan 1.1 V_{RHE} → 1.9 V_{RHE}, v = 5 mV/s
    i. E = 1.9 V_{RHE} → skipped after XAS finished

At each potential step a waiting time of a minimum of 15 min was observed or until the current signal had stabilized (whichever later).

5.4 HERFD Axis Calibration

Incident energy was selected using a cryogenically-cooled fixed-exit double-crystal monochromator including a flat Si[111] pair and a flat Si[311] pair with angular ranges between 5 and 60 degrees. A Rowland circle spectrometer with five spherically bent crystals was aligned to select the Ni Kα line.

6. Stability Measurements

Electrochemical stability measurements were carried out using a conventional three-electrode set-up. For this purpose, a working electrode (WE), a reference electrode (RE) and a counter electrode(CE) were placed in an electrochemical cell. A miniflex RHE-electrode was employed as the reference electrode. A platinum mesh served as the CE. For the stability measurements, a self-made PTFE cell was used. A glassy carbon electrode with a surface area of 0.7854 cm2 served as the WE. The rotation speed was set to 1600 rpm. Before each measurement, 30 mL of a 0.1 M KOH solution were added to the cell. Subsequently, 1 mL of the electrolyte was removed for testing. After the measurement, a further 1 mL of the electrolyte was sampled after one hour in order to allow subsequent analysis of the electrolyte by ICP-MS. To investigate the influence of the electrochemistry on the catalyst stability, additional experiments were performed in which the prepared electrode was immersed in the

electrolyte for one hour without application of potential.

Supplementary Discussion

1. Verifying the Origin of Large ECSA Values of $Ni_aFe_1Mo_1$ and $Ni_aFe_1W_1$ LDHs

Impact of preheating step

To verify and pin down the origin of the large ECSA values of $Ni_aFe_1Mo_1$ LDHs and $Ni_aFe_1W_1$ LDHs, first the impact of the preheating step at 50 °C for 15 min on the crystallinity, redox behaviors, and corresponding ECSA values was investigated. This was achieved by comparing the XRD patterns, CVs, and ECSA values of $Ni_{10}Fe_1$ LDH, $Ni_{10}Fe_1$ LDH_{MT}, and $Ni_{10}Fe_1W_1$ LDH (*Supplementary Figure 11*), where the $Ni_{10}Fe_1$ LDH_{MT} sample was synthesized following a modified thermal (MT) treatment, which introduced the preheating step also in the synthesis of binary NiFe LDH. *Supplementary Figure 11* confirms that the preheating step leads to lower crystallinity of $Ni_{10}Fe_1$ LDH_{MT} based on the broadening of (003) and (006) peaks. Interestingly, despite the lower crystallinity of $Ni_{10}Fe_1$ LDH_{MT} compared to $Ni_{10}Fe_1$ LDH, the two catalysts exhibited almost the same Ni oxidation peak areas and ECSA values.

Impact of CO ligands during synthesis

Subsequently, the impact of CO ligands was investigated in the same way by comparing $Ni_{7.5}Fe_1$ LDH and $Ni_{7.5}Fe_1Cr_1$ LDH (both synthesized without metal precursors that contained CO ligands) with $Ni_{7.5}Fe_1Cr_1$ LDH^{CO}_{MT} (containing CO ligands), as a carbonyl precursor was also available for Cr. As shown in *Supplementary Figure 12*, $Ni_{7.5}Fe_1Cr_1$ LDH^{CO}_{MT} synthesized by solvothermal synthesis with modified thermal treatment, including a preheating step with $Cr(CO)_6$, exhibited an open morphology, resulting in a large Ni oxidation peak areas and a larger ECSA value accompanied with an enhancement in the activity based on geometric current densities at 10 mA/cm^2 for $Ni_{7.5}Fe_1Cr_1$ LDH^{CO}_{MT} compared to that of $Ni_{7.5}Fe_1Cr_1$ LDH.

Conclusion

From these two control tests, we conclude that 1) the preheating step affects the size of the crystalline domains but neither the redox behavior nor the ECSA. and 2) CO ligands have an impact during synthesis on the morphology, resulting in large ECSA values.

2. Discussion of the Fe-XANES Spectra obtained at ESRF

Supplementary Figure 14 presents the recorded XAS Fe-K-edge XANES spectra. Panel *a)* illustrates the reference material NiFe LDH during potential stepping from 1.1 V to 1.9 V. At elevated potentials, a significant shift in white line intensity is observed, and at the highest potential (1.9 V), a pronounced pre-edge peak emerges. The data quality is notably compromised due to the lower concentration of Fe compared to Ni in NiFe LDH, particularly at higher potentials, where bubble formation further impedes accurate data acquisition. *Supplementary Figures 14 b)* and *c)* provide a comparison of the various NiFeX LDH samples examined in this study at 1.1 V and 1.9 V respectively. It is noteworthy that no substantial differences between the NiFeX catalysts are observed. Apparent is, that for all catalysts (including the reference material NiFe LDH) that an increase of the potential to 1.9 V leads to a reduction of the White Line intensity, which for K-edge spectra is difficult to interpret, as K-edge XAS measures the s → p transition, which is not necessarily directly influenced by the oxidation state of the absorber, because it is not probing the partially filled d-band states. If there was p-d hybridization we would be able to conclude an increase in empty states and could indicate a higher oxidation state. Further research, including X-Ray emission spectroscopy is needed to fully understand the role of Fe in NiFe as well as NiFeX LDHs.

3. Discussion of the Cell used in the XAS Experiments at ID26

Supporting Figure 12 a) shows a picture of the assembled cell in its horizontal configuration. For the experiments performed here the cell was mounted vertically. The diagram in *Supporting Figure 12 b)* shows the general functionalities of the cell. The cell consists of a glassy carbon rod inserted in the middle of the cell that is spanned with a mylar® foil. By increasing or decreasing the height of the electrolyte reservoir used for the electrolyte flow, we can change the internal pressure of the cell and thereby can control the formation of an electrolyte filled dome above the catalyst layer of interest. The electrolyte inlet is carefully positioned to directly hit small channels

laser-cut into the glassy carbon disks and has a small diameter to allow for maximized electrolyte velocity at the point of exit. These two design decisions lead to mitigated bubble formation on top of the catalyst layer while at the same time allowing a constant electrolyte film on top of the electrode. The cell is furthermore equipped to allow the introduction of a reference electrode as well as a counter electrode into the same compartment. We note that in this cell design the formation of the electrical field between the counter and working electrode is uncontrolled. Special care was taken to apply the catalyst layer only in the middle of the glassy carbon disk to mitigate the risk of currents being collected from 'invisible' (to the X-Ray beam) catalyst particles. While the currents achieved in this cell are far from the ones even obtained in RDE-Measurements, we are still confident that the changes in the observed XAS spectra are potential dependent as further evidenced by the presence of Ni oxidation/reduction peaks in the electrochemistry obtained during the XAS experiments (*Supplementary Figure 19*).

4. Beam Damage and Mitigation thereof

Beam damage is a significant problem for in situ measurements, especially at high brilliance facilities like the ESRF. Before the XAS experiments started, we conducted test measurements on pressed pellets to see whether the beam damage was present. These test measurements consisted of six 20 second illuminations of the same sample spot after each other. Comparing the fluorescence results of each one of them between each other, no change in the fluorescence spectrum would indicate no beam damage during this time. For all dry catalyst pellets no beam damage was found when probing the Ni-K-edge, while for probing the Fe-K-edge beam damage was observed. We repeated the tests for damage once more for the catalyst coated glassy carbon rods with added electrolyte in the cell, and no beam damage was found for either the Ni-K-edge or the Fe-K-edge for all samples. We further mitigated beam damage, by automatically moving to a new spot on the sample after every scan (~30 seconds).

5. Supplementary Discussion on the NiFeV LDH DFT Calculations

To identify the possible incorporation of V dopant into the catalytic active γ-NiFe LDH under OER conditions, we first examined the thermodynamic stability and dopant distribution in bulk ternary γ-NiFeV LDH. An Fe:X ratio of 1:1 was considered, which corresponds to a composition of $Ni_6FeVK_2O_{16}\cdot 4H_2O$. The free energy of formation (ΔG_{form}) is calculated based on the following reaction:

$$3\,\mathrm{NiOOH}+3\,\mathrm{NiO_2}+\mathrm{FeO_2}+0.5\,\mathrm{V_2O_5}+1.5\,\mathrm{H_2O}_{(l)}+2\,\mathrm{K^+}+2\,\mathrm{OH^-} \rightarrow \mathrm{Ni_6FeVO_{16}K_2}\cdot 4\,\mathrm{H_2O} \tag{1}$$

then,

$$\Delta G_{\mathrm{form}} = \mu\left(\mathrm{Ni_6FeVO_{16}K_2}\cdot 4\,\mathrm{H_2O}\right) - \Big\{3\mu\left(\mathrm{NiOOH}\right) + 3\mu\left(\mathrm{NiO_2}\right) + \mu\left(\mathrm{FeO_2}\right) + 0.5\cdot\mu\left(\mathrm{V_2O_5}\right)$$

$$+ 1.5\,\mu\left(\mathrm{H_2O}\right) + 2\mu\left(\mathrm{K^+}\right) + 2\mu\left(\mathrm{OH^-}\right)\Big\} \tag{2}$$

Which can further be normalized by the total number of metal atoms in the layer, i.e., 8 in our case. Due to the challenges in describing ions in standard DFT calculations, the chemical potential of K^+ and OH^- are calculated based on the following reaction with a reaction free energy of -3.33 eV:

$$\mathrm{K_2O} + \mathrm{H_2O} \rightarrow 2\,\mathrm{K^+} + 2\,\mathrm{OH^-} \tag{3}$$

Then,

$$2\mu\left(\mathrm{K^+}\right) + 2\mu\left(\mathrm{OH^-}\right) = \mu\left(\mathrm{K_2O}\right) + \mu\left(\mathrm{H_2O}\right) - 3.33 \tag{4}$$

The calculated free energy of formation is 0.05 eV/M. This endothermic value indicates that γ-NiFeV LDH is slightly metastable with respect to the selected reference species, which further suggests potential V segregation and dissolution. Such a result is consistent with experimental observation of near-surface V leaching.

To examine possible distribution of Fe and V in the γ-NiFe LDH framework, we further considered two representative bulk arrangements with different relative atomic positions of Fe and V (see *Supplementary Figure 27*). The calculated energy difference between the two bulk arrangements is 26 meV/$Ni_6FeVK_2O_{16}\cdot 4H_2O$, suggesting that both bulk arrangements are nearly degenerate and therefore may be relevant when constructing surface models for investigating OER overpotential. V has an oxidation state of +5 for both configurations, while Fe is +4 and +5 for the most stable configuration and slightly less stable configuration, respectively. Such electronic structure modulation in the bulk provides the basis for understanding how dopants may influence the surface electronic structure, stability and OER intermediates adsorption.

The catalytic oxygen evolution reaction mechanism: Beginning with the elucidated bulk structures described above, we first evaluated the steady state surfaces through surface phase diagram. We then calculated the reaction free energy of the oxygen evolution based on the following four steps:

$$\begin{aligned} &4\,\mathrm{OH}^- + * \leftrightharpoons 3\,\mathrm{OH}^- + \mathrm{OH}^* + e^- \\ &\leftrightharpoons 2\,\mathrm{OH}^- + \mathrm{O}^* + \mathrm{H_2O} + 2e^- \\ &\rightarrow \mathrm{OH}^- + \mathrm{OOH}^* + \mathrm{H_2O} + 3e^- \\ &\rightarrow \mathrm{O_2} + 2\,\mathrm{H_2O} + 4e^- + * \end{aligned} \tag{5}$$

Consistent with our previous study[6], we adopted an edge-exposed (01-10)-type surface model for γ-NiFeV LDH and used the same terminology to describe the OER intermediates as surface oxygen species.
To understand the increase in the OER activity upon incorporation of V dopant into the catalytically active γ-NiFe LDH. We investigated two different surface models representatives of their corresponding bulk configurations (see *Main Figure 5*). The goal is to understand how the location of dopant influences the surface stability and the OER activity. In the first model, Fe is exposed at the surface while V is located in the third layer, hereafter referred to as the Fe-terminated surface. In the second model, V is exposed at the surface and Fe is located in the third layer, hereafter referred to as the V-terminated surface. These two models allow us to directly distinguish the catalytic role of surface V from the electronic modulation effects of V in the third layer. The calculated surface energies show that both surface terminations are nearly degenerate, with an energy difference of only 18 meV. This difference suggests that both the surface terminations are energetically favourable and may coexist. Therefore, we considered both cases for their role in OER activity. We will show that, while the two surfaces are nearly degenerate energetically, their electronic and catalytic behaviors are different.

To identify the catalytically relevant surface under OER conditions, we first constructed surface phase diagrams for the Fe-terminated and V-terminated γ-NiFeV LDH surfaces. Consistent with our previous study on γ-NiFe LDH[6], the edge-exposed (01-10) surface was selected as the representative catalytic surface. Different surface coverages, including H- and OH-saturated configurations were considered to determine the steady-state surface in equilibrium with the alkaline electrolyte. After identifying the stable surface phase from the surface phase diagram, the OER reaction free-energy profiles were calculated using a Mars van Krevelen-type mechanism, for which the reaction begins from the deprotonation of the surface OH species. Similar to γ-NiFe LDH, the oxidation of two-metal coordinated bridge OH moieties is more favorable than that of one-metal coordinated atop OH due to the synergy of the two nearest-neighbor metal sites in stabilizing the potential limiting OER reaction intermediates (O* radicals) by performing an O-bridged reaction center. Therefore, we have similarly focussed our discussion below on the synergistic bridge OH oxidation pathway.

For the Fe-terminated surface, the calculated surface phase diagram indicates that under OER conditions, the undercoordinated surface O atoms are saturated with H forming bridge OH species, and undercoordinated metal sites are saturated with atop OH species when the surface is in equilibrium with the electrolyte and in steady state (see *Supplementary Figure 28*). Following Mars van Krevelen-type mechanism, the highest reaction free energy barrier is 1.88 eV, which implies an overpotential ($\boldsymbol{\eta}$) of 0.65 V. The potential determining step (PDS) is the OH* deprotonation during the OER cycle which has the highest free energy barrier followed by OOH* deprotonation.

On the Fe-terminated γ-NiFeV LDH surface, bridge OH (Fe^{+4}-OH-Ni^{+4}) deprotonation at 1.88 V is accompanied by Fe^{+4} oxidation (characterized by change in the magnetic moment of Fe (see *Supplementary Figure 28*), whereas the oxidation state of Ni remains constant. Therefore, Fe-terminated surface indicated no improvement in the OER activity when compared to γ-NiFe LDH ($\boldsymbol{\eta}$=0.45 V) due to the different electronic structures of reaction centers: Fe^{+4}-OH-Ni^{+4} for γ-NiFeV LDH versus Fe^{+4}-OH-Ni^{+3} for γ-NiFe LDH.

For the V-terminated surface, while the steady-state configuration under OER condition is similar to that of Fe-terminated surface, i.e., all sites are saturated, O(top-V)+H_2O(top-Ni) configuration is more favourable than OH(top-V)+OH(top-Ni) (see *Supplementary Figure 29*). Thus, we considered OER based on this configuration. Following OER mechanism, the highest free energy barrier is 1.60 eV, which implies an overpotential ($\boldsymbol{\eta}$) of 0.37 V. The potential determining step (PDS) is the OH* deprotonation during the OER cycle which has the highest free energy barrier followed by OOH* deprotonation. The bridge OH (V^{+5}-OH-Ni^{+2}) deprotonation at 1.60 V is accompanied by Ni^{+2} oxidation (characterized by change in the magnetic moment of Ni, see *Supplementary Figure 29*), whereas the oxidation state of V remains constant. Therefore, the V-terminated surface indicated improvement in the OER activity by decreasing the overpotential by 0.08 V compared to γ-NiFe LDH. This improvement in the overpotential is semi-quantitatively consistent with the present measurement indicating an overpotential shift of ~27 mV compared to reference γ-NiFe LDH catalysts, showing overpotentials close to 350 mV at 10 mA/cm^2.

Supplementary References

(1) Klingenhof, M.; Hauke, P.; Brückner, S.; Dresp, S.; Wolf, E.; Nong, H. N.; Spöri, C.; Merzdorf, T.; Bernsmeier, D.; Teschner, D.; et al. Modular Design of Highly Active Unitized Reversible Fuel Cell Electrocatalysts. *ACS Energy Letters* **2021**, *6* (1), 177-183. DOI: 10.1021/acsenergylett.0c02203.

(2) Dionigi, F.; Zhu, J.; Zeng, Z.; Merzdorf, T.; Sarodnik, H.; Gliech, M.; Pan, L.; Li, W.-X.; Greeley, J.; Strasser, P. Intrinsic Electrocatalytic Activity for Oxygen Evolution of Crystalline 3d-Transition Metal Layered Double Hydroxides. *Angewandte Chemie International Edition* **2021**, *60* (26), 14446-14457. DOI: https://doi.org/10.1002/anie.202100631.

(3) Jeon, S. S.; Kang, P. W.; Klingenhof, M.; Lee, H.; Dionigi, F.; Strasser, P. Active Surface Area and Intrinsic Catalytic Oxygen Evolution Reactivity of NiFe LDH at Reactive Electrode Potentials Using Capacitances. *ACS Catalysis* **2023**, *13* (2), 1186-1196. DOI: 10.1021/acscatal.2c04452.

(4) Strobl, O.; Martić, N.; Michaels, H.; de Godoy, F. N.; Wessnick, F.; Lang, D.; Kress, M.; Maltenberger, A.; Wengler-Rust, S.; Luxbacher, T.; et al. Low hydrogen crossover anion-exchange membrane water electrolysis based on non-ionic binder polymers. *EES Catalysis* **2026**, 10.1039/D6EY00083E. DOI: 10.1039/D6EY00083E.

(5) De Marco, G.; Malkow, T.; Pilenga, A.; Tsotridis, G. *EU harmonised polarisation curve test method for low-temperature water electrolysis*; Publications Office, 2018. DOI: doi/10.2760/179509.

(6) Dionigi, F.; Zeng, Z.; Sinev, I.; Merzdorf, T.; Deshpande, S.; Lopez, M. B.; Kunze, S.; Zegkinoglou, I.; Sarodnik, H.; Fan, D.; et al. In-situ structure and catalytic mechanism of NiFe and CoFe layered double hydroxides during oxygen evolution. *Nature Communications* **2020**, *11* (1), 2522. DOI: 10.1038/s41467-020-16237-1.
